# Multi-omics atlas of the bovine coronavirus-infected calf jejunum: reduction of *Phocaeicola coprophilus* and deoxycholic acid linked to Th17/Treg imbalance

**DOI:** 10.1038/s41522-026-00997-7

**Published:** 2026-05-07

**Authors:** Zheng Niu, Jingyi Xu, Xinfeng Hou, Guanglei Liu, Junhu Yao, Yangchun Cao, Qian Du, Dewen Tong, Shengru Wu

**Affiliations:** 1 College of Veterinary Medicine, Northwest A&F University, Yangling, China; 2 College of Animal Science and Technology, Northwest A&F University, Yangling, China; 3 National Center of Technology Innovation for Dairy, Hohhot, China; 4 JUNLEBAO-Northwest A&F University Cooperation Dairy Research Institute, Leyuan Animal Husbandry, JUNLEBAO Company, Shijiazhuang, China

**Keywords:** Diseases, Immunology, Microbiology

## Abstract

Optimal calf rearing is fundamental for ensuring efficient milk production. Bovine coronavirus (BCoV) poses a significant threat to calf health and leads to substantial economic losses in the dairy industry. However, the mechanisms by which the intestinal mucosal microbiome regulates the host immune response during infection remain unclear. In this study, we constructed a high-resolution map of the jejunal mucosal microenvironment in BCoV-infected calves. Our findings revealed that BCoV infection led to severe microbial dysbiosis, characterized by a marked reduction in *Phocaeicola coprophilus* (formerly known as *Bacteroides coprophilus*) and decreased secondary bile acid, especially deoxycholic acid (DCA). Concurrently, enrichment of harmful microbiota correlated with increased arachidonic acid metabolites. At the host level, BCoV infection altered the composition of jejunal mucosal cells and affected metabolic and immune-related pathways. The differentiation of CD4⁺ T cells played a central role in the jejunum’s response to BCoV infection. By integrating these metabolic alterations with dynamic host cellular responses, we suggested a putative that DCA deficiency might contribute to the pathological polarization of CD4^+^ T cells toward a Th17 phenotype while suppressing Treg differentiation. These findings suggest that restoring the *Phocaeicola coprophilus*-affected bile acid transformation might represent a promising therapeutic strategy for BCoV infection.

## Introduction

As a prevalent communal disease among ruminants, bovine coronavirus (BCoV) poses a serious threat to ruminant health and the development of the livestock industry, with reports indicating its potential risk for zoonotic transmission^1^. The BCoV infection rate is 30.6% in China and 52% in Europe, with the highest prevalence rate reaching 60.5%, with severe economic losses to dairy farms caused by transmission, making it one of the key diseases hindering the development of the dairy cattle industry^2–4^. BCoV can be transmitted through respiratory droplets via the fecal‒oral route, causing both digestive tract disease, characterized primarily by diarrhea, and respiratory illnesses^5^. Adult dairy cows are more prone to winter diarrhea when infected with BCoV, whereas calves remain susceptible throughout the year^1,2^. Previous studies have shown that when adult dairy cows are infected with BCoV, milk production decreases significantly or ceases overall, and compared with that of healthy cows during the same period, milk production decreases by up to 51 kg^2^. In calves, BCoV infection not only has severe health impacts via pronounced diarrhea and intestinal hemorrhage but also compromises milk production efficiency in later lactation stages, even post-recovery^6^. Therefore, studying prevention and treatment plans for BCoV is highly important for ensuring the efficient development of the dairy industry.

The mucosal surface serves as the body’s first line of defense against the external environment. Viral infections typically cause significant disruption of the mucosal barrier, triggering a series of pathological changes and host responses^7,8^. After BCoV infection, the virus first replicates throughout the small intestine, causing villus atrophy, tight junction disruption, and mucin layer depletion^9^. Afterward, it spreads throughout the entire large intestine, reaching the terminal colon and rectum^1^. BCoV-infected cows show hallmark lesions of the shortening and fusion of villi, denudation and infiltration of mononuclear cells in the lamina propria^10^. Damage to the intestinal epithelium reduces the absorptive surface area, allowing unabsorbed nutrients to draw water into the intestinal lumen through osmosis, causing malabsorptive diarrhea^11^. Such mucosal damage triggers rapid and extensive host responses, including the activation of innate immune pathways, the recruitment of inflammatory cells, and alterations in cytokine networks^12,13^. A study revealed that inoculation of mice with a virus expressing the bovine coronavirus spike protein resulted in elevated levels of specific IgG, along with increased populations of B lymphocytes, CD8^+^ T lymphocytes, and CD4^+^ T lymphocytes in the peripheral circulation^14^. An in vitro model study further demonstrated that IFN-α and IFN-β are persistently produced during BCoV infection, indicating sustained activation of the host innate immune response. The distinct expression patterns of the proinflammatory cytokine IL-6 and the anti-inflammatory cytokine IL-10 highlight the complexity of the host’s immunomodulatory mechanisms^15^. However, after being infected with BCoV, how the intestinal cells of dairy cows respond to virus invasion and which immune cells function have not been fully explored.

Accumulating evidence indicates that the host–microbiome axis plays an essential regulatory role during viral infections^16–18^. In the context of enteric viral infections, the gut microbiome may either inhibit viral replication or infection through metabolic byproducts, such as indole-3-propionic acid produced by *Clostridium sporogenes*, as well as *Lactobacillus* and *Bifidobacteriaceae* species, decreasing the viral load^19–21^. More importantly, the gut microbiome, especially probiotics, can increase host resistance by promoting barrier repair and regulating host immune cells to produce cytokines^22^. Previous studies have shown that SCFAs derived from gut microbial metabolic activity can upregulate IL-22 production by promoting aryl hydrocarbon receptor and hypoxia-inducible factor 1α expression and that microbially derived secondary bile acids—such as LCA, iso LCA and DCA—increase host resistance by modulating T-cell differentiation and promoting epithelial barrier repair^23,24^. Thus, disruptions in microbial community structure and enrichment of beneficial microbiomes are increasingly recognized as key determinants of infection severity and recovery dynamics. However, whether BCoV infection causes microbial disruption and how the gut microbiome can improve the host response to BCoV infection are entirely unknown.

After BCoV infection, the jejunum has the highest viral load and is the most severely damaged intestinal segment^10,25^. Additionally, the jejunum is often selected as a representative site for mucosal interaction because it is the primary site for both nutrient absorption and enteric pathogen‒host interactions^26,27^. Therefore, our research aimed to explore the microbiome and its metabolic alterations and how the jejunal mucosal cell type and proportion change after BCoV infection, as well as the potential probiotics that may prevent or alleviate BCoV infection. Our results revealed that after BCoV infection, microbial arachidonic acid metabolism is activated, releasing proinflammatory metabolites to accelerate further inflammation. *Phocaeicola coprophilus* (formerly known as *Bacteroides coprophilus*) is associated with secondary bile acid biosynthesis to potentially mediate the differentiation of CD4^+^ T cells into Treg cells, alleviating the inflammatory response and maintaining immune stability.

## Results

### BCoV infection disrupted intestinal morphology and affected glucose and lipid metabolism

**Fig. 1:**
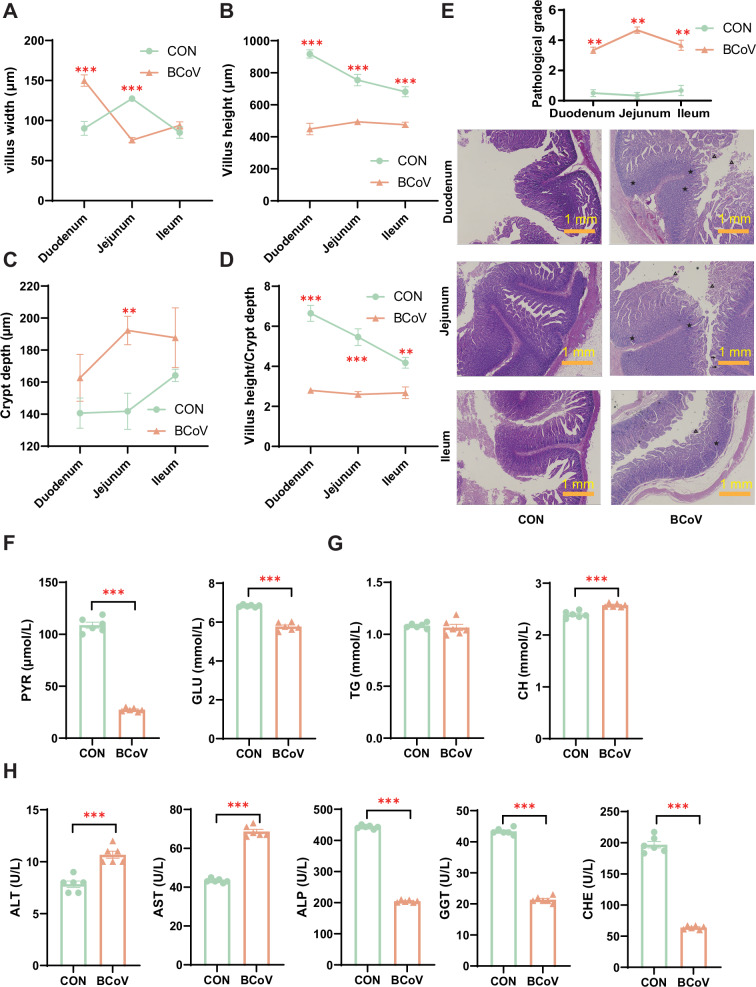
Effects of BCoV infection on intestinal histology and blood biochemical indicators. **A–D** Statistical results of histological examinations of various intestinal segments, including the duodenum, jejunum and ileum, including villus weight (**A**), villus height (**B**), crypt depth (**C**) and the distance from the villus height to crypt depth (**D**). **E** HE staining was used for morphological examination of various intestinal segments. Scale bars: left, 2.5; right, 10. The asterisks indicate abnormal proliferation of crypts, the triangles indicate villus shedding or atrophy, and the arrows indicate focal hemorrhage. **F–H** Blood biochemical indicators, including PYR, GLU (**F**), TG, CH (**G**), ALT, AST, ALP, GGT, and CHE (**H**), were measured. The data are presented as the means ± SEMs. *P < 0.05, **P < 0.01, ***P < 0.001.

The intestinal morphology of calves significantly changed after BCoV infection. The villus width in the duodenum increased significantly but decreased significantly in the jejunum after BCoV infection (*P* < 0.001; Fig. 1A). The villus height was significantly lower in BCoV-infected calves than in control calves in the duodenum, jejunum and ileum (*P* < 0.001; Fig. 1B). Compared with that of healthy calves, the crypt depth of BCoV-infected calves was significantly greater only in the jejunum (*P* < 0.01; Fig. 1C). BCoV infection led to a significant decrease in the ratio of the villus height to crypt depth in the small intestine (*P* < 0.01; Fig. 1D). Observations of intestinal morphology revealed that after infection with BCoV, there was atrophy and shedding of the intestinal villi, abnormal proliferation of the crypts, structural disorders of the intestinal mucosal layer, and obvious focal hemorrhage in the jejunal tissue (Fig. 1E). In that case, a statistical analysis of the pathological grades of three intestinal segments in the two groups was conducted, which showed that whether it was the duodenum, jejunum or ileum, the pathological score of the BCoV group was significantly higher than that of the CON group (*P* < 0.01; Fig. 1E).

The concentrations of pyruvate and glucose decreased significantly in BCoV, suggesting a disorder of blood glucose metabolism (*P* < 0.001; Fig. 1F). There were no significant differences in triglyceride levels (*P* > 0.05; Fig. 1G), but the concentration of cholesterol markedly increased in BCoV-infected calves, suggesting negative effects on lipid digestion and absorption (*P* < 0.001; Fig. 1G). Alanine aminotransferase and aspartate aminotransferase levels were significantly elevated in the BCoV group, whereas alkaline phosphatase, γ-glutamyl transferase, and cholinesterase levels were markedly reduced, indicating hepatic cell injury and impaired liver function (*P* < 0.001; Fig. 1H).

### Changes in the microbial composition of the jejunum mucosa after BCoV infection

**Fig. 2:**
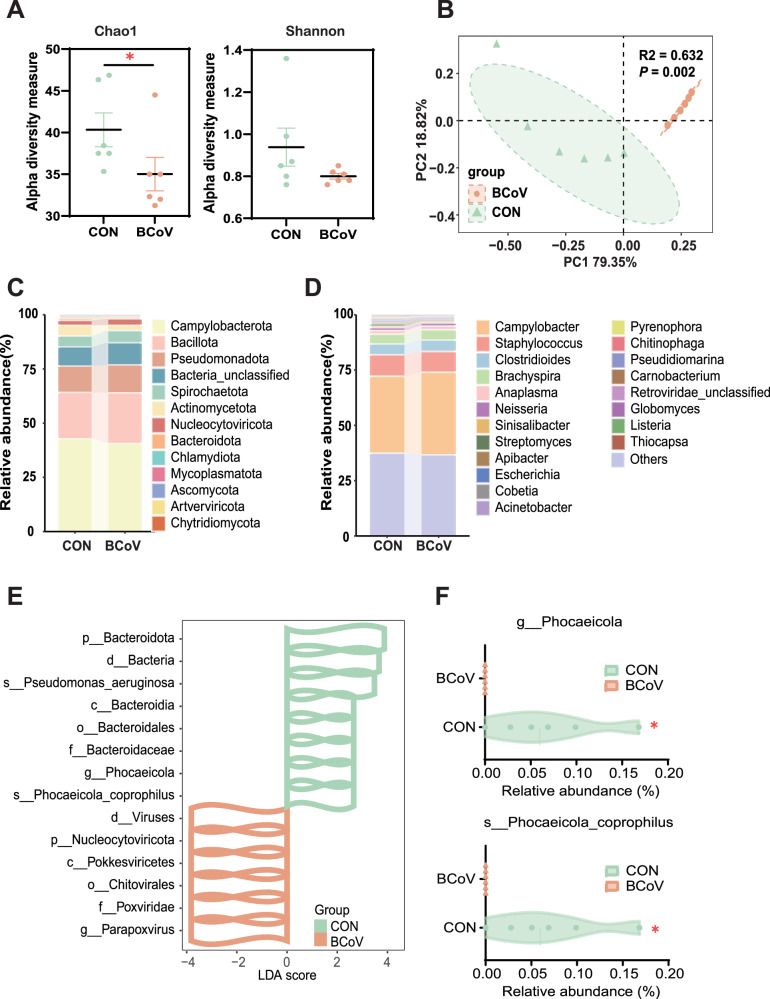
The microbial composition and differences in the jejunal mucosa between BCoV-infected and healthy calves. **A** Differences in α diversity between BCoV and CON, including the Chao1 index and Shannon index. **B** β diversity analysis via PCoA with statistics performed via ANOSIM. **C**, **D** The composition of the microbiome at the phylum level (**C**) and genus level (**D**). **E** Differences in the LEfSe of intestinal mucosal microorganisms between the BCoV-infected and CON groups. **F** Differences in the abundance of *Phocaeicola* and *Phocaeicola coprophilus* in the jejunal mucosa between the BCoV group and the CON group according to the Mann‒Whitney U test.

The Chao1 index and Sobs index were lower in BCoV-infected calves than in control calves, indicating a decrease in microbial richness (*P* < 0.05; Fig. 2A and Supplementary Fig. 1A), while there were no significant differences in the Shannon and Simpson indices (*P* > 0.05; Fig. 2A and Supplementary Fig. 1B). PCoA revealed significant separation of the jejunal mucosal microbiome between the BCoV and CON groups (*P* < 0.05; Fig. 2B). Thirteen phyla were identified, including Campylobacterota, Bacillota, Pseudomonadota, Spirochaetota, and Actinomycetota (Fig. 2C). Twenty genera with relative abundances greater than 1% in the jejunum mucosa were identified, including *Campylobacter*, *Staphylococcus*, *Clostridioides*, *Brachyspira*, *Anaplasma*, *Neisseria* and others (Fig. 2D).

LEfSe revealed that the relative abundances of domain bacteria, the phylum Bacteroidota, the class Bacteroidia, the order Bacteroidales, the family Bacteroidaceae, the genus *Phocaeicola* and the species *Pseudomonas aeruginosa* and *Phocaeicola coprophilus* significantly decreased in BCoV-infected calves (LDA > 2, *P* < 0.05; Fig. 2E). The relative abundances of domain viruses, the phylum Nucleocytoviricota, the class Pokkesviricetes, the order Chitovirales, the family Poxviridae and the genus *Parapoxvirus* increased significantly in BCoV-infected calves (LDA > 2, *P* < 0.05; Fig. 2E). Stamp analysis revealed that the abundances of the genus *Phocaeicola* and the species *Phocaeicola coprophilus* were significantly lower in BCoV-infected calves than in control calves (*P* < 0.05; Fig. 2F).

### Altered metabolic functions of the jejunal mucosal microbiome modulated the immune process

An exploration of the microbial function of the jejunum mucosa revealed that biosynthesis of secondary metabolites; biosynthesis of cofactors; purine metabolism; glycine, serine and threonine metabolism; and arachidonic acid metabolism were enriched (Supplementary Fig. 2A). These findings indicated that the microbiota in the jejunal mucosa was involved in the regulation of carbohydrate, lipid and protein metabolism. Compared with those in the BCoV group, the functional annotation–based GO enrichment analysis revealed that the healthy calves presented greater relative abundances of microbial genes enriched in defense responses to gram-negative and gram-positive bacteria, as well as positive regulation of T-cell cytokine production and T-cell–mediated cytotoxicity. In addition, functions related to the regulation of the defense response to viruses by viruses were also enriched, indicating the presence of microbial genes linked to antiviral response modulation. Moreover, multiple GO terms associated with antigen processing and presentation via MHC class I pathways (including both TAP-dependent and TAP-independent processes) were significantly different between the groups. These terms reflected microbial gene functions analogous to antigen presentation mechanisms (*P* < 0.05; Supplementary Fig. 2B and Supplementary Table 1).

### The ability of the microbiota to mediate the shift in lipid metabolism significantly changed after BCoV infection

**Fig. 3:**
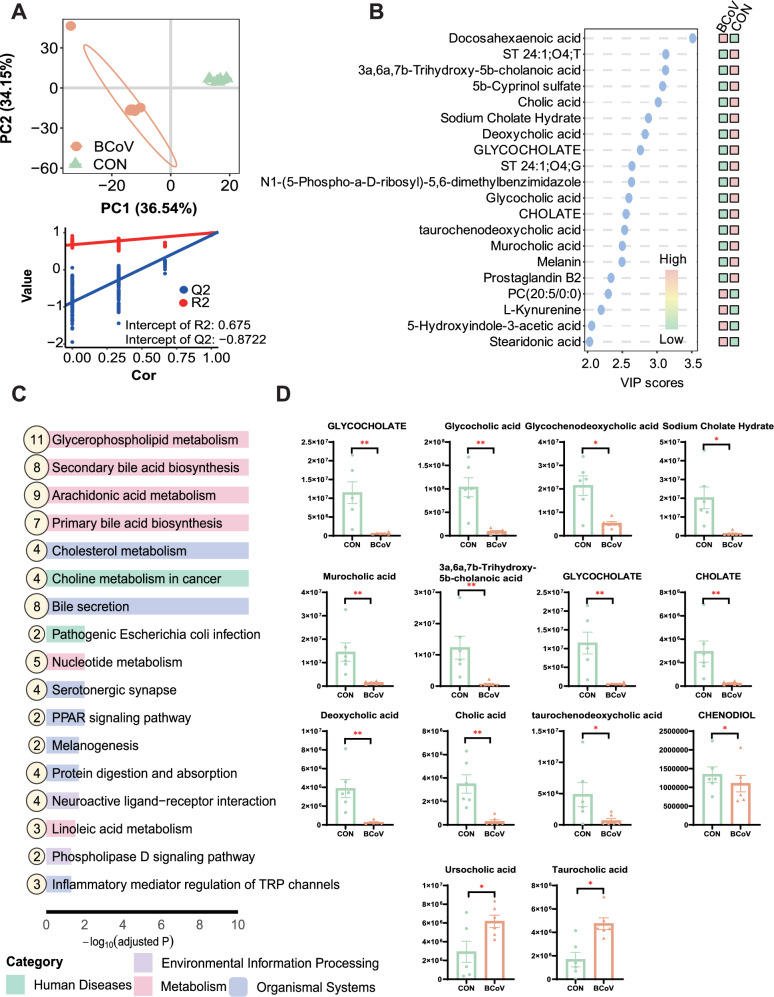
The composition of and differences in intestinal mucosal metabolites between BCoV and CON calves. **A** PLS-DA with a permutation test showing the differences in metabolites between the BCoV group and the CON group. **B** Heatmap of VIP scores for the top 20 differentially abundant metabolites. **C** KEGG pathway enrichment analysis of differentially abundant metabolites. **D** Box plot of the differentially abundant metabolites involved in secondary bile acid synthesis between the BCoV and CON groups.

A nontargeted metabolomics analysis focused on the changes in metabolites in the jejunum mucosa. Partial least squares discriminant analysis revealed that the metabolites of BCoV-infected calves and healthy calves were significantly different (*P* < 0.05; Fig. 3A). The permutation test indicated that the model had a good fit and predictive ability (Fig. 3A). There were 239 metabolites that were significantly different between the BCoV and CON groups, among which 140 metabolites were upregulated and 99 metabolites were downregulated in BCoV-infected calves (*P* < 0.05; Supplementary Fig. 3A, B). A screening of the top 20 VIP score metabolites revealed that most of them were lipid metabolites, especially those in the bile acid family, such as cholic acid, sodium cholate hydrate and deoxycholic acid (*P* < 0.05; Fig. 3B). The KEGG enrichment analysis revealed that glycerophospholipid metabolism, secondary bile acid biosynthesis, arachidonic acid metabolism, primary bile acid biosynthesis and other metabolic pathways were significantly enriched (*P* < 0.05; Fig. 3C). The levels of most of the differentially abundant metabolites of the bile acid family, including GCA (glycocholate and glycocholic acid) and secondary bile acid DCA (deoxycholic acid), were significantly greater in the CON group than in the BCoV group (*P* < 0.05; Fig. 3D). However, the levels of UCA (ursocholic acid) and TCA (taurocholic acid) were significantly greater in BCoV-infected calves (*P* < 0.05; Fig. 3D).

In contrast, the levels of most metabolites involved in arachidonic acid metabolism, including TXB2, 11Beta-Prostaglandin F2Alpha, 8-HETE, 5(S)-HETE and others, increased significantly in BCoV-infected calves (*P* < 0.05; Supplementary Fig. 4A). Metagenomic analysis revealed the microbial functional enrichment of arachidonic metabolism, and in that case, correlation analysis between metabolites involved in arachidonic metabolism and the microbiome was conducted. The increase in *Streptococcus anginosus* in BCoV-infected calves was significantly positively correlated with the proinflammatory metabolites TXB2, 11Beta-Prostaglandin F2Alpha and 5(S)-HETE (*P* < 0.05; Supplementary Fig. 4B and Supplementary Table 2). While the number of *Phocaeicola coprophilus* significantly decreased in BCoV-infected calves, it was strongly negatively correlated with the proinflammatory metabolites TXB2, 11Beta-Prostaglandin F2Alpha, 8-HETE and 5(S)-HETE (*P* < 0.05; Supplementary Fig. 4B and Supplementary Table 2).

### Construction of the calf jejunum mucosal cell atlas

**Fig. 4:**
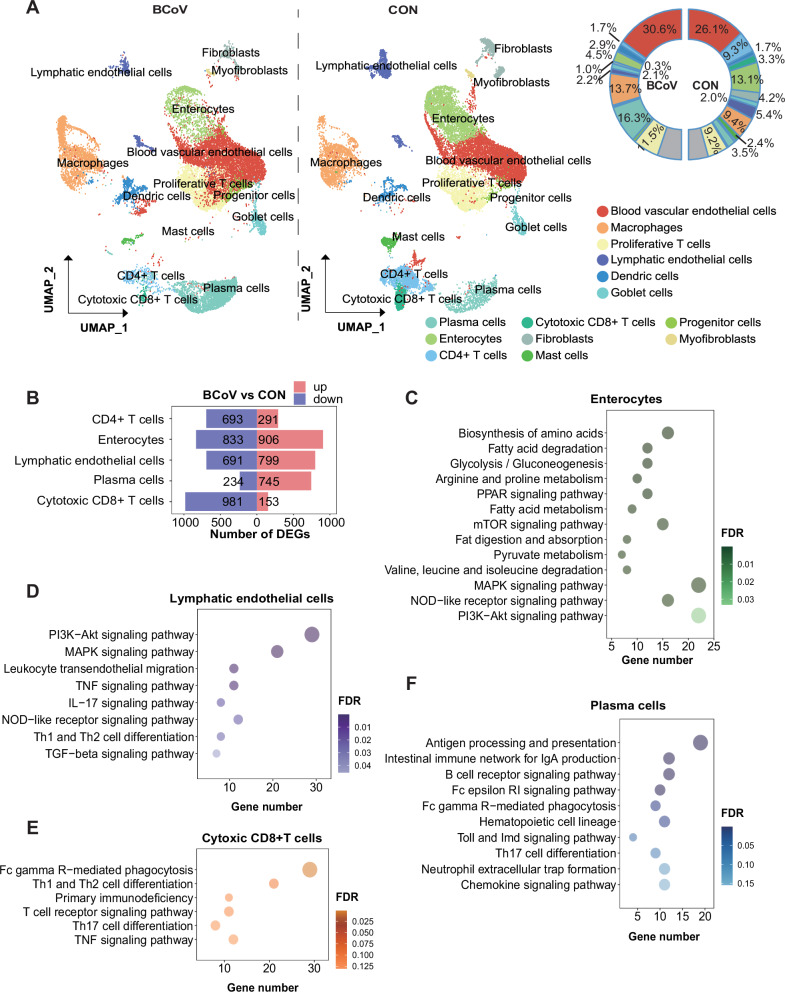
Results of single-cell transcriptome analysis of jejunum tissue and KEGG functional enrichment analysis of various cell types. **A** UMAP plot showing the cell composition of jejunal tissues in the BCoV and CON groups. **B** Statistical analysis of the number of differentially expressed genes (DEGs) for each cell type. **C** KEGG enrichment analysis revealed significantly different pathways in enterocytes between the BCoV and CON groups. **D** KEGG enrichment analysis revealed significantly different pathways in lymphatic endothelial cells between the BCoV and CON groups. **E** KEGG enrichment analysis revealed significantly different pathways in cytotoxic CD8^+^ T cells between the BCoV and CON groups. **F** KEGG enrichment analysis revealed significantly different pathways in plasma cells between the BCoV and CON groups.

To investigate changes in cell types and cellular composition following BCoV infection, single-cell RNA sequencing was performed on jejunal mucosal tissues. A total of 57,176 cells from four samples were analyzed, with an average of 14,294 cells per sample. On average, 1762 median genes were detected per cell. A total of 57,176 cells were generated into 32 clusters, and the expression profiles of the top three genes are shown for each cluster (Supplementary Fig. 5A, B). Fourteen types of cells were identified: blood vascular endothelial cells, CD4^+^ T cells, dendritic cells, cytotoxic CD8^+^ T cells, enterocytes, fibroblasts, goblet cells, lymphatic endothelial cells, macrophages, mast cells, myofibroblasts, plasma cells, progenitor cells, and proliferative T cells (Fig. 4A). Among them, the proportions of enterocytes, lymphatic endothelial cells, CD4^+^ T cells, and cytotoxic CD8^+^ T cells significantly decreased, and the proportion of plasma cells significantly increased after BCoV infection (*P* < 0.05; Fig. 4A and Supplementary Table 3). An exploration of the DEGs revealed that after BCoV infection, 291 genes were upregulated and 693 genes were downregulated in CD4^+^ T cells, 906 genes were upregulated and 833 genes were downregulated in enterocytes, 799 genes were upregulated and 691 genes were downregulated in lymphatic endothelial cells, 745 genes were upregulated and 234 genes were downregulated in plasma cells, and 153 genes were upregulated and 981 genes were downregulated in cytotoxic CD8^+^ T cells (*P* < 0.05; Fig. 4B).

The KEGG enrichment analysis of the DEGs in enterocytes revealed that the biosynthesis of amino acids, fatty acid degradation, glycolysis/gluconeogenesis, arginine and proline metabolism, fatty acid metabolism, fat digestion and absorption and valine, leucine and isoleucine degradation were enriched, indicating that changes in glycometabolism, lipid metabolism and amino acid metabolism occurred (*P* < 0.05; Fig. 4C and Supplementary Table 4). Additionally, the mTOR signaling pathway, MAPK signaling pathway and PI3K-Akt signaling pathway were enriched, indicating changes in cell proliferation and growth (*P* < 0.05; Fig. 4C and Supplementary Table 4).

After BCoV infection, the response of jejunal mucosal immune cells also changed. The KEGG enrichment of DEGs in lymphatic endothelial cells revealed that leukocyte trans-endothelial migration, the TNF signaling pathway, the IL − 17 signaling pathway, Th1 and Th2 cell differentiation, and the TGF−beta signaling pathway were enriched (*P* < 0.05; Fig. 4D and Supplementary Table 5). The results of the KEGG enrichment analysis of DEGs in cytotoxic CD8^+^ T cells revealed that genes related to Fc gamma R-mediated phagocytosis, Th1 and Th2 cell differentiation, primary immunodeficiency, the T-cell receptor signaling pathway, Th17 cell differentiation, and the TNF signaling pathway were enriched (*P* < 0.05; Fig. 4E and Supplementary Table 6). The KEGG enrichment of DEGs in plasma cells revealed that antigen processing and presentation, the intestinal immune network for IgA production, the B-cell receptor signaling pathway and other immune-related pathways were enriched (*P* < 0.05; Fig. 4F and Supplementary Table 7).

### Central involvement of CD4⁺T-cell differentiation in the jejunal response to BCoV infection

**Fig. 5:**
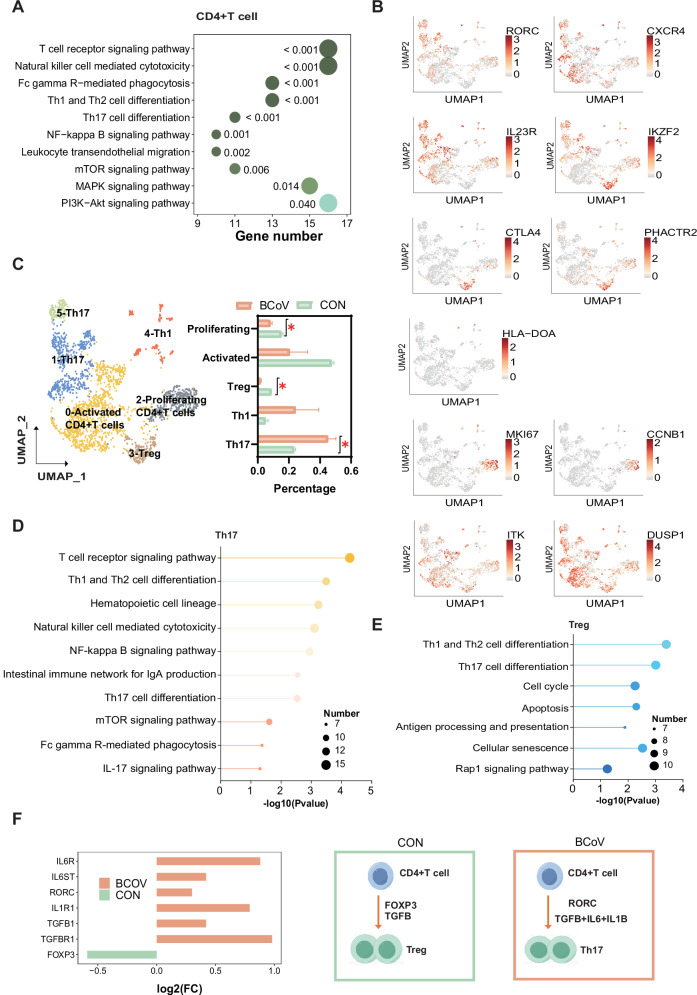
The results of subset analysis of CD4^+^ T cells in jejunal tissues and KEGG functional enrichment analysis. **A** KEGG enrichment analysis revealed significantly different pathways in CD4^+^ T cells between the BCoV and CON groups. **B** Cell markers of different CD4^+^ T cells: *RORC*, *CXCR4*, and *IL-23R* for Th17 cells; *IKZF2*, *CTLA4*, and *PHACTR2* for Treg cells; *HLA-DOA* for Th1 cells; *MKI67* and *CCNB1* for proliferating CD4^+^ T cells; and *ITK* and *DUSP1* for activated CD4^+^ T cells. **C** UMAP plot and statistical results showing the cell composition of CD4^+^ T cells in the BCoV and CON groups. **D** KEGG enrichment analysis revealed significantly different pathways in Th17 cells between the BCoV and CON groups. **E** KEGG enrichment analysis revealed significantly different pathways in Tregs between the BCoV and CON groups. **F** Differences in Th17 and Treg differentiation and key cytokine differences between BCoV and CON jejunal tissues.

The KEGG enrichment of DEGs in CD4^+^ T cells revealed that the T-cell receptor signaling pathway, Th1 and Th2 cell differentiation, Th17 cell differentiation and other pathways were enriched (*P* < 0.05; Fig. 5A and Supplementary Table 8). To explore the differentiation of CD4^+^ T cells, sub-cell types were identified. The high expression of *RORC*, *CXCR4*, and *IL-23R* suggested that cluster 1 and cluster 5 represented T helper 17 (Th17) cells. The high expression of *IKZF2*, *CTLA4*, and *PHACTR2* implied that cluster 3 was a T regulatory (Treg) cell. The high expression of *HLA-DOA* suggested that cluster 4 was T helper 1 (Th1) cells. The high expression of *MKI67* and *CCNB1* implied that cluster 2 represented proliferating CD4^+^ T cells, and the high expression of *ITK* and *DUSP1* implied that cluster 0 represented activated CD4^+^ T cells (Fig. 5B, C).

Statistical analysis of the proportions of different CD4^+^ T-cell subtypes revealed that the proportions of proliferating CD4^+^ T cells and Tregs were significantly greater in the CON group, whereas the proportion of Th17 cells was significantly greater in the BCoV group (*P* < 0.05; Fig. 5C and Supplementary Table 9). KEGG enrichment analysis of the DEGs in Th17 cells revealed that the T-cell receptor signaling pathway, Th17 cell differentiation pathway and IL-17 signaling pathway were enriched (*P* < 0.05; Fig. 5D and Supplementary Table 10). The KEGG enrichment analysis of DEGs in Tregs revealed that Th1 and Th2 cell differentiation, Th17 cell differentiation and antigen processing and presentation were enriched (*P* < 0.05; Fig. 5E and Supplementary Table 11). Gene expression analysis of Th17 and Treg cells revealed that the expression of *IL6R*, *IL6ST*, *RORC*, *IL1R1*, *TGFB1*, and *TGFBR1* was upregulated in the BCoV group, whereas that of *FOXP3* was upregulated in the CON group (Fig. 5F). These findings indicate that BCoV infection suppresses the differentiation of CD4^+^ T cells through the upregulation of *IL-6*, *IL-1*, and *TGF-β* signaling and the Th17-associated transcription factor *RORC* coupled with the downregulation of the Treg-specific transcription factor *FOXP3*, thereby inhibiting Treg development and promoting Th17 polarization (Fig. 5F).

### Interplay between the jejunal microbial metabolism and Th17/Treg cell proportions

**Fig. 6:**
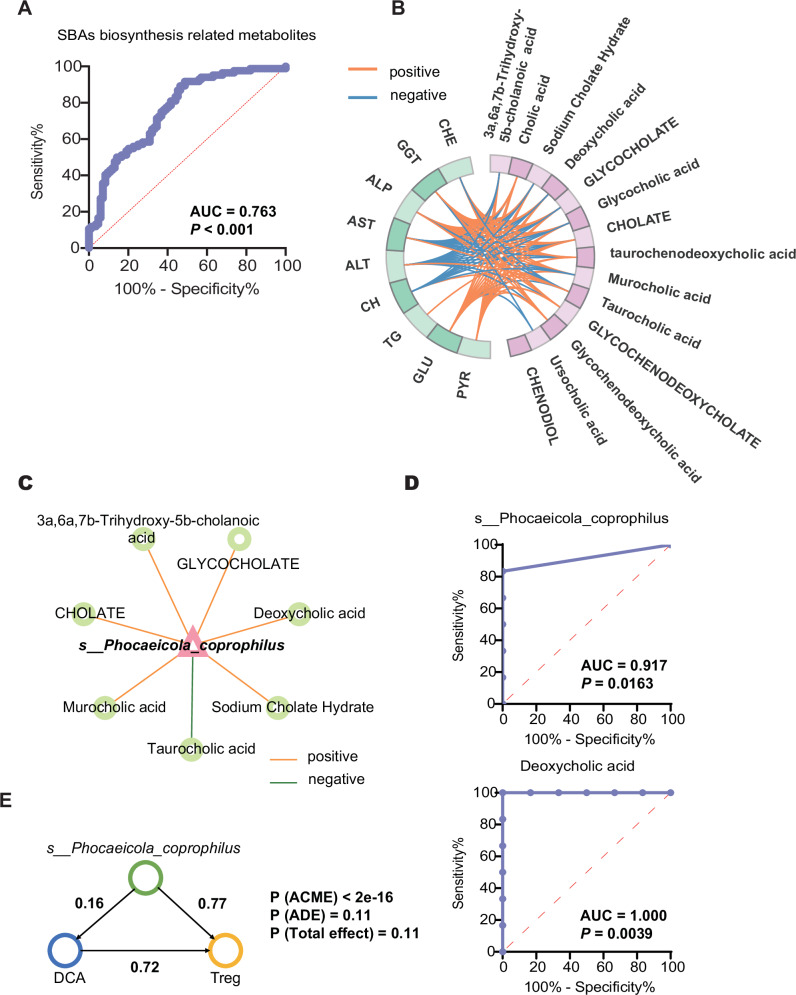
Multiomics analysis revealed microbiota–metabolite–immune cell alterations. **A** ROC diagnostic curve of metabolites related to secondary bile acid biosynthesis. **B** Correlation analysis of metabolites and biochemical indicators. **C** The correlation network between the microbiota and secondary bile acid metabolites. **D** ROC diagnostic curves of *Phocaeicola coprophilus* and DCA. **E** Mediation analysis model of microbiota-metabolite-immune cells.

The results of the receiver operating characteristic (ROC) curve indicated that metabolites with significant differences in secondary bile acid metabolism could serve as biomarkers to effectively distinguish BCoV-infected calves from normal calves (AUC = 0.763, *P* < 0.001; Fig. 6A). Correlation analysis indicated that bile acids such as DCA were significantly associated with glucose metabolism-related indicators (GLU and PYR), lipid metabolism-related indicators (TG and CH), and liver function indicators (AST, ALT, and GGT) (*P* < 0.05; Fig. 6B). The significantly decreased abundance of the microbial species *Phocaeicola coprophilus* in the BCoV group was significantly positively correlated with GCA and DCA and significantly negatively correlated with TCA and MCA, indicating that it might play a key role in the metabolic regulation of secondary bile acids (*P* < 0.05; Fig. 6C). ROC analysis revealed that *Phocaeicola coprophilus* (AUC = 0.917; *P* = 0.0163) and DCA (AUC = 1.000; *P* = 0.0039) could serve as biomarkers to identify BCoV-infected and healthy calves (Fig. 6D).

To verify the correlation between *Phocaeicola coprophilus* and bile acid biosynthesis, a single bacterial genome analysis of *Phocaeicola coprophilus* was performed. A total of 3535 genes encoding 3352 proteins were identified. The KEGG annotation indicated that *Phocaeicola coprophilus* expressed bile salt hydrolase, which hydrolyzed conjugated bile acids into free bile acids, which is a necessary precursor for DCA production (Supplementary Table 12). Mediating effect analysis revealed that *Phocaeicola coprophilus* influenced the host phenotype through distinct immune cell pathways. The indirect effect of DCA on *Phocaeicola coprophilus* was highly significant (*P*_*ACME*_ < 0.001). However, neither the direct effect (ADE) nor the total effect reached statistical significance (*P* = 0.11), suggesting that this bacterium primarily regulated Treg differentiation via the mediating role of DCA (Fig. 6E).

## Discussion

Given the widespread prevalence of BCoV on large-scale dairy farms worldwide, which has caused substantial economic loss and a considerable burden on the global dairy industry, deciphering the complex interplay between the intestinal microbiota and host responses following BCoV invasion is urgently needed to identify methods for prevention and control^4,28^. To address this gap, this study investigated changes in blood biochemical indices and intestinal morphology and compared the composition and relative abundance of the jejunal mucosal microbiota, metabolites, and host cell populations between BCoV-infected diarrheic calves and healthy controls. The jejunum was selected as the primary site of investigation because of its pivotal role in both physiological function and disease pathogenesis. First, physiologically, the jejunum serves as the principal site of nutrient absorption in neonatal calves and constitutes a critical component of the intestinal mucosal immune system^26,27^. Second, from a pathogenic perspective, BCoV exhibits marked tropism for the jejunal epithelium. Histopathological evidence consistently demonstrates that compared with the duodenum and ileum, the jejunum sustains more severe lesions and robust viral replication^10,25^. Building on these insights, we further elucidated glucose and lipid metabolism disorders and intestinal barrier damage after BCoV infection. Importantly, the reshaping of the jejunal microenvironment manifested as a decrease in the relative abundance of *Phocaeicola coprophilus*, which was negatively related to arachidonic metabolism and positively related to secondary bile acid metabolism. These changes in microbial metabolism were further associated with an imbalance in the host immune system, manifested as an increase in the proportion of Th17 cells and a decrease in the proportion of Treg cells, exacerbating the damage caused by BCoV infection to the host.

Morphological examination of the intestinal mucosa revealed that BCoV infection induced structural damage to the mucosa across all segments of the intestine, especially jejunal mucosa damage, in the present study. Similarly, intestinal villus atrophy and crypt hyperplasia occur in other coronavirus infections, indicating that this is a typical characteristic of viral enteritis^29–31^. The intestine is the main site for nutrient absorption^32^. Damage to the intestinal mucosa reduces the absorption surface area, leading to insufficient intake of metabolic substrates such as glucose and amino acids, which usually leads to metabolism, secretion and absorption disorders, thereby causing malabsorptive diarrhea^33,34^. When affected by intestinal mucosa injury, the concentrations of glucose and pyruvate decrease, indicating energy metabolism disorders in organisms. Consistent with the observed disruption of intestinal morphology, our scRNA-seq data indicated a significant reduction in the proportion of enterocytes. As the main absorptive cells in the intestinal mucosa, enterocytes are responsible for the absorption of nutrients^35^. In addition to the decreased proportion of enterocytes, disorders in glycolysis, fatty acid metabolism, and amino acid biosynthesis in surviving enterocytes occurred after BCoV infection in our study, confirming that viral infection caused metabolic abnormalities in the host epithelium.

The intestinal microbiota and the intestinal epithelium contribute to the breakdown and synthesis of nutrients and regulate the expression of metabolic genes in the intestinal epithelium via the microbiota–intestinal axis^36,37^. As the intestinal segment with the most severe damage to the intestinal epithelium after BCoV infection, the microbial changes in the jejunum mucosa have attracted our attention. Our research revealed that the microbial richness of the mucosal layer decreased after infection with BCoV, indicating that viral invasion severely disrupted the stable microenvironment of the jejunum. Among them, we observed that *Phocaeicola coprophilus*, which normally exists in the intestinal mucosa of healthy calves, completely disappeared in the intestinal mucosa of calves infected with BCoV. *Phocaeicola coprophilus* harbors canonical polysaccharide utilization loci (PULs) and is capable of degrading dietary polysaccharides, thereby contributing significantly to mucosal layer renewal and host energy harvest^38^. *Phocaeicola coprophilus* was initially isolated from human feces and has demonstrated significant potential in the amelioration of ulcerative colitis and the management of gestational diabetes^39–41^. In a randomized clinical trial, the administration of *Phocaeicola coprophilus* following fecal microbiota transplantation from donors was strongly associated with clinical improvement in patients with ulcerative colitis^39^. Furthermore, alterations in the gut microbiota of pregnant individuals with diabetes are linked to metabolic inflammation and imbalances in short-chain fatty acid (SCFA) production, which may contribute to the development of gestational diabetes. Given its capacity to produce SCFAs, *Phocaeicola coprophilus* represents a promising probiotic candidate for the prevention and treatment of gestational diabetes^41^. Meanwhile, another study identified that probiotic supplementation with *Phocaeicola coprophilus* could inhibit repression of the IDO1-Kyn axis and mitigate intestinal fibrosis^42^. Similar to previous studies, our research revealed that *Phocaeicola coprophilus* could serve as a probiotic to alleviate diarrhea in calves caused by BCoV.

However, unlike previous studies, through correlation analysis, we found that *Phocaeicola coprophilus* may affect the host cell response by influencing bile acid metabolism and the production of secondary bile acids. Our multiomics analysis demonstrated that BCoV infection resulted in a significant reduction in the abundance of *Phocaeicola coprophilus*, which was positively associated with the level of DCA. DCA is an effective endogenous ligand of TGR5 and has been shown to attenuate the release of proinflammatory cytokines, including TNF-α, IL-6, and IL-1β, through the inhibition of the NF-κB signaling pathway^43–45^. Moreover, previous studies have reported that at low physiological concentrations, DCA can suppress the differentiation of CD4^+^ T cells into Th1 and Th17 cells, thereby contributing to the inhibition of inflammatory cytokine release and the modulation of inflammatory responses^46–48^. Therefore, maintaining a normal concentration of DCA is vital for preserving the internal environment of the intestine and maintaining an anti-inflammatory state. In contrast, viral infection established a microenvironment that favors the metabolic activity of opportunistic pathogens such as *Streptococcus anginosus*. Integrated evidence from metagenomic and metabolomic analyses demonstrated that enrichment of pathogenic bacteria promoted the activation of the arachidonic acid metabolic pathway. Arachidonic acid can be enzymatically oxidized via the oxidase system into various bioactive lipid mediators, which play pivotal roles in the initiation, amplification, and persistence of inflammatory responses^49,50^. Our study revealed significantly elevated levels of arachidonic acid metabolites, specifically TXB2 and 5(S)-HETE, in calves infected with BCoV. TXB2, a stable derivative of TXA2, serves as an indicator of enhanced cyclooxygenase (COX)-mediated proinflammatory signaling and heightened immune activation^51,52^. 5(S)-HETE functions as both a chemotactic and an activating signal for leukocytes and can be further metabolized into 5-oxo-ETE, a more potent chemoattractant, thereby exacerbating inflammatory processes^53,54^. Arachidonic acid metabolites, as potent lipid signaling molecules, might facilitate Th17 polarization via autocrine or paracrine mechanisms^55,56^. These findings, manifested as the downregulation of DCA alongside increased production of proinflammatory arachidonic acid derivatives, indicate that BCoV-induced gut microbiota dysbiosis not only results in reduced microbial diversity but also leads to impaired metabolic function.

After single-cell RNA analysis, changes in cell types and proportions in jejunal tissue were observed. Our analysis of CD4^+^ T-cell subtypes revealed a significant alteration in the Th17/Treg balance within the jejunum following BCoV infection, characterized by an increased frequency of Th17 cells alongside a decrease in Treg populations. Th17 cells, which are induced by TGF-β, IL-1β and IL-6, are known for their role in promoting mucosal inflammation through the secretion of proinflammatory cytokines such as IL-17A and IL-22, which facilitate neutrophil recruitment and enhance inflammatory responses in the epithelium^46,57–59^. In contrast, Treg cells, which are induced predominantly by TGF-β and characterized by Foxp3 expression, are functionally supported by cytokines such as IL-10 and participate in local immune tolerance associated with the intestinal microbiota^60–63^. Treg cells play a critical role in maintaining intestinal immune homeostasis and suppressing excessive immune activation^62,64^. Thus, the observed expansion of Th17 cells coupled with the reduction in Treg cells indicated a disruption of immunological equilibrium in the jejunal microenvironment. This imbalance might contribute to impaired epithelial barrier integrity and intensify local inflammatory pathology, which aligns with evidence that the jejunum is particularly vulnerable to immune-mediated damage under conditions of viral infection and metabolic stress^65,66^. Interestingly, after BCoV infection, the proportion of plasma cells increased. This is likely attributable to an enhanced mucosal humoral immune response induced by persistent exposure to viral antigens^67,68^. Plasma cells located in the intestinal lamina propria predominantly secrete IgA, which plays a critical role in neutralizing enteroviruses and inhibiting their attachment to epithelial cells^67,69^. Similarly, our research revealed that the intestinal immune network for IgA production was enriched in plasma cells after BCoV infection.

Through mediation effect analysis, we statistically validated the causal pathway through which the jejunal intestinal microbiota regulates host cellular responses via their metabolic products. This represented a key distinction between our study and conventional descriptive approaches. The results revealed that the potential promoting effect of *Phocaeicola coprophilus* on Treg cell differentiation was predominantly mediated by its metabolite DCA. Concurrently, the analysis of *Phocaeicola coprophilus* showed that the *Phocaeicola coprophilus* gene encoding BSH is involved in unconjugated bile acid biosynthesis to further affect the content of DCA^38,70^.

Notably, despite different initial insults of different pathogens causing diarrhea, they might also share common host–microbiota response features. Our research is based on microbial‒host interactions during BCoV infection; beneficial microbes, such as *Phocaeicola coprophilus*, may participate in immune regulation through secondary bile acids, and harmful microbes may promote the generation of inflammatory mediators during infections by other pathogens, suggesting that restoring these metabolic niches could be a broad-spectrum therapeutic strategy.

However, there were still several limitations in this research. First, we focused on the most severely affected jejunum, which could reflect the core microbial characteristics and host responses to BCoV infection. However, the synergistic role of the duodenum and ileum in immune activation might have been overlooked. Future research should adopt multipoint sampling to comprehensively analyze the role of the entire intestinal tract in viral defense process. Second, the sample size of this research was modest, which was constrained by the high cost and ethical considerations of large animal models. Nevertheless, the strict enrollment criteria and consistent microbiota‒host response patterns across different individuals supported the biological relevance of our findings. Although our power analysis (83.24%) suggested sufficient statistical validity, the large-scale cohorts’ studies across diverse geographic regions are still required to validate the potential effectiveness of the probiotic *Phocaeicola coprophilus* against BCoV. Finally, while our multiomics analysis confirmed that *Phocaeicola coprophilus* possessed the metabolic machinery for DCA production to affect Treg cells differentiation, the absence of direct mechanistic validation hinted the observed relationships remained associative rather than definitive. Future research utilizing in vitro metabolic assays and T-cell co-culture experiments will be essential to provide causal evidence for the immunomodulatory roles of these specific microbial candidates identified in the jejunum.

In conclusion, this study provides a multiomics landscape of the jejunal mucosal response to BCoV infection. In addition to the direct cytopathic effects on the epithelium, we demonstrated that BCoV infection induced a systemic metabolic crisis and disrupted the intestinal barrier. Importantly, our integrated analysis revealed a specific immune response related to changes in the metabolism of the intestinal microbiota. Specifically, the reduction in *Phocaeicola coprophilus* and DCA levels was potentially linked to the impairment of Treg maintenance, suggesting a potential microbial-metabolic axis in the jejunal mucosa. Consequently, this study not only established *Phocaeicola coprophilus* and DCA as potent biomarkers for distinguishing infection status but also highlights the restoration of secondary bile acid metabolism as a promising therapeutic strategy to rescue immune tolerance and mitigate intestinal injury in patients with viral enteritis.

## Methods

### Animal experiments and sample collection

A total of twelve Holstein female calves were selected from the same dairy farm and raised under identical feeding and management conditions. All calves were 10 ± 1 days of age at the time of sampling. On the basis of their clinical presentation, the calves were assigned to two groups. The BCoV-infected group (BCoV, *n* = 6) consisted of calves exhibiting acute and severe watery diarrhea, with diarrheal symptoms persisting for more than 24 h. The healthy control group (CON, *n* = 6) had a normal clinical appearance and no history of diarrhea. All calves were separated from their mothers immediately after birth and were fed high-quality first milk (IgG > 50 mg/mL) within 2 h postpartum. The calves were subsequently housed individually in calf hutches with free access to drinking water. Each calf was fed 2 L of sterilized whole milk twice daily at 05:30 and 17:30.

Fecal swab samples were collected from all candidate calves via sterile swabs, placed into sterile tubes, and stored at −80°C until analysis. Nucleic acid extraction and pathogen detection were performed according to the manufacturer’s instructions via a commercial Anieasy Calf Diarrhea Five-Pathogen Nucleic Acid Detection Kit in combination with a BVDV nucleic acid detection kit. Six major enteric pathogens, including bovine rotavirus (BRV), bovine coronavirus (BCoV), enterotoxigenic *Escherichia coli* K99 (ETEC K99), *Cryptosporidium* spp., *Clostridium perfringens*, and bovine viral diarrhea virus (BVDV), were screened. Calves were classified as BCoV-infected if they exhibited clinical diarrhea and tested positive exclusively for BCoV but tested negative for the other five pathogens. Healthy control calves were required to show no clinical symptoms and to test negative for all six pathogens.

Before euthanasia, blood samples were collected from the jugular vein. Each calf was stunned using an electric shock (KEST-2 Electrical Stimulator, Kentmaster Co., Ltd., China) with a voltage lower than 48 V for 7 to 30 s, ensuring that the calf was in an unconscious state while maintaining its heart beating. The loss of corneal and palpebral reflexes was monitored to confirm the effectiveness of the stunning. While in a recumbent position, a transverse incision was made across the neck to sever the esophagus, trachea, and major blood vessels. Exsanguination was performed for at least 6 min, followed immediately by complete necropsy. The interval between stunning and slaughter was strictly maintained under 1.5 min to prevent potential recovery of consciousness. All procedures were performed in accordance with the national standard operating procedure of livestock and poultry slaughtering cattle (GB/T 19477-2018). The entire gastrointestinal tract was rapidly removed, and tissues from different intestinal segments were collected. The jejunum was identified and processed on a sterile, chilled surface. Jejunal tissue was collected, rinsed with precooled phosphate-buffered saline (PBS), and separately preserved in tissue stabilization solution, cryogenic tubes, or specialized containers for metagenomic analysis. All sample collection procedures were completed within 30 min after euthanasia. Jejunal mucosal samples and luminal contents collected postmortem were reexamined via the same multiplex PCR assays to confirm the presence of BCoV and to exclude potential coinfections.

### Preparation of intestinal sections and H&E staining

The tissue samples were removed from the formaldehyde fixative, trimmed appropriately, and then transferred into an embedding cassette. The tissues were rinsed thoroughly with running water overnight. The samples were subsequently dehydrated through a graded ethanol series: they were sequentially immersed in 70%, 80%, 85%, and 95% ethanol for 2 h each, followed by two changes of anhydrous ethanol (I and II) for 1 h per change. The tissues were cleared in xylene for 30 min to achieve transparency. The samples were embedded in paraffin wax through three sequential baths: paraffin (I) for 30 min, paraffin (II) for 60 min, and paraffin (III) for 90 min, ensuring complete infiltration. After sectioning, the slides were placed in xylene for 7 min to remove excess paraffin and then rehydrated by washing three times with ddH₂O for 5 min each. The sections were differentiated in differentiation solution for 15 s, followed by three 5-min washes with ddH₂O. The sections were stained with eosin for 1 min and then rinsed again with ddH₂O three times for 5 min each. The stained sections were dehydrated through a graded ethanol series (75%, 85%, 95%, and anhydrous ethanol) for 5 min each. The slides were subsequently washed with xylene I and II for 10 min each. Finally, a drop of neutral balsam was mounted onto each tissue section on the slide, which was carefully covered with a coverslip, and the slides were allowed to dry at room temperature for 24 h prior to slide scanning and data collection. Case scoring was conducted for each intestinal segment section according to previous standards^71^.

### Blood chemical tests and routine blood analysis

The collected blood samples were analyzed via an automated hematology analyzer. The primary parameters measured included white blood cell count, neutrophil count, lymphocyte count, monocyte count, eosinophil count, basophil count, and neutrophil percentage. Concurrently, the serum components of the blood samples were assessed via an automated biochemical analyser. The main analytes included pyruvic acid, prealbumin, alanine aminotransferase, aspartate aminotransferase, the transaminase ratio, total bilirubin, and other markers, for a total of 24 biochemical indicators.

### Metagenomic analysis

The jejunal mucosa was collected postslaughter, and the intestinal segment was collected under sterile conditions. The mucosal surface was gently scraped using a sterile glass slide to collect epithelial tissue and adherent microbiota, which were then rinsed with PBS. The samples were frozen and stored at −80 °C until analysis. Total microbial DNA was extracted via an E.Z.N.A.® soil DNA kit (Omega Biotek, Norcross, GA, U.S.). Following the completion of DNA extraction, the concentration, purity, and integrity of the DNA were assessed. DNA fragments approximately 350 bp in size were selected for paired-end library construction. The DNA libraries were subsequently sequenced on the Illumina NovaSeq™ 6000 platform to generate paired-end reads.

The raw reads were subjected to quality control, and the host-derived sequences were removed by mapping against the reference genome (ARS-UCD2.0) via Bowtie2^72^. High-quality, host-filtered reads were then de novo assembled via MEGAHIT (v1.2.9)^73^. Contigs shorter than 500 bp were discarded to reduce assembly fragmentation while retaining low-abundance microbial genetic information in the jejunal mucosa. Assembly quality was evaluated via QUAST (v3.2)^74^. Open reading frames (ORFs) were predicted from the assembled contigs via Prodigal, and reads shorter than 100 bp were excluded, while reads longer than 100 bp were translated into amino acid sequences. The resulting protein sequences were clustered via CD-HIT at 90% identity and 90% coverage to generate a nonredundant gene catalog^75^. SOAPaligner was used to align the high-quality reads of each sample with the nonredundant gene catalog and to calculate the abundance of genes in the corresponding samples. Diamond was used to align the amino acid sequences of the nonredundant gene catalog to the NR database for taxonomic annotation and abundance calculation^76^. Concurrently, sequence comparisons were performed against the Kyoto Encyclopedia of Genes and Genomes (KEGG) and GO databases to assign KEGG functional annotations and Gene Ontology terms to the genes. Principal coordinate analysis (PCoA) based on distance matrices was performed to visualize the species composition, and analysis of similarities (ANOSIM) was used to assess significant differences in community composition between groups. Microbial features that differed between BCoV and CON were identified via linear discriminant analysis effect size (LEfSe). Linear discriminant analysis (LDA) was then used to estimate the effect size of each differentially abundant feature. Only features with a logarithmic LDA score greater than 2 and a *P* value < 0.05 were considered significantly discriminative.

### Untargeted metabolomic analysis

Metabolites were extracted from samples via the organic solvent precipitation method, with multiple quality control (QC) samples prepared in parallel to ensure analytical reproducibility. The extracted metabolites were first separated on a chromatographic column, followed by detection via a Q Exactive high-resolution tandem mass spectrometer (HR‒MS/MS). The raw mass spectrometry data were preprocessed with XCMS software, including peak detection, peak alignment, retention time correction, secondary peak grouping, and annotation of isotopic peaks and adduct ions. Subsequently, downstream data analysis was performed via R-based bioinformatics toolkits (XCMS, CAMERA, and metaX) to refine metabolite identification and quantification^77^. Metabolite annotation was achieved by matching accurate mass‒charge ratio (*m*/*z*) values against the KEGG and Human Metabolome Database (HMDB) repositories. Partial least squares-discriminant analysis (PLS-DA) was implemented via the R package ropls, and variable importance in projection (VIP) scores were calculated to assess the discriminatory contribution of each metabolite variable.

### Single-cell transcriptomic analysis

Fresh jejunal mucosal tissue was rinsed with PBS and enzymatically digested with collagenase IV at 37 °C for 30 min. The cell suspension was filtered through a 40 μm cell strainer. Quality control and cell counting of the single-cell suspensions were performed to ensure a cell survival rate greater than 80%. The qualified cells were washed, resuspended, and prepared as a single-cell suspension at a concentration of 1000–1200 cells/μL. The single-cell suspensions were subsequently loaded onto the 10x Genomics Chromium™ system. Gel bead-in-emulsions (GEMs), including beads with unique molecular identifiers (UMIs) and cell barcodes, were generated and isolated. Reverse transcription (RT) was performed on a thermal cycler for cell barcoding, followed by purification of first-strand cDNA via magnetic beads, subsequent amplification, and QC validation. Qualified cDNA was used for next-generation sequencing (NGS) library construction and quantification. Libraries were sequenced on the Illumina NovaSeq X Plus platform in paired-end 150 bp (PE150) mode. The raw sequencing data were subjected to QC and statistical analysis via FastQC. Read quality was evaluated, and sequence alignment against the *Bos taurus* reference genome (ARS-UCD2.0) was performed via Cell Ranger (Cell Ranger | Official 10x Genomics Support). Seurat software was employed for cell filtering and multisample integration following standard workflows^78^. After filtering, data normalization was implemented via the log normalization method^78^. Uniform manifold approximation and projection (UMAP) was used for dimensionality reduction of single-cell sequencing data, and the shared nearest neighbor algorithm was applied for cell clustering^79^. Marker genes were identified on the basis of previously published single-cell sequencing datasets of dairy cows^80^.

### Mediation analysis

Mediation analysis was performed to evaluate whether key metabolites mediate the association between microbiota abundance and the proportion of host cells in the jejunal mucosa. All analyses were conducted in R (version 4.3.2). Microbial abundance was treated as the independent variable (X), metabolite concentration as the mediator (M), and cell proportion as the outcome variable (Y). A linear regression model was fitted to assess the associations between the microbiota and metabolites (M~X). A regression model was subsequently fitted to examine associations between metabolites and cell proportions (Y~M). A third model evaluated the total effect of the microbiota on the cell proportion (Y~X). A causal mediation model incorporating both the independent variable and the mediator (Y~X + M) was subsequently constructed. The mediation effect was estimated via the mediate function from the R package: mediation, with nonparametric bootstrapping (1000 simulations), to obtain confidence intervals for the average causal mediation effect (ACME), average direct effect (ADE), and total effect. Model fit was evaluated via the coefficient of determination (*R*²) for each regression model. A two-sided *P* value < 0.05 was considered to indicate statistical significance.

### Statistical analysis

This study performed a post hoc statistical power analysis using the limma-empirical Bayes framework. Assuming a conventional power threshold of 80%, the analysis indicated that the current sample size (*n* = 6 per group) achieved an estimated statistical power of 83.24%. KEGG pathway enrichment analysis for DEGs was conducted via KOBAS software. All other statistical analyses were performed with IBM SPSS Statistics 27, where the Mann‒Whitney U test was applied for intergroup comparisons. Statistical significance was defined as *P* < 0.05, *P* < 0.01, and *P* < 0.001, and all graphical visualizations were generated via GraphPad Prism 9.5. The genome of *Phocaeicola coprophilus* was downloaded from the NCBI RefSeq assembly GCF_016888945.1 (https://www.ncbi.nlm.nih.gov/datasets/genome/GCF_016888945.1/). The protein sequence was aligned with the KEGG database by GhostKOALA (https://www.kegg.jp/ghostkoala/). Correlation analysis was carried out in RStudio via R packages, including BiocManager and ComplexHeatmap, with Spearman’s rank correlation coefficients calculated to evaluate associations between variables. Receiver operating characteristic (ROC) curve analysis was also performed in GraphPad Prism 9.5 via the Wilson–Brown method, with 95% confidence intervals (CIs) computed.

### Ethics approval and consent to participate

This experiment was conducted in accordance with the recommended guidelines from the Administration of Affairs Concerning Experimental Animals (Ministry of Science and Technology, China, revised 2004). The animal protocol was approved by the Institutional Animal Care and Use Committee of Northwest A&F University (ethical approval number: NWAFU-DY20240419).

## Supplementary information

Supplementary information

## Data Availability

The raw sequencing data from the metagenome, single-cell RNA sequencing and metabolome were deposited into the China National Center for Bioinformation (CNCB; https://www.cncb.ac.cn/?lang=en) under accession number PRJCA051918.

## Code availability

There were no custom codes, and all analyses relied on publicly available software with the specified parameters.
