# Supplementary material for: Multi-omics atlas of the bovine coronavirus-infected calf jejunum: reduction of Phocaeicola coprophilus and deoxycholic acid linked to Th17/Treg imbalance
Source: NPJ Biofilms Microbiomes. 2026 May 7;12:136. doi: 10.1038/s41522-026-00997-7 (PMC13365524; doi:10.1038/s41522-026-00997-7)
Supplement: Supplementary file 1 — Supplementary information [file 41522_2026_997_MOESM1_ESM.pdf]

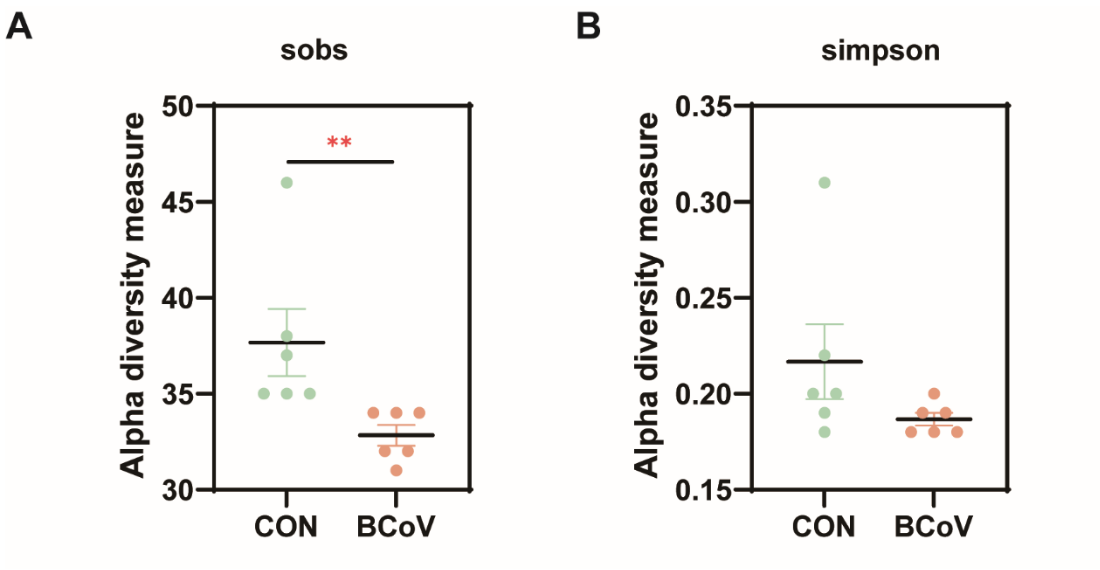

Supplementary Fig. 1. The α diversity of microbiota in jejunal mucosa. **(A)** The sobs index differences between BCoV and CON groups. **(B)** The Simpson index differences between BCoV and CON groups.

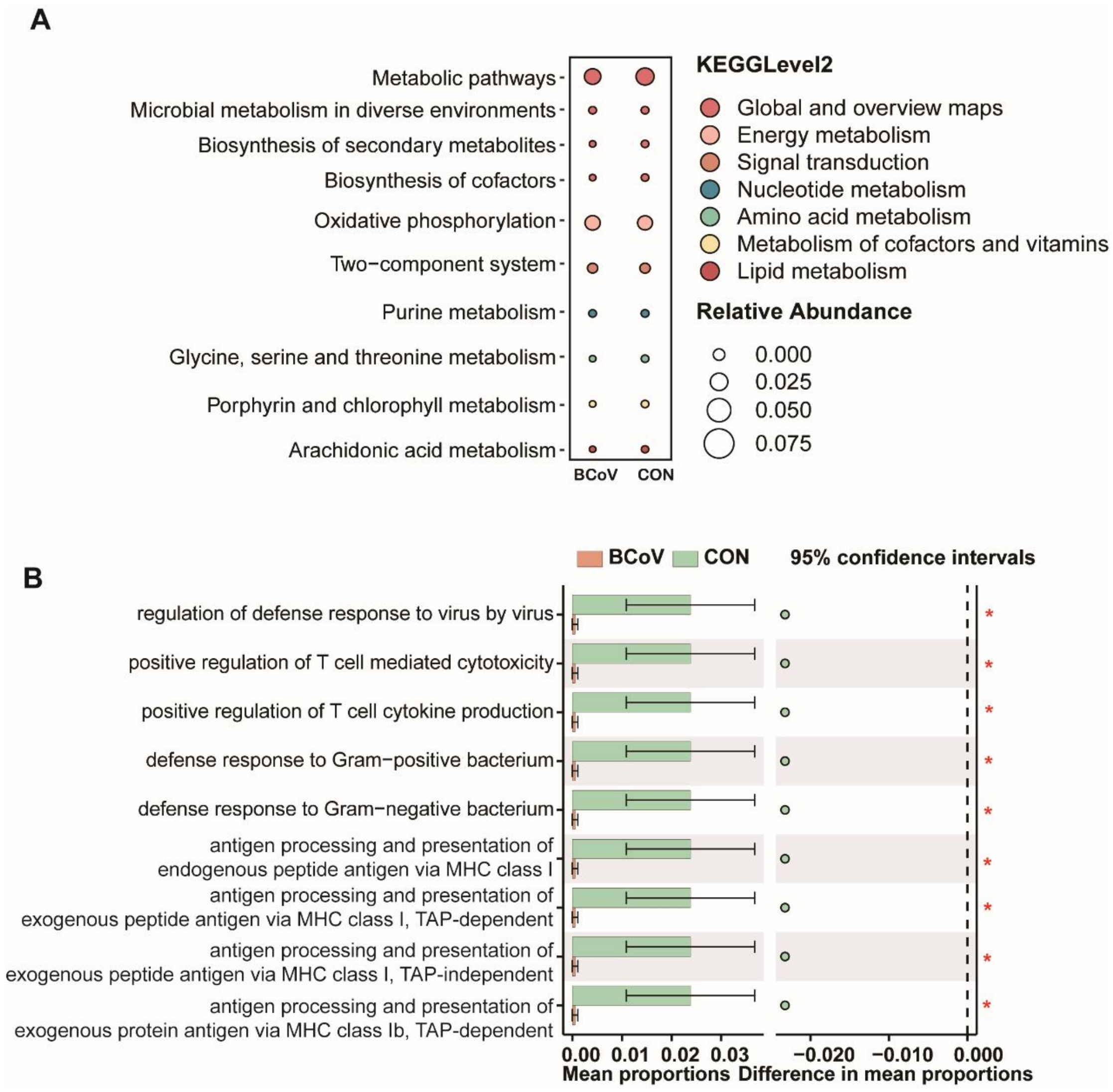

Supplementary Fig. 2. The microbial functional differences between BCoV and CON groups. **(A)** The KEGG enrichment analysis differences. **(B)** The GO enrichment analysis differences.

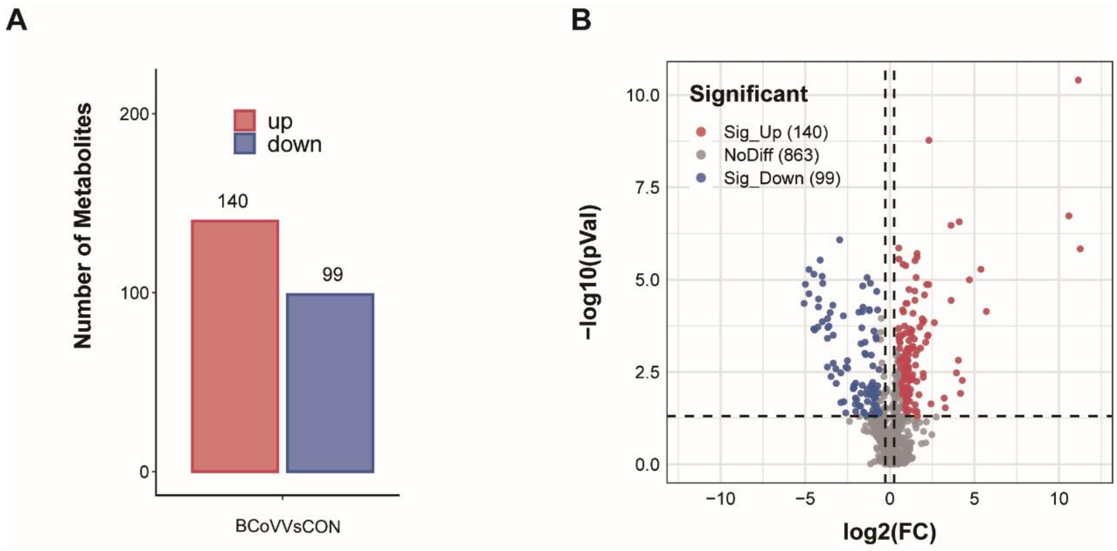

Supplementary Fig. 3. The metabolites differences in jejunal mucosa between BCoV and CON. **(A)** The bar chart shows the number of differential metabolites. **(B)** The volcano plot shows the number of differential metabolites.

A

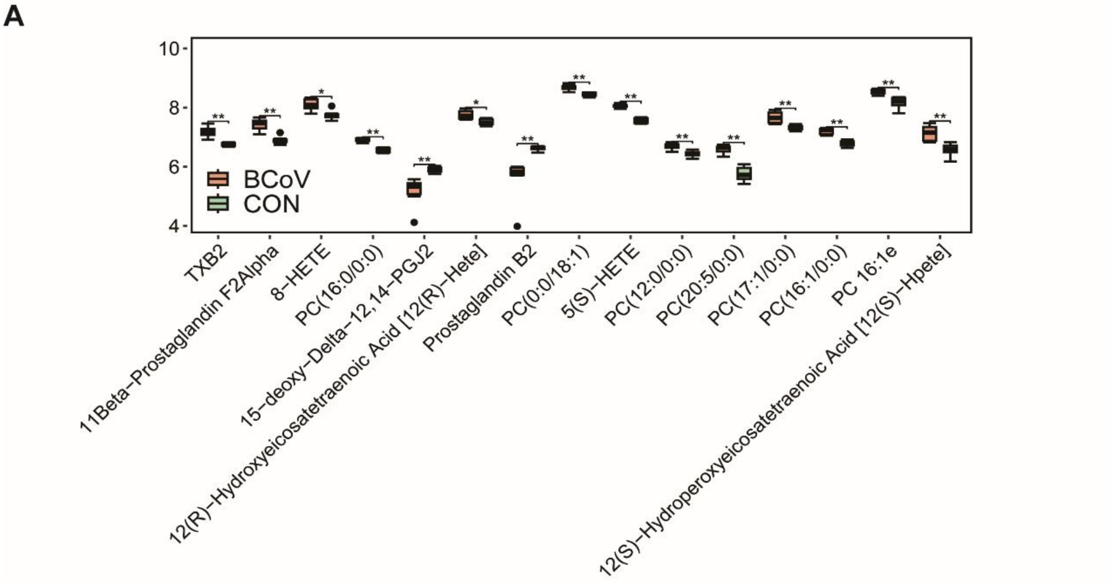

B

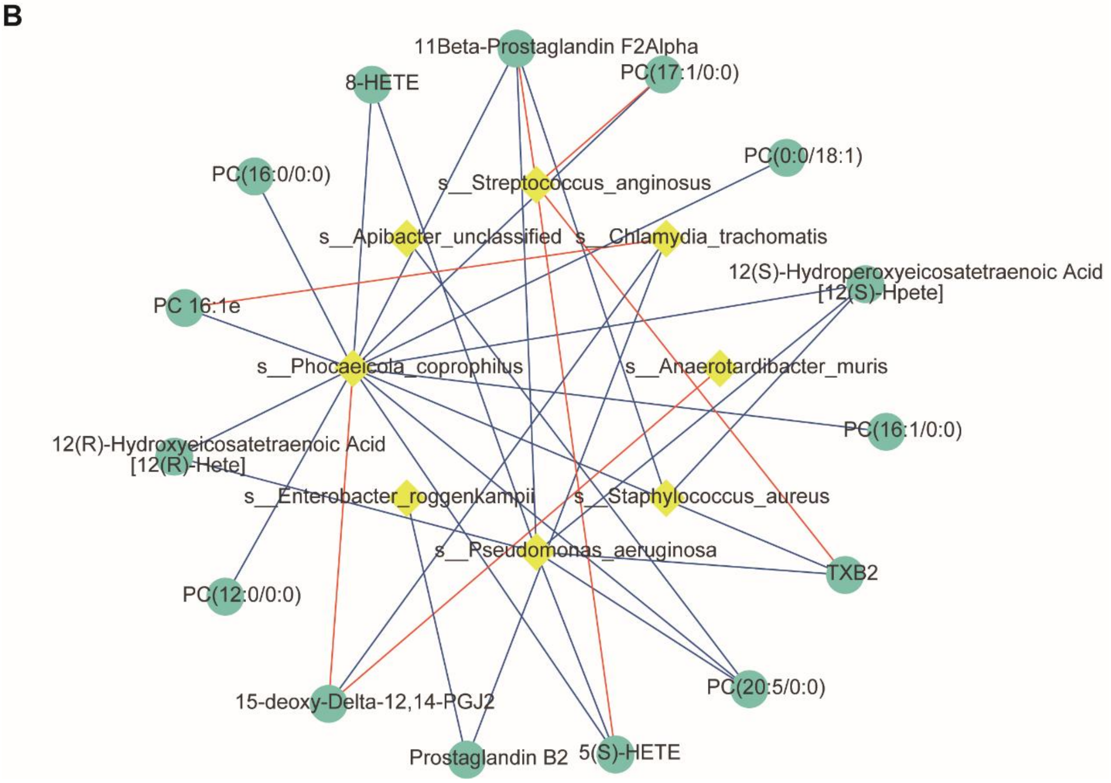

Supplementary Fig. 4. The arachidonic metabolism derived metabolites differences and the correction analysis between these metabolites with microbiota. **(A)** The box plot shows the differences in arachidonic acid metabolites between BCoV and CON. **(B)** Association analysis of arachidonic acid metabolites with microbiota.

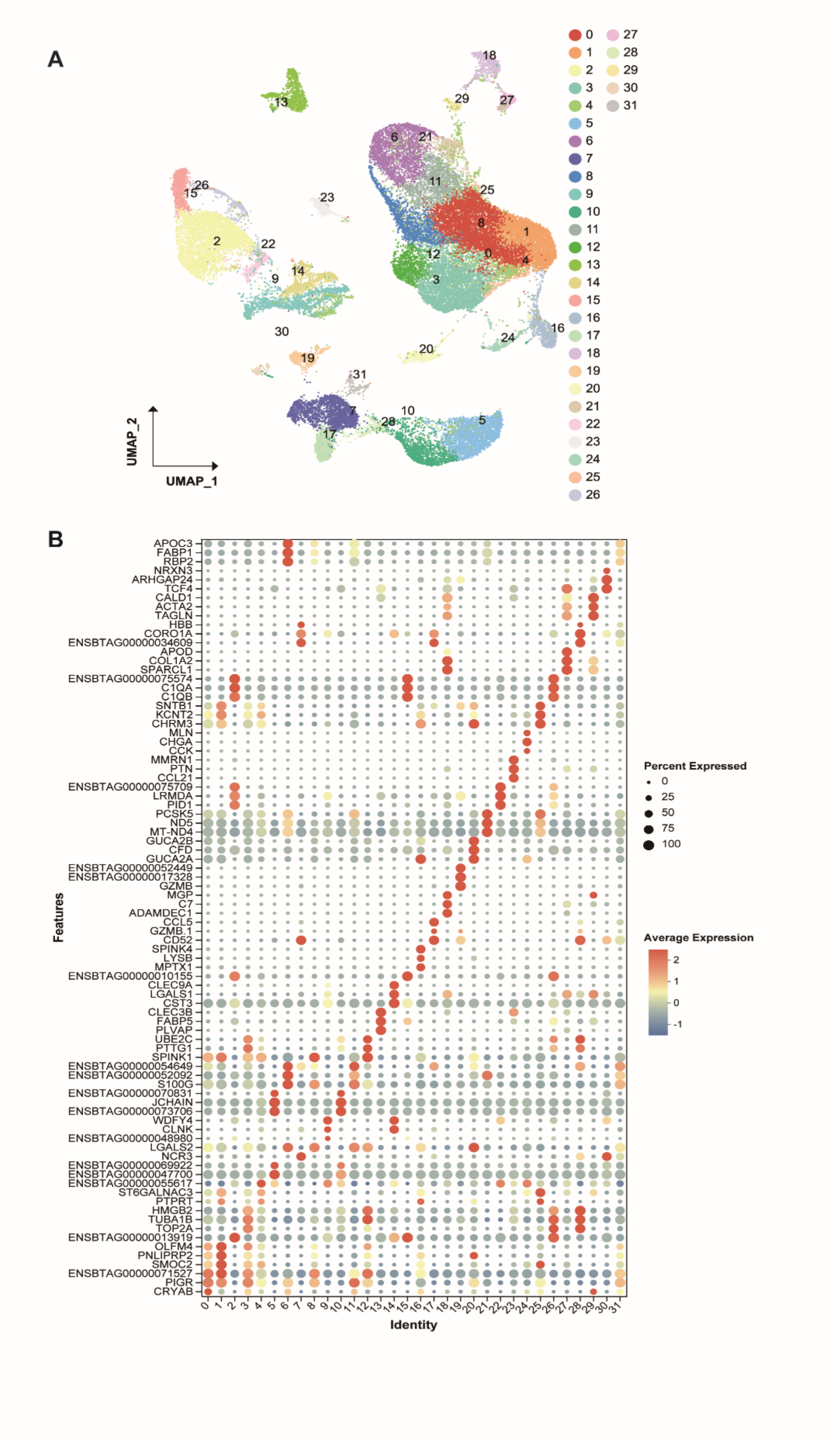

Supplementary Fig. 5. Single-cell clustering under UMAP dimensionality reduction and the markers of each cluster. **(A)** Single cell clustering under UMAP dimensionality reduction. **(B)** The top 3 marker genes of each cluster.

Supplementary Table 1 The microbial functional analysis of GO term with significant differences between BCoV and CON groups

description	mean_BCoV	mean_CON	se_BCoV	se_CON	p_value	sign
1-phosphatidylinositol binding	0.818920599	0.716814575	0.037378845	0.016651503	0.037372988	*
antigen processing and presentation of endogenous peptide antigen via MHC class I	0.000560867	0.02381487	0.000560867	0.012933481	0.049510167	*
antigen processing and presentation of exogenous peptide antigen via MHC class I, TAP-dependent	0.000560867	0.02381487	0.000560867	0.012933481	0.049510167	*
antigen processing and presentation of exogenous peptide antigen via MHC class I, TAP-independent	0.000560867	0.02381487	0.000560867	0.012933481	0.049510167	*
antigen processing and presentation of exogenous protein antigen via MHC class Ib, TAP-dependent	0.000560867	0.02381487	0.000560867	0.012933481	0.049510167	*
bitter taste receptor activity	0.361286071	0.285536457	0.022635866	0.012726556	0.024974679	*
cellular protein metabolic process	0.000560867	0.02381487	0.000560867	0.012933481	0.049510167	*
cellular response to iron ion	0.000560867	0.02381487	0.000560867	0.012933481	0.049510167	*
cellular response to lipopolysaccharide	0.000560867	0.02381487	0.000560867	0.012933481	0.049510167	*
chemical synaptic transmission	0.818920599	0.716814575	0.037378845	0.016651503	0.037372988	*
clathrin binding	0.818920599	0.716814575	0.037378845	0.016651503	0.037372988	*
clathrin coat assembly	0.818920599	0.716814575	0.037378845	0.016651503	0.037372988	*
clathrin-coated pit	0.818920599	0.716814575	0.037378845	0.016651503	0.037372988	*
clathrin-coated vesicle	0.818920599	0.716814575	0.037378845	0.016651503	0.037372988	*
defense response to Gram-negative bacterium	0.000560867	0.02381487	0.000560867	0.012933481	0.049510167	*
defense response to Gram-positive bacterium	0.000560867	0.02381487	0.000560867	0.012933481	0.049510167	*
detection of chemical stimulus involved in sensory perception of bitter taste	0.361286071	0.285536457	0.022635866	0.012726556	0.024974679	*

detection of chemical stimulus involved in sensory perception of smell	0.012484399	0.027457041	0.003090899	0.003920491	0.024974679	*
DNA replication	0	0.017060251	0	0.012250996	0.007397125	**
early endosome lumen	0.000560867	0.02381487	0.000560867	0.012933481	0.049510167	*
early endosome membrane	0.000560867	0.02381487	0.000560867	0.012933481	0.049510167	*
endoplasmic reticulum lumen	0.000560867	0.02381487	0.000560867	0.012933481	0.049510167	*
ER to Golgi transport vesicle membrane	0.000560867	0.02381487	0.000560867	0.012933481	0.049510167	*
external side of plasma membrane	0.000560867	0.02381487	0.000560867	0.012933481	0.049510167	*
G-protein coupled receptor activity	0.012484399	0.027457041	0.003090899	0.003920491	0.024974679	*
Golgi apparatus	0.000560867	0.02381487	0.000560867	0.012933481	0.049510167	*
Golgi membrane	0.000560867	0.02381487	0.000560867	0.012933481	0.049510167	*
HFE-transferrin receptor complex	0.000560867	0.02381487	0.000560867	0.012933481	0.049510167	*
identical protein binding	0.007936046	0.045207721	0.000909523	0.017513347	0.037372988	*
interferon-gamma-mediated signaling pathway	0.000560867	0.02381487	0.000560867	0.012933481	0.049510167	*
iron ion homeostasis	0.000560867	0.02381487	0.000560867	0.012933481	0.049510167	*
mitochondrial inner membrane	0.022721886	0.050739947	0.005796792	0.013039223	0.024974679	*
negative regulation of neuron projection development	0.000560867	0.02381487	0.000560867	0.012933481	0.049510167	*
negative regulation of receptor binding	0.000560867	0.02381487	0.000560867	0.012933481	0.049510167	*
neutrophil degranulation	0.00116721	0.029247814	0.00116721	0.01569586	0.021025213	*
olfactory receptor activity	0.012484399	0.038924307	0.003090899	0.011175973	0.016309172	*
phagocytic vesicle membrane	0.000560867	0.02381487	0.000560867	0.012933481	0.049510167	*
positive regulation of ferrous iron binding	0.000560867	0.02381487	0.000560867	0.012933481	0.049510167	*
positive regulation of protein binding	0.000560867	0.02381487	0.000560867	0.012933481	0.049510167	*
positive regulation of receptor binding	0.000560867	0.02381487	0.000560867	0.012933481	0.049510167	*
positive regulation of receptor-mediated endocytosis	0.000560867	0.02381487	0.000560867	0.012933481	0.049510167	*
positive regulation of T cell cytokine production	0.000560867	0.02381487	0.000560867	0.012933481	0.049510167	*

positive regulation of T cell mediated cytotoxicity	0.000560867	0.02381487	0.000560867	0.012933481	0.049510167	*
positive regulation of transferrin receptor binding	0.000560867	0.02381487	0.000560867	0.012933481	0.049510167	*
protein refolding	0.000560867	0.02381487	0.000560867	0.012933481	0.049510167	*
Ran GTPase binding	0.035202753	0.05909858	0.006457854	0.00762347	0.037372988	*
recycling endosome membrane	0.000560867	0.02381487	0.000560867	0.012933481	0.049510167	*
regulation of clathrin-dependent endocytosis	0.818920599	0.716814575	0.037378845	0.016651503	0.037372988	*
regulation of defense response to virus by virus	0.000560867	0.02381487	0.000560867	0.012933481	0.049510167	*
regulation of membrane depolarization	0.000560867	0.02381487	0.000560867	0.012933481	0.049510167	*
response to cadmium ion	0.000560867	0.02381487	0.000560867	0.012933481	0.049510167	*
response to drug	0.001714901	0.03534223	0.000767087	0.022907917	0.027768363	*
sequence-specific DNA binding	0	0.005241525	0	0.001708917	0.007397125	**
specific granule lumen	0.000560867	0.02381487	0.000560867	0.012933481	0.049510167	*
T cell differentiation in thymus	0.000560867	0.02381487	0.000560867	0.012933481	0.049510167	*
tertiary granule lumen	0.000560867	0.02381487	0.000560867	0.012933481	0.049510167	*

Supplementary Table 2. **The correlation analysis of microbiome and metabolites in arachidonic metabolism**

Data1	Data2	rho	pvalue	relation
s__Phocaeicola_coprophilus	PC(16:0/0:0)	-0.896769649	7.75E-05	negtive
s__Phocaeicola_coprophilus	PC(16:1/0:0)	-0.888971653	0.00010998	negtive
s__Phocaeicola_coprophilus	PC(0:0/18:1)	-0.842183671	0.00058779	negtive
s__Phocaeicola_coprophilus	PC(17:1/0:0)	-0.826587677	0.000915842	negtive
s__Phocaeicola_coprophilus	PC 16:1e	-0.81878968	0.001125347	negtive
s__Phocaeicola_coprophilus	PC(12:0/0:0)	-0.803193686	0.001653531	negtive
s__Phocaeicola_coprophilus	5(S)-HETE	-0.795395689	0.00198009	negtive
s__Pseudomonas_aeruginosa	12(S)-Hydroperoxyeicosatetraenoic Acid [12(S)-Hpete]	-0.783122887	0.002591379	negtive
s__Chlamydia_trachomatis	PC 16:1e	0.768620611	0.003488367	positive
s__Phocaeicola_coprophilus	11Beta-Prostaglandin F2Alpha	-0.764203701	0.003803461	negtive
s__Pseudomonas_aeruginosa	12(R)-Hydroxyeicosatetraenoic Acid [12(R)-Hete]	-0.739616059	0.005969519	negtive
s__Phocaeicola_coprophilus	15-deoxy-Delta-12,14-PGJ2	0.733011713	0.006683813	positive
s__Phocaeicola_coprophilus	12(S)-Hydroperoxyeicosatetraenoic Acid [12(S)-Hpete]	-0.733011713	0.006683813	negtive
s__Pseudomonas_aeruginosa	TXB2	-0.717862646	0.008563333	negtive
s__Phocaeicola_coprophilus	TXB2	-0.701819726	0.010957379	negtive
s__Pseudomonas_aeruginosa	11Beta-Prostaglandin F2Alpha	-0.696109232	0.011919049	negtive
s__Streptococcus_anginosus	PC(17:1/0:0)	0.692307692	0.015877693	positive
s__Pseudomonas_aeruginosa	8-HETE	-0.681606957	0.014641746	negtive
s__Chlamydia_trachomatis	15-deoxy-Delta-12,14-PGJ2	-0.674355819	0.016163124	negtive
s__Phocaeicola_coprophilus	8-HETE	-0.647233747	0.022896405	negtive
s__Pseudomonas_aeruginosa	5(S)-HETE	-0.63810013	0.02556511	negtive

s__Pseudomonas_aeruginosa	PC(20:5/0:0)	-0.630848992	0.027838786	negtive
s__Staphylococcus_aureus	11Beta-Prostaglandin F2Alpha	-0.629370629	0.03239475	negtive
s__Streptococcus_anginosus	TXB2	0.629370629	0.03239475	positive
s__Phocaeicola_coprophilus	12(R)-Hydroxyeicosatetraenoic Acid [12(R)-Hete]	-0.623839756	0.030171995	negtive
s__Apibacter_unclassified	PC(20:5/0:0)	-0.623468557	0.030299355	negtive
s__Staphylococcus_aureus	12(S)-Hydroperoxyeicosatetraenoic Acid [12(S)-Hpete]	-0.622377622	0.034800123	negtive
s__Enterobacter_roggenkampii	Prostaglandin B2	-0.615384615	0.037334511	negtive
s__Streptococcus_anginosus	11Beta-Prostaglandin F2Alpha	0.615384615	0.037334511	positive
s__Chlamydia_trachomatis	Prostaglandin B2	-0.609095578	0.035534073	negtive
s__Phocaeicola_coprophilus	PC(20:5/0:0)	-0.608243762	0.035863294	negtive
s__Anaerotardibacter_muris	15-deoxy-Delta-12,14-PGJ2	0.605955395	0.036758516	positive
s__Streptococcus_anginosus	5(S)-HETE	0.601398601	0.042806869	positive

Supplementary Table 3. The proportion of cell types in jejunum mucosal of calves

Celltype	BCoV	CON
Blood vascular endothelial cells	0.305974248	0.261102767
CD4+ T cells	0.016962753	0.092908097
DC	0.029077541	0.017417477
Cytotoxic CD8+ T cells	0.002932694	0.033291112
Enterocytes	0.04530475	0.130789594
Fibroblasts	0.010412098	0.042208039
Goblet cells	0.020553812	0.020410546
Lymphatic endothelial cells	0.02191406	0.053754293
Macrophages	0.137111378	0.094082013
Mast cells	0.005743062	0.023703059
Myofibroblasts	0.003872123	0.007763897
Plasma cells	0.162951836	0.034913373
Progenitor cells	0.013170205	0.006437249
Proliferative T cells	0.115421442	0.092150748
unknown	0.108597997	0.089067736

Supplementary Table 4. The KEGG enrichment analysis of differential expression genes in Enterocytes between BCoV and CON groups

#Term	ID	Input number	Corrected P-Value
Oxidative phosphorylation	bta00190	76	7.48E-55
Parkinson disease	bta05012	76	1.85E-53
Huntington disease	bta05016	81	1.42E-51
Metabolic pathways	bta01100	182	1.20E-47
Thermogenesis	bta04714	82	1.20E-47
Alzheimer disease	bta05010	72	1.87E-45
Ribosome	bta03010	65	2.37E-41
Non-alcoholic fatty liver disease (NAFLD)	bta04932	59	6.98E-36
Retrograde endocannabinoid signaling	bta04723	36	2.33E-16
Carbon metabolism	bta01200	28	3.68E-13
Cardiac muscle contraction	bta04260	23	4.48E-11
Proteasome	bta03050	16	2.28E-09
Biosynthesis of amino acids	bta01230	16	5.98E-07
Citrate cycle (TCA cycle)	bta00020	11	1.11E-06
Fatty acid degradation	bta00071	12	2.50E-06
Protein processing in endoplasmic reticulum	bta04141	21	1.46E-05
Fluid shear stress and atherosclerosis	bta05418	19	3.09E-05
Cellular senescence	bta04218	20	5.03E-05
Endocytosis	bta04144	25	5.03E-05
Peroxisome	bta04146	14	5.12E-05
Glycolysis / Gluconeogenesis	bta00010	12	8.10E-05
Kaposi sarcoma-associated herpesvirus infection	bta05167	22	8.10E-05
Epstein-Barr virus infection	bta05169	23	0.000113222
Tuberculosis	bta05152	21	0.000132014
Arginine and proline metabolism	bta00330	10	0.000188428
Dopaminergic synapse	bta04728	16	0.000310474
Amphetamine addiction	bta05031	11	0.000471698
HIF-1 signaling pathway	bta04066	14	0.000547309
PPAR signaling pathway	bta03320	12	0.000547309
Ferroptosis	bta04216	9	0.000550063
Apoptosis	bta04210	16	0.000579516
Influenza A	bta05164	18	0.000725541
Pentose phosphate pathway	bta00030	7	0.001019969
Phagosome	bta04145	17	0.001207251
Synaptic vesicle cycle	bta04721	11	0.001207251
Estrogen signaling pathway	bta04915	15	0.001207251
Mitophagy - animal	bta04137	10	0.001375478
Vitamin digestion and absorption	bta04977	6	0.002262411

Fatty acid metabolism	bta01212	9	0.002335083
Osteoclast differentiation	bta04380	14	0.002412075
Mineral absorption	bta04978	8	0.002746321
Fat digestion and absorption	bta04975	8	0.00298398
mTOR signaling pathway	bta04150	15	0.00298398
Human papillomavirus infection	bta05165	25	0.003440733
Pertussis	bta05133	10	0.003440733
Pyruvate metabolism	bta00620	7	0.003724409
Valine, leucine and isoleucine degradation	bta00280	8	0.003820742
Pathways in cancer	bta05200	34	0.003820742
Tight junction	bta04530	16	0.003858049
Human T-cell leukemia virus 1 infection	bta05166	19	0.003899007
MAPK signaling pathway	bta04010	22	0.003899007
Collecting duct acid secretion	bta04966	6	0.004076448
Aldosterone synthesis and secretion	bta04925	11	0.004084613
NOD-like receptor signaling pathway	bta04621	16	0.004131828
Arginine biosynthesis	bta00220	5	0.004907082
Relaxin signaling pathway	bta04926	13	0.00491247
Rheumatoid arthritis	bta05323	11	0.005980297
Glucagon signaling pathway	bta04922	11	0.006785059
Colorectal cancer	bta05210	10	0.00721444
C-type lectin receptor signaling pathway	bta04625	11	0.00743138
Endocrine and other factor-regulated calcium reabsorption	bta04961	7	0.00743138
Proximal tubule bicarbonate reclamation	bta04964	5	0.00743138
Necroptosis	bta04217	15	0.00743138
Human immunodeficiency virus 1 infection	bta05170	18	0.00743138
Fructose and mannose metabolism	bta00051	6	0.007889291
Glioma	bta05214	9	0.008568793
GnRH signaling pathway	bta04912	10	0.008568793
Hepatitis C	bta05160	14	0.00923494
Cholesterol metabolism	bta04979	7	0.010333398
cGMP-PKG signaling pathway	bta04022	14	0.011573905
Oxytocin signaling pathway	bta04921	13	0.012350956
Purine metabolism	bta00230	12	0.012995522
Hepatocellular carcinoma	bta05225	14	0.014095482
Salmonella infection	bta05132	9	0.014095482
Melanogenesis	bta04916	10	0.014162021
Rap1 signaling pathway	bta04015	16	0.015590091
Apelin signaling pathway	bta04371	12	0.015932628
Legionellosis	bta05134	7	0.016181779
Renin secretion	bta04924	8	0.016686608
Fatty acid elongation	bta00062	5	0.016686608
RNA polymerase	bta03020	5	0.016686608

2-Oxocarboxylic acid metabolism	bta01210	4	0.018278648
Bacterial invasion of epithelial cells	bta05100	8	0.018278648
Glyoxylate and dicarboxylate metabolism	bta00630	5	0.018278648
Leishmaniasis	bta05140	8	0.020838559
cAMP signaling pathway	bta04024	16	0.02220739
FoxO signaling pathway	bta04068	11	0.024343223
Propanoate metabolism	bta00640	5	0.024907981
Measles	bta05162	12	0.025007567
Hepatitis B	bta05161	13	0.025312906
Chagas disease (American trypanosomiasis)	bta05142	10	0.026571856
Circadian entrainment	bta04713	9	0.029648
beta-Alanine metabolism	bta00410	5	0.032496152
Viral carcinogenesis	bta05203	16	0.032565259
PI3K-Akt signaling pathway	bta04151	22	0.033102612
Folate biosynthesis	bta00790	5	0.034801003
Lysosome	bta04142	10	0.03508563
Long-term potentiation	bta04720	7	0.03508563
Adherens junction	bta04520	7	0.03508563
Human cytomegalovirus infection	bta05163	16	0.03508563
IL-17 signaling pathway	bta04657	8	0.045221772
Gastric acid secretion	bta04971	7	0.047188288
Adrenergic signaling in cardiomyocytes	bta04261	11	0.047188288

Supplementary Table 5. The KEGG enrichment analysis of differential expression genes in Lymphatic endothelial cells between BCoV and CON groups

#Term	ID	Input number	Corrected P-Value
Ribosome	bta03010	53	6.63E-34
Phagosome	bta04145	21	4.86E-06
Pathways in cancer	bta05200	39	6.62E-06
Rap1 signaling pathway	bta04015	23	6.62E-06
Proteoglycans in cancer	bta05205	22	7.02E-06
Oxidative phosphorylation	bta00190	18	8.92E-06
Human papillomavirus infection	bta05165	29	9.50E-06
Fluid shear stress and atherosclerosis	bta05418	18	1.07E-05
Kaposi sarcoma-associated herpesvirus infection	bta05167	21	2.03E-05
Regulation of actin cytoskeleton	bta04810	21	2.67E-05
PI3K-Akt signaling pathway	bta04151	29	2.67E-05
Influenza A	bta05164	19	2.75E-05
Focal adhesion	bta04510	20	2.75E-05
Alzheimer disease	bta05010	19	2.75E-05
Proteasome	bta03050	10	2.99E-05
Thermogenesis	bta04714	22	2.99E-05
Parkinson disease	bta05012	16	0.000134476
Apelin signaling pathway	bta04371	15	0.000230405
Huntington disease	bta05016	18	0.000252817
Ras signaling pathway	bta04014	20	0.000302009
Epstein-Barr virus infection	bta05169	19	0.000399543
Metabolic pathways	bta01100	67	0.000399543
Human T-cell leukemia virus 1 infection	bta05166	19	0.000522972
Rheumatoid arthritis	bta05323	12	0.00053383
HIF-1 signaling pathway	bta04066	12	0.000960423
MAPK signaling pathway	bta04010	21	0.000960423
Pertussis	bta05133	10	0.000961418
Relaxin signaling pathway	bta04926	13	0.001189163
Mitophagy - animal	bta04137	9	0.00142888
Non-alcoholic fatty liver disease (NAFLD)	bta04932	14	0.001724896
Endocytosis	bta04144	18	0.001957552
ECM-receptor interaction	bta04512	10	0.001957552
Complement and coagulation cascades	bta04610	10	0.001957552
Protein processing in endoplasmic reticulum	bta04141	14	0.002301121
Gap junction	bta04540	10	0.002368722
Staphylococcus aureus infection	bta05150	9	0.002739545
Oxytocin signaling pathway	bta04921	13	0.003146273
Leukocyte transendothelial migration	bta04670	11	0.003146273
Tight junction	bta04530	14	0.003982824

Glycolysis / Gluconeogenesis	bta00010	8	0.003982824
Human cytomegalovirus infection	bta05163	17	0.003982824
TNF signaling pathway	bta04668	11	0.003982824
Protein export	bta03060	5	0.00472274
AGE-RAGE signaling pathway in diabetic complications	bta04933	10	0.004993555
Salmonella infection	bta05132	9	0.005017205
Hepatitis C	bta05160	13	0.005017205
Chemokine signaling pathway	bta04062	14	0.005831901
Retrograde endocannabinoid signaling	bta04723	12	0.007767975
GnRH signaling pathway	bta04912	9	0.008270366
Leishmaniasis	bta05140	8	0.009030022
Axon guidance	bta04360	13	0.009925634
Cell adhesion molecules (CAMs)	bta04514	12	0.010221449
Cellular senescence	bta04218	12	0.013994999
Tuberculosis	bta05152	13	0.020276048
IL-17 signaling pathway	bta04657	8	0.021762364
Vascular smooth muscle contraction	bta04270	10	0.021762364
Osteoclast differentiation	bta04380	10	0.021762364
Spliceosome	bta03040	10	0.021762364
Toxoplasmosis	bta05145	9	0.023132813
Melanoma	bta05218	7	0.023298621
NOD-like receptor signaling pathway	bta04621	12	0.023298621
Thyroid hormone synthesis	bta04918	7	0.023901987
Small cell lung cancer	bta05222	8	0.023901987
Intestinal immune network for IgA production	bta04672	6	0.023901987
Chagas disease (American trypanosomiasis)	bta05142	9	0.023901987
Prostate cancer	bta05215	8	0.023901987
Viral protein interaction with cytokine and cytokine receptor	bta04061	8	0.023901987
Signaling pathways regulating pluripotency of stem cells	bta04550	10	0.024711315
Th1 and Th2 cell differentiation	bta04658	8	0.027151042
Apoptosis	bta04210	10	0.027151042
Synaptic vesicle cycle	bta04721	7	0.027151042
Sphingolipid signaling pathway	bta04071	9	0.027151042
Platelet activation	bta04611	9	0.028123287
Glutathione metabolism	bta00480	6	0.028731262
Neurotrophin signaling pathway	bta04722	9	0.028731262
Melanogenesis	bta04916	8	0.031013124
PPAR signaling pathway	bta03320	7	0.032275707
Parathyroid hormone synthesis, secretion and action	bta04928	8	0.032540035
Glucagon signaling pathway	bta04922	8	0.032540035
Collecting duct acid secretion	bta04966	4	0.032540035

Ribosome biogenesis in eukaryotes	bta03008	7	0.032540035
Hippo signaling pathway	bta04390	10	0.034499262
C-type lectin receptor signaling pathway	bta04625	8	0.034499262
Measles	bta05162	10	0.034499262
Phospholipase D signaling pathway	bta04072	10	0.034499262
Endocrine and other factor-regulated calcium reabsorption	bta04961	5	0.034499262
Central carbon metabolism in cancer	bta05230	6	0.034562584
Necroptosis	bta04217	11	0.034936936
Calcium signaling pathway	bta04020	12	0.036247395
Dopaminergic synapse	bta04728	9	0.037748078
Human immunodeficiency virus 1 infection	bta05170	13	0.042129469
Adherens junction	bta04520	6	0.042129469
Cholesterol metabolism	bta04979	5	0.043406144
Cholinergic synapse	bta04725	8	0.045021635
N-Glycan biosynthesis	bta00510	5	0.045053041
Estrogen signaling pathway	bta04915	9	0.045053041
Renal cell carcinoma	bta05211	6	0.045053041
TGF-beta signaling pathway	bta04350	7	0.045360558
Salivary secretion	bta04970	7	0.045360558
Transcriptional misregulation in cancer	bta05202	11	0.045360558
Renin secretion	bta04924	6	0.045870413
Fc gamma R-mediated phagocytosis	bta04666	7	0.046648972
Bacterial invasion of epithelial cells	bta05100	6	0.049910681
Gastric acid secretion	bta04971	6	0.049910681
Aldosterone synthesis and secretion	bta04925	7	0.049953415

Supplementary Table 6. The KEGG enrichment analysis of differential expression genes in Cytoxic CD8+T cells between BCoV and CON groups

#Term	ID	Input number	Corrected P-Value
Ribosome	bta03010	62	1.10E-94
Parkinson disease	bta05012	8	1.28E-06
Salmonella infection	bta05132	6	6.27E-06
Fc gamma R-mediated phagocytosis	bta04666	6	1.08E-05
Alzheimer disease	bta05010	7	4.26E-05
Huntington disease	bta05016	7	7.60E-05
Oxidative phosphorylation	bta00190	6	9.12E-05
Cardiac muscle contraction	bta04260	5	0.000112534
Regulation of actin cytoskeleton	bta04810	7	0.000114666
Non-alcoholic fatty liver disease (NAFLD)	bta04932	6	0.000172332
Thermogenesis	bta04714	7	0.000234171
Bacterial invasion of epithelial cells	bta05100	4	0.000619325
Human immunodeficiency virus 1 infection	bta05170	6	0.001283744
Human T-cell leukemia virus 1 infection	bta05166	5	0.006870172
Pertussis	bta05133	3	0.007399748
Endocytosis	bta04144	5	0.008392894
PD-L1 expression and PD-1 checkpoint pathway in cancer	bta05235	3	0.012153208
Th1 and Th2 cell differentiation	bta04658	3	0.013924606
T cell receptor signaling pathway	bta04660	3	0.017464563
Primary immunodeficiency	bta05340	2	0.01760341
Th17 cell differentiation	bta04659	3	0.020077896
Chagas disease (American trypanosomiasis)	bta05142	3	0.021460948
TNF signaling pathway	bta04668	3	0.022411311
Proteasome	bta03050	2	0.023677718
Mineral absorption	bta04978	2	0.024606347
Lysosome	bta04142	3	0.024886379
cAMP signaling pathway	bta04024	4	0.028552215
Yersinia infection	bta05135	3	0.029140651
Human cytomegalovirus infection	bta05163	4	0.035627426
Fluid shear stress and atherosclerosis	bta05418	3	0.037450051
Oxytocin signaling pathway	bta04921	3	0.042011594
Measles	bta05162	3	0.042011594
Amphetamine addiction	bta05031	2	0.046054074

Supplementary Table 7. The KEGG enrichment analysis of differential expression genes in Plasma cells between BCoV and CON groups

#Term	ID	Input number	Corrected P-Value
Protein processing in endoplasmic reticulum	bta04141	41	5.35E-22
Prion disease	bta05020	38	1.34E-10
Parkinson disease	bta05012	34	1.86E-08
Antigen processing and presentation	bta04612	19	9.43E-08
Coronavirus disease - COVID-19	bta05171	48	5.71E-07
Chemical carcinogenesis - reactive oxygen species	bta05208	30	7.56E-07
Oxidative phosphorylation	bta00190	23	1.26E-06
Thermogenesis	bta04714	29	1.27E-06
Epstein-Barr virus infection	bta05169	27	2.84E-06
Diabetic cardiomyopathy	bta05415	25	1.07E-05
Alzheimer disease	bta05010	34	1.55E-05
N-Glycan biosynthesis	bta00510	12	1.67E-05
Phagosome	bta04145	21	1.77E-05
Leishmaniasis	bta05140	14	2.52E-05
Huntington disease	bta05016	29	4.62E-05
Various types of N-glycan biosynthesis	bta00513	10	7.21E-05
Pathways of neurodegeneration - multiple diseases	bta05022	35	0.000143843
Ribosome	bta03010	35	0.000143844
Amyotrophic lateral sclerosis	bta05014	31	0.000149074
Viral myocarditis	bta05416	14	0.000152767
Allograft rejection	bta05330	12	0.000286324
Protein export	bta03060	7	0.000337283
Estrogen signaling pathway	bta04915	15	0.000340599
Human T-cell leukemia virus 1 infection	bta05166	22	0.000348519
Intestinal immune network for IgA production	bta04672	12	0.000530676
Autoimmune thyroid disease	bta05320	12	0.000828362
Spliceosome	bta03040	13	0.000871151
Asthma	bta05310	10	0.000939264
Human cytomegalovirus infection	bta05163	20	0.000947166
Prostate cancer	bta05215	12	0.001011902
Herpes simplex virus 1 infection	bta05168	19	0.001129206
B cell receptor signaling pathway	bta04662	12	0.001182018
MicroRNAs in cancer	bta05206	15	0.001216301
Fc epsilon RI signaling pathway	bta04664	10	0.001374428
Yersinia infection	bta05135	16	0.00169107
MAPK signaling pathway - fly	bta04013	10	0.002402352
Thyroid hormone signaling pathway	bta04919	12	0.002791988

Staphylococcus aureus infection	bta05150	14	0.003308729
Systemic lupus erythematosus	bta05322	14	0.003757701
Transcriptional misregulation in cancer	bta05202	17	0.004945867
Lipid and atherosclerosis	bta05417	17	0.005441055
ATP-dependent chromatin remodeling	bta03082	10	0.005995716
Rheumatoid arthritis	bta05323	13	0.006015622
NF-kappa B signaling pathway	bta04064	13	0.009048404
Measles	bta05162	14	0.010972533
African trypanosomiasis	bta05143	7	0.011046622
Retrograde endocannabinoid signaling	bta04723	13	0.011108167
Tuberculosis	bta05152	15	0.014899509
Toxoplasmosis	bta05145	10	0.015724436
Apoptosis - fly	bta04214	6	0.015902786
Viral carcinogenesis	bta05203	14	0.01787871
Propanoate metabolism	bta00640	5	0.026613165
Proteasome	bta03050	6	0.027733418
Fc gamma R-mediated phagocytosis	bta04666	9	0.030327022
Fluid shear stress and atherosclerosis	bta05418	11	0.030379717
Hematopoietic cell lineage	bta04640	11	0.03369498
RNA degradation	bta03018	7	0.035288293
Salmonella infection	bta05132	15	0.036991755
Primary immunodeficiency	bta05340	6	0.038699176
Breast cancer	bta05224	11	0.049630342

Supplementary Table 8. The KEGG enrichment analysis of differential expression genes in CD4^{+}T cells between BCoV and CON groups

#Term	ID	Input number	Corrected P-Value
Ribosome	bta03010	63	2.67E-49
Huntington disease	bta05016	49	2.45E-30
Parkinson disease	bta05012	43	5.45E-29
Oxidative phosphorylation	bta00190	42	5.45E-29
Alzheimer disease	bta05010	41	2.04E-24
Thermogenesis	bta04714	45	6.34E-24
Proteasome	bta03050	22	1.09E-18
Non-alcoholic fatty liver disease (NAFLD)	bta04932	31	6.27E-17
Metabolic pathways	bta01100	77	3.35E-10
Cardiac muscle contraction	bta04260	16	3.05E-08
Retrograde endocannabinoid signaling	bta04723	20	3.05E-08
Spliceosome	bta03040	18	1.25E-07
T cell receptor signaling pathway	bta04660	16	2.28E-07
PD-L1 expression and PD-1 checkpoint pathway in cancer	bta05235	14	1.61E-06
Natural killer cell mediated cytotoxicity	bta04650	16	2.43E-06
Epstein-Barr virus infection	bta05169	21	2.43E-06
Fc gamma R-mediated phagocytosis	bta04666	13	9.15E-06
Human immunodeficiency virus 1 infection	bta05170	20	1.25E-05
Th1 and Th2 cell differentiation	bta04658	13	1.25E-05
Endocytosis	bta04144	20	2.33E-05
RNA transport	bta03013	16	2.78E-05
Cellular senescence	bta04218	16	3.07E-05
Regulation of actin cytoskeleton	bta04810	18	3.76E-05
Human T-cell leukemia virus 1 infection	bta05166	18	0.000127325
Rap1 signaling pathway	bta04015	16	0.000561133
Primary immunodeficiency	bta05340	7	0.000563919
HIF-1 signaling pathway	bta04066	11	0.000663173
Bacterial invasion of epithelial cells	bta05100	9	0.000742517
Th17 cell differentiation	bta04659	11	0.000771294
RNA polymerase	bta03020	6	0.000838696
Platelet activation	bta04611	11	0.00126331
NF-kappa B signaling pathway	bta04064	10	0.001375188
Ras signaling pathway	bta04014	16	0.001463477
Salmonella infection	bta05132	9	0.001601811
Oxytocin signaling pathway	bta04921	12	0.00193943
Pathways in cancer	bta05200	26	0.002283171
Leukocyte transendothelial migration	bta04670	10	0.002481533
Carbon metabolism	bta01200	10	0.002481533

Endocrine and other factor-regulated calcium reabsorption	bta04961	6	0.005918913
Measles	bta05162	11	0.005929
mTOR signaling pathway	bta04150	11	0.006390543
Hematopoietic cell lineage	bta04640	9	0.007308629
Long-term potentiation	bta04720	7	0.007943495
Apelin signaling pathway	bta04371	10	0.009932879
Apoptosis	bta04210	10	0.010714011
cGMP-PKG signaling pathway	bta04022	11	0.011422425
Phagosome	bta04145	11	0.011675016
Pyrimidine metabolism	bta00240	6	0.01209491
Glioma	bta05214	7	0.012446496
Hepatocellular carcinoma	bta05225	11	0.012791976
Synaptic vesicle cycle	bta04721	7	0.012791976
VEGF signaling pathway	bta04370	6	0.012988964
Proteoglycans in cancer	bta05205	12	0.012988964
Thyroid cancer	bta05216	5	0.013544137
MAPK signaling pathway	bta04010	15	0.01499893
Yersinia infection	bta05135	9	0.016644951
Cell adhesion molecules (CAMs)	bta04514	10	0.018186778
Glycolysis / Gluconeogenesis	bta00010	6	0.018454863
Purine metabolism	bta00230	9	0.01901218
Central carbon metabolism in cancer	bta05230	6	0.019120834
Human cytomegalovirus infection	bta05163	13	0.019120834
Acute myeloid leukemia	bta05221	6	0.019838181
Pentose phosphate pathway	bta00030	4	0.019976531
Non-small cell lung cancer	bta05223	6	0.020562594
Gap junction	bta04540	7	0.020918696
Inflammatory bowel disease (IBD)	bta05321	6	0.022405887
Adherens junction	bta04520	6	0.022405887
Thyroid hormone signaling pathway	bta04919	8	0.024158328
Fluid shear stress and atherosclerosis	bta05418	9	0.0250399
Biosynthesis of amino acids	bta01230	6	0.027535166
Influenza A	bta05164	10	0.030326727
Rheumatoid arthritis	bta05323	7	0.034634198
Melanogenesis	bta04916	7	0.034634198
RNA degradation	bta03018	6	0.036772207
Glucagon signaling pathway	bta04922	7	0.037038714
EGFR tyrosine kinase inhibitor resistance	bta01521	6	0.037801885
PI3K-Akt signaling pathway	bta04151	16	0.039591467
Vascular smooth muscle contraction	bta04270	8	0.040130298
Ribosome biogenesis in eukaryotes	bta03008	6	0.042583425
Long-term depression	bta04730	5	0.043402993
Insulin signaling pathway	bta04910	8	0.044610095

Protein processing in endoplasmic reticulum	bta04141	9	0.044610095
B cell receptor signaling pathway	bta04662	6	0.047122341
Focal adhesion	bta04510	10	0.04935954

Supplementary Table 9. The proportion of sub-cell types of CD4+T cells in jejunum mucosal in calves

Celltype	BCoV	CON
Th17	0.45218677	0.234631153
Treg	0.017718319	0.087202295
Th1	0.243120141	0.051203208
Activated CD4+T	0.205728049	0.475019288
Proliferating CD4+T	0.081246721	0.151944055

Supplementary Table 10. The KEGG enrichment analysis of differential expression genes in Th17 between BCoV and CON groups

#Term	ID	Input number	Corrected P-Value
Prion disease	bta05020	64	1.16E-34
Parkinson disease	bta05012	58	1.71E-29
Alzheimer disease	bta05010	63	6.84E-26
Huntington disease	bta05016	57	1.99E-25
Oxidative phosphorylation	bta00190	42	2.03E-24
Amyotrophic lateral sclerosis	bta05014	60	7.02E-24
Diabetic cardiomyopathy	bta05415	45	3.55E-22
Chemical carcinogenesis - reactive oxygen species	bta05208	47	7.74E-21
Pathways of neurodegeneration - multiple diseases	bta05022	61	2.16E-20
Thermogenesis	bta04714	42	2.40E-17
Non-alcoholic fatty liver disease	bta04932	34	6.55E-17
Ribosome	bta03010	54	7.45E-16
Proteasome	bta03050	18	2.82E-15
Coronavirus disease - COVID-19	bta05171	56	5.14E-13
Epstein-Barr virus infection	bta05169	26	2.07E-07
Retrograde endocannabinoid signaling	bta04723	19	5.35E-07
Spinocerebellar ataxia	bta05017	17	1.36E-06
Protein processing in endoplasmic reticulum	bta04141	19	2.19E-06
Primary immunodeficiency	bta05340	9	4.35E-05
Yersinia infection	bta05135	17	4.59E-05
T cell receptor signaling pathway	bta04660	15	5.22E-05
RNA degradation	bta03018	10	5.78E-05
Spliceosome	bta03040	13	6.39E-05
Cardiac muscle contraction	bta04260	12	0.000129472
Herpes simplex virus 1 infection	bta05168	18	0.000265942
Th1 and Th2 cell differentiation	bta04658	12	0.000315618
Apoptosis	bta04210	13	0.000414153
Human T-cell leukemia virus 1 infection	bta05166	19	0.000454717
Hematopoietic cell lineage	bta04640	13	0.0005702
Natural killer cell mediated cytotoxicity	bta04650	14	0.000769778
PD-L1 expression and PD-1 checkpoint pathway in cancer	bta05235	11	0.0007565
Thyroid hormone signaling pathway	bta04919	11	0.001065457
NF-kappa B signaling pathway	bta04064	13	0.00110949
Measles	bta05162	14	0.001395659
Influenza A	bta05164	15	0.001565394
Antigen processing and presentation	bta04612	10	0.002106076
Apoptosis - fly	bta04214	6	0.002379735
Intestinal immune network for IgA production	bta04672	9	0.002767397

Th17 cell differentiation	bta04659	11	0.002857969
Fluid shear stress and atherosclerosis	bta05418	11	0.004835173
Central carbon metabolism in cancer	bta05230	7	0.006098476
Salmonella infection	bta05132	15	0.006000318
Allograft rejection	bta05330	8	0.005997306
Virion - Human immunodeficiency virus	bta03260	2	0.007631491
Human cytomegalovirus infection	bta05163	15	0.008547895
Bacterial invasion of epithelial cells	bta05100	6	0.010414095
HIF-1 signaling pathway	bta04066	9	0.010983919
Autoimmune thyroid disease	bta05320	8	0.012476061
Pentose phosphate pathway	bta00030	4	0.012170415
Protein export	bta03060	4	0.012170415
Ribosome biogenesis in eukaryotes	bta03008	7	0.013866773
MAPK signaling pathway - fly	bta04013	7	0.014913878
Rheumatoid arthritis	bta05323	10	0.017324189
Platinum drug resistance	bta01524	7	0.017174815
B cell receptor signaling pathway	bta04662	8	0.017173532
Viral myocarditis	bta05416	8	0.020537831
Asthma	bta05310	6	0.0254452
mTOR signaling pathway	bta04150	10	0.024260179
Hepatitis C	bta05160	11	0.026151303
Necroptosis	bta04217	11	0.026151303
Peroxisome	bta04146	7	0.025388803
Leishmaniasis	bta05140	7	0.025388803
Inflammatory bowel disease	bta05321	7	0.028637126
Nucleotide metabolism	bta01232	7	0.028637126
Circadian rhythm - fly	bta04711	2	0.032309269
Mitophagy - animal	bta04137	7	0.034018434
Chagas disease	bta05142	9	0.0335407
Cell adhesion molecules	bta04514	12	0.035637172
Prolactin signaling pathway	bta04917	6	0.0355224
Drug metabolism - other enzymes	bta00983	6	0.042646658
Regulation of actin cytoskeleton	bta04810	12	0.04100175
Sulfur relay system	bta04122	2	0.04054937
Type I diabetes mellitus	bta04940	6	0.04017981
Fc gamma R-mediated phagocytosis	bta04666	7	0.042181899
Prostate cancer	bta05215	7	0.042181899
Osteoclast differentiation	bta04380	9	0.049276112
IL-17 signaling pathway	bta04657	7	0.049072213

Supplementary Table 11. The KEGG enrichment analysis of differential expression genes in Treg between BCoV and CON groups

#Term	ID	Input number	Corrected P-Value
Human T-cell leukemia virus 1 infection	bta05166	20	2.13E-06
DNA replication	bta03030	7	5.07E-05
Th1 and Th2 cell differentiation	bta04658	10	0.000397128
Th17 cell differentiation	bta04659	10	0.000964006
Cellular senescence	bta04218	10	0.002920109
Mismatch repair	bta03430	4	0.003077371
SNARE interactions in vesicular transport	bta04130	5	0.003774992
Glycosaminoglycan biosynthesis - keratan sulfate	bta00533	3	0.004555849
Homologous recombination	bta03440	5	0.004702164
Apoptosis	bta04210	9	0.004942142
Endocrine resistance	bta01522	7	0.00541074
Pathways of neurodegeneration - multiple diseases	bta05022	21	0.005437542
Cell cycle	bta04110	10	0.005443754
Hepatocellular carcinoma	bta05225	10	0.005443754
Inflammatory bowel disease	bta05321	7	0.006200467
Asthma	bta05310	6	0.006399805
Influenza A	bta05164	11	0.00677979
Glycosphingolipid biosynthesis - ganglio series	bta00604	3	0.006963535
Coronavirus disease - COVID-19	bta05171	24	0.008241041
Measles	bta05162	10	0.008265312
Chronic myeloid leukemia	bta05220	6	0.009961403
Mucin type O-glycan biosynthesis	bta00512	4	0.010329141
Prion disease	bta05020	14	0.012718591
Antigen processing and presentation	bta04612	7	0.012853018
Phagosome	bta04145	10	0.013105014
Glycosaminoglycan biosynthesis - heparan sulfate / heparin	bta00534	3	0.013693733
Pyrimidine metabolism	bta00240	5	0.013850793
Thermogenesis	bta04714	13	0.014071467
Huntington disease	bta05016	15	0.014824984
Parathyroid hormone synthesis, secretion and action	bta04928	7	0.015106833
Alzheimer disease	bta05010	17	0.01689286
Salmonella infection	bta05132	11	0.018170984
Base excision repair	bta03410	4	0.018691534
Epstein-Barr virus infection	bta05169	12	0.018778207
Efferocytosis	bta04148	9	0.023060306
AGE-RAGE signaling pathway in diabetic complications	bta04933	6	0.024601113
Relaxin signaling pathway	bta04926	7	0.02694591
Diabetic cardiomyopathy	bta05415	11	0.028357684

Ubiquinone and other terpenoid-quinone biosynthesis	bta00130	2	0.033976611
Prolactin signaling pathway	bta04917	5	0.034430788
Chemical carcinogenesis - reactive oxygen species	bta05208	12	0.037200921
Estrogen signaling pathway	bta04915	7	0.037645245
Type I diabetes mellitus	bta04940	5	0.03838092
Oocyte meiosis	bta04114	6	0.039840905
Amino sugar and nucleotide sugar metabolism	bta00520	4	0.040006947
Endocytosis	bta04144	10	0.041419044
Cholinergic synapse	bta04725	6	0.041631731
Toxoplasmosis	bta05145	6	0.041631731
MAPK signaling pathway - fly	bta04013	5	0.042596878
Ether lipid metabolism	bta00565	4	0.042670479
Gap junction	bta04540	5	0.047080966
p53 signaling pathway	bta04115	5	0.047080966
Hepatitis B	bta05161	8	0.04745669
Porphyrin metabolism	bta00860	4	0.048290268
Parkinson disease	bta05012	12	0.048917656
Protein processing in endoplasmic reticulum	bta04141	8	0.049025798
Glycerophospholipid metabolism	bta00564	6	0.049297362
Growth hormone synthesis, secretion and action	bta04935	6	0.049297362
Rap1 signaling pathway	bta04015	10	0.055984148

Supplementary Table 12. Results of the KEGG database alignment of the *Phocaeicola coprophilus* genome

GENE	KO	NAME
WP_002558051.1	K02919	RP-L36, MRPL36, rpmJ; large subunit ribosomal protein L36
WP_002558052.1	K02518	infA; translation initiation factor IF-1
WP_004293585.1	K02014	TC.FEV.OM; iron complex outermembrane recepter protein
WP_004293586.1	K25034	btuF; cobalamin transport system substrate-binding protein
WP_004293588.1	K25028	btuD; cobalamin transport system ATP-binding protein [EC:7.6.2.8]
WP_004293619.1	K06926	K06926; uncharacterized protein
WP_004310157.1	K03111	ssb; single-strand DNA-binding protein
WP_004323414.1	K00986	ltrA; RNA-directed DNA polymerase [EC:2.7.7.49]
WP_005816141.1	K01185	E3.2.1.17; lysozyme [EC:3.2.1.17]
WP_005816273.1	K07133	K07133; uncharacterized protein
WP_005816294.1	K03671	TXN, trxA; thioredoxin
WP_005816319.1	K03496	parA, soj; chromosome partitioning protein
WP_005816321.1	K03111	ssb; single-strand DNA-binding protein
WP_005841178.1	K02963	RP-S18, MRPS18, rpsR; small subunit ribosomal protein S18
WP_005841291.1	K02913	RP-L33, MRPL33, rpmG; large subunit ribosomal protein L33
WP_007559377.1	K02874	RP-L14, MRPL14, rplN; large subunit ribosomal protein L14
WP_007559383.1	K02890	RP-L22, MRPL22, rplV; large subunit ribosomal protein L22
WP_007559385.1	K02965	RP-S19, RSM19, rpsS; small subunit ribosomal protein S19
WP_007559879.1	K02911	RP-L32, MRPL32, rpmF; large subunit ribosomal protein L32
WP_007560904.1	K00986	ltrA; RNA-directed DNA polymerase [EC:2.7.7.49]
WP_007562676.1	K00134	GAPDH, gapA; glyceraldehyde 3-phosphate dehydrogenase (phosphorylating) [EC:1.2.1.12]
WP_007564314.1	K02914	RP-L34, MRPL34, rpmH; large subunit ribosomal protein L34
WP_007567343.1	K02946	RP-S10, MRPS10, rpsJ; small subunit ribosomal protein S10
WP_007567382.1	K02948	RP-S11, MRPS11, rpsK; small subunit ribosomal protein S11
WP_007567505.1	K02078	acpP, acpM; acyl carrier protein
WP_007571099.1	K02909	RP-L31, rpmE; large subunit ribosomal protein L31

WP_007660154.1	K07484	K07484; transposase
WP_007896781.1	K03496	parA, soj; chromosome partitioning protein
WP_007897432.1	K07484	K07484; transposase
WP_007897433.1	K07484	K07484; transposase
WP_007901159.1	K07484	K07484; transposase
WP_008139685.1	K03581	recD; exodeoxyribonuclease V alpha subunit [EC:3.1.11.5]
WP_008139687.1	K03424	tatD; TatD DNase family protein [EC:3.1.21.-]
WP_008139692.1	K01338	lon; ATP-dependent Lon protease [EC:3.4.21.53]
WP_008139731.1	K06926	K06926; uncharacterized protein
WP_008139740.1	K01546	kdpA; potassium-transporting ATPase potassium-binding subunit
WP_008139741.1	K01547	kdpB; potassium-transporting ATPase ATP-binding subunit [EC:7.2.2.6]
WP_008139743.1	K01548	kdpC; potassium-transporting ATPase KdpC subunit
WP_008139748.1	K07646	kdpD; two-component system, OmpR family, sensor histidine kinase KdpD [EC:2.7.13.3]
WP_008139783.1	K03217	yidC, spoIIIJ, OXA1, ccfA; YidC/Oxa1 family membrane protein insertase
WP_008139785.1	K01937	pyrG, CTPS; CTP synthase [EC:6.3.4.2]
WP_008139803.1	K00610	pyrI; aspartate carbamoyltransferase regulatory subunit
WP_008139804.1	K00609	pyrB, PYR2; aspartate carbamoyltransferase catalytic subunit [EC:2.1.3.2]
WP_008139807.1	K03427	hsdM; type I restriction enzyme M protein [EC:2.1.1.72]
WP_008139808.1	K01154	hsdS; type I restriction enzyme, S subunit [EC:3.1.21.3]
WP_008139816.1	K01153	hsdR; type I restriction enzyme, R subunit [EC:3.1.21.3]
WP_008139821.1	K01190	lacZ; beta-galactosidase [EC:3.2.1.23]
WP_008139845.1	K03100	lepB; signal peptidase I [EC:3.4.21.89]
WP_008139867.1	K22431	carD; caffeyl-CoA reductase-Etf complex subunit CarD [EC:1.3.1.108]
WP_008139869.1	K28018	pbfC; 2-(methylaminoethyl)phosphonate dehydrogenase (acceptor) [EC:1.5.99.16]
WP_008139874.1	K02217	ftnA, ftn; ferritin [EC:1.16.3.2]
WP_008139893.1	K03321	TC.SULP; sulfate permease, SulP family
WP_008139897.1	K01689	ENO1_2_3, eno; enolase 1/2/3 [EC:4.2.1.11]
WP_008139924.1	K01156	res; type III restriction enzyme [EC:3.1.21.5]
WP_008139926.1	K07316	mod; adenine-specific DNA-methyltransferase [EC:2.1.1.72]
WP_008139942.1	K02426	sufE; cysteine desulfuration protein SufE
WP_008139945.1	K00634	ptb; phosphate butyryltransferase [EC:2.3.1.19]
WP_008139947.1	K18672	dacA; diadenylate cyclase [EC:2.7.7.85]
WP_008139949.1	K00796	folP; dihydropteroate synthase [EC:2.5.1.15]

WP_008139954.1	K28370	lysO; lysine exporter
WP_008139974.1	K01866	YARS, tyrS; tyrosyl-tRNA synthetase [EC:6.1.1.1]
WP_008139975.1	K03424	tatD; TatD DNase family protein [EC:3.1.21.-]
WP_008139976.1	K08998	K08998; uncharacterized protein
WP_008139977.1	K03536	rnpA; ribonuclease P protein component [EC:3.1.26.5]
WP_008139979.1	K01719	hemD, UROS; uroporphyrinogen-III synthase [EC:4.2.1.75]
WP_008139984.1	K00789	metK, MAT; S-adenosylmethionine synthetase [EC:2.5.1.6]
WP_008139986.1	K00950	folK; 2-amino-4-hydroxy-6-hydroxymethyldihydropteridine diphosphokinase [EC:2.7.6.3]
WP_008139988.1	K07568	queA; S-adenosylmethionine:tRNA ribosyltransferase-isomerase [EC:2.4.99.17]
WP_008139990.1	K03177	truB, PUS4, TRUB1; tRNA pseudouridine55 synthase [EC:5.4.99.25]
WP_008139992.1	K06153	bacA; undecaprenyl-diphosphatase [EC:3.6.1.27]
WP_008139995.1	K09811	ftsX; cell division transport system permease protein
WP_008140000.1	K06168	CDK5RAP1, miaB; tRNA-2-methylthio-N6-dimethylallyladenosine synthase [EC:2.8.4.3]
WP_008140009.1	K01869	LARS, leuS; leucyl-tRNA synthetase [EC:6.1.1.4]
WP_008140016.1	K01519	rdgB, ITPA; XTP/dITP diphosphohydrolase [EC:3.6.1.66]
WP_008140019.1	K00077	panE, apbA; 2-dehydropantoate 2-reductase [EC:1.1.1.169]
WP_008140021.1	K06942	ychF; ribosome-binding ATPase
WP_008140030.1	K02523	ispB; octaprenyl-diphosphate synthase [EC:2.5.1.90]
WP_008140032.1	K01619	deoC, DERA; deoxyribose-phosphate aldolase [EC:4.1.2.4]
WP_008140037.1	K03703	uvrC; excinuclease ABC subunit C
WP_008140038.1	K00759	APRT, apt; adenine phosphoribosyltransferase [EC:2.4.2.7]
WP_008140039.1	K03495	gidA, mnmG, MTO1; tRNA uridine 5-carboxymethylaminomethyl modification enzyme
WP_008140050.1	K28560	bcerS; bacterial ceramide synthase [EC:2.3.1.-]
WP_008140055.1	K00954	E2.7.7.3A, coaD, kdtB; pantetheine-phosphate adenylyltransferase [EC:2.7.7.3]
WP_008140058.1	K00241	sdhC, frdC; succinate dehydrogenase cytochrome b subunit
WP_008140060.1	K00239	sdhA, frdA; succinate dehydrogenase flavoprotein subunit [EC:1.3.5.1]
WP_008140061.1	K00240	sdhB, frdB; succinate dehydrogenase iron-sulfur subunit [EC:1.3.5.1]
WP_008140063.1	K04096	smf; DNA processing protein

WP_008140065.1	K07107	ybgC; acyl-CoA thioester hydrolase [EC:3.1.2.-]
WP_008140072.1	K03100	lepB; signal peptidase I [EC:3.4.21.89]
WP_008140075.1	K03100	lepB; signal peptidase I [EC:3.4.21.89]
WP_008140080.1	K01190	lacZ; beta-galactosidase [EC:3.2.1.23]
WP_008140082.1	K07059	rho2; rhomboid family protease [EC:3.4.21.105]
WP_008140086.1	K01284	dcp; peptidyl-dipeptidase Dcp [EC:3.4.15.5]
WP_008140088.1	K12257	secDF; SecD/SecF fusion protein
WP_008140091.1	K00639	kbl, GCAT; glycine C-acetyltransferase [EC:2.3.1.29]
WP_008140092.1	K00174	korA, oorA, oforA; 2-oxoglutarate/2-oxoacid ferredoxin oxidoreductase subunit alpha [EC:1.2.7.3 1.2.7.11]
WP_008140093.1	K00175	korB, oorB, oforB; 2-oxoglutarate/2-oxoacid ferredoxin oxidoreductase subunit beta [EC:1.2.7.3 1.2.7.11]
WP_008140094.1	K00761	upp, UPRT; uracil phosphoribosyltransferase [EC:2.4.2.9]
WP_008140096.1	K01610	pckA; phosphoenolpyruvate carboxykinase (ATP) [EC:4.1.1.49]
WP_008140103.1	K03469	rnhA, RNASEH1; ribonuclease HI [EC:3.1.26.4]
WP_008140106.1	K01887	RARS, argS; arginyl-tRNA synthetase [EC:6.1.1.19]
WP_008140119.1	K06041	kdsD, kpsF; arabinose-5-phosphate isomerase [EC:5.3.1.13]
WP_008140125.1	K03551	ruvB; holliday junction DNA helicase RuvB [EC:5.6.2.4]
WP_008140128.1	K05592	deaD, cshA; ATP-dependent RNA helicase DeaD [EC:5.6.2.7]
WP_008140129.1	K07727	K07727; putative transcriptional regulator
WP_008140131.1	K07052	K07052; CAAX protease family protein
WP_008140133.1	K11753	ribF; riboflavin kinase / FMN adenylyltransferase [EC:2.7.1.26 2.7.7.2]
WP_008140135.1	K20866	yihX; glucose-1-phosphatase [EC:3.1.3.10]
WP_008140154.1	K24118	atsB, chuR, aslB; serine-type anaerobic sulfatase-maturating enzyme [EC:1.1.98.7]
WP_008140160.1	K02527	kdtA, waaA; 3-deoxy-D-manno-octulosonic-acid transferase [EC:2.4.99.12 2.4.99.13 2.4.99.14 2.4.99.15]
WP_008140162.1	K01262	pepP; Xaa-Pro aminopeptidase [EC:3.4.11.9]
WP_008140163.1	K02970	RP-S21, MRPS21, rpsU; small subunit ribosomal protein S21
WP_008140167.1	K02358	tuf, TUFM; elongation factor Tu
WP_008140168.1	K03073	secE; preprotein translocase subunit SecE
WP_008140169.1	K02601	nusG; transcription termination/antitermination protein NusG
WP_008140170.1	K02867	RP-L11, MRPL11, rplK; large subunit ribosomal protein L11
WP_008140171.1	K02863	RP-L1, MRPL1, rplA; large subunit ribosomal protein L1

WP_008140172.1	K02864	RP-L10, MRPL10, rplJ; large subunit ribosomal protein L10
WP_008140174.1	K02935	RP-L7, MRPL12, rplL; large subunit ribosomal protein L7/L12
WP_008140175.1	K03043	rpoB; DNA-directed RNA polymerase subunit beta [EC:2.7.7.6]
WP_008140176.1	K03046	rpoC; DNA-directed RNA polymerase subunit beta' [EC:2.7.7.6]
WP_008140184.1	K21572	susD; starch-binding outer membrane protein, SusD/RagB family
WP_008140199.1	K03118	tatC; sec-independent protein translocase protein TatC
WP_008140200.1	K02950	RP-S12, MRPS12, rpsL; small subunit ribosomal protein S12
WP_008140201.1	K02992	RP-S7, MRPS7, rpsG; small subunit ribosomal protein S7
WP_008140203.1	K02355	fusA, GFM, EFG; elongation factor G
WP_008140206.1	K02906	RP-L3, MRPL3, rplC; large subunit ribosomal protein L3
WP_008140209.1	K02926	RP-L4, MRPL4, rplD; large subunit ribosomal protein L4
WP_008140211.1	K02892	RP-L23, MRPL23, rplW; large subunit ribosomal protein L23
WP_008140212.1	K02886	RP-L2, MRPL2, RML2, rplB; large subunit ribosomal protein L2
WP_008140217.1	K02982	RP-S3, rpsC; small subunit ribosomal protein S3
WP_008140219.1	K02878	RP-L16, MRPL16, rplP; large subunit ribosomal protein L16
WP_008140221.1	K02904	RP-L29, rpmC; large subunit ribosomal protein L29
WP_008140227.1	K02895	RP-L24, MRPL24, rplX; large subunit ribosomal protein L24
WP_008140232.1	K02954	RP-S14, MRPS14, rpsN; small subunit ribosomal protein S14
WP_008140234.1	K02994	RP-S8, MRPS8, rpsH; small subunit ribosomal protein S8
WP_008140236.1	K02933	RP-L6, MRPL6, rplF; large subunit ribosomal protein L6
WP_008140238.1	K02881	RP-L18, MRPL18, rplR; large subunit ribosomal protein L18
WP_008140239.1	K02988	RP-S5, MRPS5, rpsE; small subunit ribosomal protein S5
WP_008140242.1	K02907	RP-L30, MRPL30, rpmD; large subunit ribosomal protein L30
WP_008140244.1	K02876	RP-L15, MRPL15, rplO; large subunit ribosomal protein L15

WP_008140249.1	K01265	map; methionyl aminopeptidase [EC:3.4.11.18]
WP_008140252.1	K02952	RP-S13, rpsM; small subunit ribosomal protein S13
WP_008140255.1	K02986	RP-S4, NAM9, rpsD; small subunit ribosomal protein S4
WP_008140256.1	K03040	rpoA; DNA-directed RNA polymerase subunit alpha [EC:2.7.7.6]
WP_008140257.1	K02879	RP-L17, MRPL17, rplQ; large subunit ribosomal protein L17
WP_008140258.1	K00962	pnp, PNPT1; polyribonucleotide nucleotidyltransferase [EC:2.7.7.8]
WP_008140261.1	K03624	greA; transcription elongation factor GreA
WP_008140262.1	K02503	HINT1_2, hinT, hit; histidine triad (HIT) family protein [EC:3.9.1.-]
WP_008140266.1	K00971	manC, cpsB; mannose-1-phosphate guanylyltransferase [EC:2.7.7.13]
WP_008140288.1	K04759	feoB; ferrous iron transport protein B
WP_008140292.1	K13993	HSP20; HSP20 family protein
WP_008140296.1	K03498	trkH, trkG, ktrB, ktrD; trk/ktr system potassium uptake protein
WP_008140297.1	K12251	aguB; N-carbamoylputrescine amidase [EC:3.5.1.53]
WP_008140307.1	K01372	BLMH, pepC; bleomycin hydrolase [EC:3.4.22.40]
WP_008140309.1	K00346	nqrA; Na+-transporting NADH:ubiquinone oxidoreductase subunit A [EC:7.2.1.1]
WP_008140311.1	K00347	nqrB; Na+-transporting NADH:ubiquinone oxidoreductase subunit B [EC:7.2.1.1]
WP_008140315.1	K00349	nqrD; Na+-transporting NADH:ubiquinone oxidoreductase subunit D [EC:7.2.1.1]
WP_008140317.1	K00350	nqrE; Na+-transporting NADH:ubiquinone oxidoreductase subunit E [EC:7.2.1.1]
WP_008140318.1	K00351	nqrF; Na+-transporting NADH:ubiquinone oxidoreductase subunit F [EC:7.2.1.1]
WP_008140326.1	K00971	manC, cpsB; mannose-1-phosphate guanylyltransferase [EC:2.7.7.13]
WP_008140342.1	K07478	ycaJ; putative ATPase
WP_008140345.1	K00018	hprA; glycerate dehydrogenase [EC:1.1.1.29]
WP_008140351.1	K03596	lepA; GTP-binding protein LepA
WP_008140354.1	K03616	rnfB; H+/Na+-translocating ferredoxin:NAD+ oxidoreductase subunit B [EC:7.1.1.11 7.2.1.2]
WP_008140355.1	K03615	rnfC; H+/Na+-translocating ferredoxin:NAD+ oxidoreductase subunit C [EC:7.1.1.11 7.2.1.2]
WP_008140356.1	K03614	rnfD; H+/Na+-translocating ferredoxin:NAD+ oxidoreductase subunit D [EC:7.1.1.11 7.2.1.2]
WP_008140357.1	K03612	rnfG; H+/Na+-translocating ferredoxin:NAD+ oxidoreductase subunit G

WP_008140358.1	K03613	rnfE; H+/Na+-translocating ferredoxin:NAD+ oxidoreductase subunit E
WP_008140359.1	K03617	rnfA; H+/Na+-translocating ferredoxin:NAD+ oxidoreductase subunit A
WP_008140361.1	K01784	galE, GALE; UDP-glucose 4-epimerase [EC:5.1.3.2]
WP_008140364.1	K26937	dinF, mepA, vmrA; MATE family, multidrug efflux pump
WP_008140373.1	K01533	copB; P-type Cu2+ transporter [EC:7.2.2.9]
WP_008140376.1	K07085	K07085; putative transport protein
WP_008140381.1	K08218	ampG; MFS transporter, PAT family, beta-lactamase induction signal transducer AmpG
WP_008140389.1	K06941	rlmN; 23S rRNA (adenine2503-C2)-methyltransferase [EC:2.1.1.192]
WP_008140410.1	K01858	INO1, ISYNA1; myo-inositol-1-phosphate synthase [EC:5.5.1.4]
WP_008140421.1	K03075	secG; preprotein translocase subunit SecG
WP_008140426.1	K08676	tri; tricorn protease [EC:3.4.21.-]
WP_008140432.1	K07447	ruvX; putative pre-16S rRNA nuclease [EC:3.1.-.-]
WP_008140434.1	K01462	PDF, def; peptide deformylase [EC:3.5.1.88]
WP_008140440.1	K02520	infC, MTIF3; translation initiation factor IF-3
WP_008140442.1	K02916	RP-L35, MRPL35, rpmI; large subunit ribosomal protein L35
WP_008140443.1	K02887	RP-L20, MRPL20, rplT; large subunit ribosomal protein L20
WP_008140450.1	K01173	ENDOG; endonuclease G, mitochondrial
WP_008140464.1	K07001	K07001; NTE family protein
WP_008140465.1	K00645	fabD, MCAT, MCT1; [acyl-carrier-protein] S-malonyltransferase [EC:2.3.1.39]
WP_008140479.1	K01897	ACSL, fadD; long-chain-fatty-acid---CoA ligase [EC:6.2.1.3]
WP_008140483.1	K01159	ruvC; crossover junction endodeoxyribonuclease RuvC [EC:3.1.21.10]
WP_008140487.1	K05776	modF; molybdate transport system ATP-binding protein
WP_008140489.1	K07791	dcuA; anaerobic C4-dicarboxylate transporter DcuA
WP_008140493.1	K01790	rfbC, rmlC; dTDP-4-dehydrorhamnose 3,5-epimerase [EC:5.1.3.13]
WP_008140494.1	K00973	rfbA, rmlA, rffH; glucose-1-phosphate thymidylyltransferase [EC:2.7.7.24]
WP_008140499.1	K02005	ABC.CD.TX; HlyD family secretion protein
WP_008140501.1	K02004	ABC.CD.P; putative ABC transport system permease protein
WP_008140514.1	K02004	ABC.CD.P; putative ABC transport system permease protein

WP_008140515.1	K02003	ABC.CD.A; putative ABC transport system ATP-binding protein
WP_008140517.1	K12340	tolC, bepC, cyaE, raxC, sapF, rsaF, hasF; outer membrane protein
WP_008140525.1	K00426	cydB; cytochrome bd ubiquinol oxidase subunit II [EC:7.1.1.7]
WP_008140535.1	K18928	lldE; L-lactate dehydrogenase complex protein LldE
WP_008140538.1	K00794	ribH, RIB4; 6,7-dimethyl-8-ribityllumazine synthase [EC:2.5.1.78]
WP_008140544.1	K03629	recF; DNA replication and repair protein RecF
WP_008140549.1	K03797	E3.4.21.102, prc, ctpA; carboxyl-terminal processing protease [EC:3.4.21.102]
WP_008140551.1	K01493	comEB; dCMP deaminase [EC:3.5.4.12]
WP_008140555.1	K01284	dcp; peptidyl-dipeptidase Dcp [EC:3.4.15.5]
WP_008140559.1	K03734	apbE; FAD:protein FMN transferase [EC:2.7.1.180]
WP_008140560.1	K23242	mntP; manganese efflux pump family protein
WP_008140575.1	K04069	pflA, pflC, pflE; pyruvate formate lyase activating enzyme [EC:1.97.1.4]
WP_008140577.1	K00656	E2.3.1.54, pflD; formate C-acetyltransferase [EC:2.3.1.54]
WP_008140586.1	K01854	glf; UDP-galactopyranose mutase [EC:5.4.99.9]
WP_008140592.1	K03218	rlmB; 23S rRNA (guanosine2251-2'-O)-methyltransferase [EC:2.1.1.185]
WP_008140594.1	K03631	recN; DNA repair protein RecN (Recombination protein N)
WP_008140598.1	K02342	dnaQ; DNA polymerase III subunit epsilon [EC:2.7.7.7]
WP_008140600.1	K02338	dnaN; DNA polymerase III subunit beta [EC:2.7.7.7]
WP_008140606.1	K03628	rho; transcription termination factor Rho
WP_008140607.1	K09022	ridA, tdcF, RIDA; 2-iminobutanoate/2-iminopropanoate deaminase [EC:3.5.99.10]
WP_008140612.1	K01759	GLO1, gloA; lactoylglutathione lyase [EC:4.4.1.5]
WP_008140617.1	K15738	uup; ABC transport system ATP-binding/permease protein
WP_008140644.1	K15475	lpnE; enhanced entry protein LpnE
WP_008140648.1	K04075	tilS, mesJ; tRNA(Ile)-lysidine synthase [EC:6.3.4.19]
WP_008140661.1	K03446	emrB; MFS transporter, DHA2 family, multidrug resistance protein
WP_008140663.1	K03543	emrA; membrane fusion protein, multidrug efflux system
WP_008140665.1	K12340	tolC, bepC, cyaE, raxC, sapF, rsaF, hasF; outer membrane protein
WP_008140669.1	K01620	ltaE; threonine aldolase [EC:4.1.2.48]
WP_008140675.1	K07005	K07005; uncharacterized protein

WP_008140683.1	K19147	mcrC; 5-methylcytosine-specific restriction enzyme subunit McrC
WP_008140689.1	K02342	dnaQ; DNA polymerase III subunit epsilon [EC:2.7.7.7]
WP_008140693.1	K03671	TXN, trxA; thioredoxin
WP_008140696.1	K24180	mleP; malate permease and related proteins
WP_008140739.1	K19171	dndD; DNA sulfur modification protein DndD
WP_008140742.1	K01156	res; type III restriction enzyme [EC:3.1.21.5]
WP_008140749.1	K03650	mnmE, trmE, MSS1; tRNA modification GTPase [EC:3.6.-.-]
WP_008140751.1	K08968	msrC; L-methionine (R)-S-oxide reductase [EC:1.8.4.14]
WP_008140752.1	K00757	udp, UPP; uridine phosphorylase [EC:2.4.2.3]
WP_008140764.1	K03811	pnuC; nicotinamide mononucleotide transporter
WP_008140765.1	K00949	thiN, TPK1, THI80; thiamine pyrophosphokinase [EC:2.7.6.2]
WP_008140768.1	K03427	hsdM; type I restriction enzyme M protein [EC:2.1.1.72]
WP_008140772.1	K01154	hsdS; type I restriction enzyme, S subunit [EC:3.1.21.3]
WP_008140777.1	K01153	hsdR; type I restriction enzyme, R subunit [EC:3.1.21.3]
WP_008140779.1	K09922	K09922; uncharacterized protein
WP_008140782.1	K13694	mepS, spr; murein DD-endopeptidase / murein LD-carboxypeptidase [EC:3.4.-.- 3.4.17.13]
WP_008140784.1	K01990	ABC-2.A; ABC-2 type transport system ATP-binding protein
WP_008140796.1	K00384	trxB, TRR; thioredoxin reductase (NADPH) [EC:1.8.1.9]
WP_008140802.1	K03466	ftsK, spoIIIE; DNA segregation ATPase FtsK/SpoIIIE, S-DNA-T family
WP_008140810.1	K25314	FKGP, fkp; fucokinase / fucose-1-phosphate guanylyltransferase [EC:2.7.1.52 2.7.7.30]
WP_008140815.1	K01711	gmd, GMDS; GDPmannose 4,6-dehydratase [EC:4.2.1.47]
WP_008140817.1	K02429	fucP, deoP, nagP, agaP; MFS transporter, FHS family, fucose-galactose-glucose:H+ symporter family protein
WP_008140845.1	K05946	tagA, tarA; N-acetylglucosaminyldiphosphoundecaprenol N-acetyl-beta-D-mannosaminyltransferase [EC:2.4.1.187]
WP_008140851.1	K19002	mgs, bgsB; 1,2-diacylglycerol 3-alpha-glucosyltransferase [EC:2.4.1.337]
WP_008140858.1	K16692	etk-wzc; tyrosine-protein kinase Etk/Wzc [EC:2.7.10.3]
WP_008140860.1	K01991	wza, gfcE; polysaccharide biosynthesis/export protein
WP_008140902.1	K03702	uvrB; excinuclease ABC subunit B
WP_008140904.1	K05807	bamD; outer membrane protein assembly factor BamD

WP_008140909.1	K26937	dinF, mepA, vmrA; MATE family, multidrug efflux pump
WP_008140910.1	K07005	K07005; uncharacterized protein
WP_008140912.1	K28370	lysO; lysine exporter
WP_008140922.1	K01938	fhs; formate--tetrahydrofolate ligase [EC:6.3.4.3]
WP_008140933.1	K05349	bglX; beta-glucosidase [EC:3.2.1.21]
WP_008140935.1	K00281	GLDC, gcvP; glycine cleavage system P protein (glycine dehydrogenase) [EC:1.4.4.2]
WP_008140936.1	K01069	gloB, gloC, HAGH; hydroxyacylglutathione hydrolase [EC:3.1.2.6]
WP_008140938.1	K03501	gidB, rsmG; 16S rRNA (guanine527-N7)-methyltransferase [EC:2.1.1.170]
WP_008140943.1	K03801	lipB; lipoyl(octanoyl) transferase [EC:2.3.1.181]
WP_008140951.1	K08999	K08999; uncharacterized protein
WP_008140952.1	K09761	rsmE; 16S rRNA (uracil1498-N3)-methyltransferase [EC:2.1.1.193]
WP_008140958.1	K02112	ATPF1B, atpD; F-type H+/Na+-transporting ATPase subunit beta [EC:7.1.2.2 7.2.2.1]
WP_008140959.1	K02114	ATPF1E, atpC; F-type H+-transporting ATPase subunit epsilon
WP_008140963.1	K02108	ATPF0A, atpB; F-type H+-transporting ATPase subunit a
WP_008140964.1	K02110	ATPF0C, atpE; F-type H+-transporting ATPase subunit c
WP_008140965.1	K02109	ATPF0B, atpF; F-type H+-transporting ATPase subunit b
WP_008140969.1	K02111	ATPF1A, atpA; F-type H+/Na+-transporting ATPase subunit alpha [EC:7.1.2.2 7.2.2.1]
WP_008140971.1	K02115	ATPF1G, atpG; F-type H+-transporting ATPase subunit gamma
WP_008140998.1	K21063	K21063; 5-amino-6-(5-phospho-D-ribitylamino)uracil phosphatase [EC:3.1.3.104]
WP_008140999.1	K06180	rluD; 23S rRNA pseudouridine1911/1915/1917 synthase [EC:5.4.99.23]
WP_008141002.1	K01006	ppdK; pyruvate, orthophosphate dikinase [EC:2.7.9.1]
WP_008141011.1	K00428	E1.11.1.5; cytochrome c peroxidase [EC:1.11.1.5]
WP_008141029.1	K00783	rlmH; 23S rRNA (pseudouridine1915-N3)-methyltransferase [EC:2.1.1.177]
WP_008141030.1	K00767	nadC, QPRT; nicotinate-nucleotide pyrophosphorylase (carboxylating) [EC:2.4.2.19]
WP_008141032.1	K03657	uvrD, pcrA; ATP-dependent DNA helicase UvrD/PcrA [EC:5.6.2.4]
WP_008141035.1	K00793	ribE, RIB5; riboflavin synthase [EC:2.5.1.9]
WP_008141036.1	K07058	K07058; membrane protein

WP_008141045.1	K03322	mntH; manganese transport protein
WP_008141054.1	K06020	ettA; energy-dependent translational throttle protein EttA
WP_008141092.1	K03924	moxR; MoxR-like ATPase [EC:3.6.3.-]
WP_008141110.1	K26937	dinF, mepA, vmrA; MATE family, multidrug efflux pump
WP_008141113.1	K03106	SRP54, ffh; signal recognition particle subunit SRP54 [EC:3.6.5.4]
WP_008141117.1	K06950	K06950; uncharacterized protein
WP_008141120.1	K03648	UNG, UDG; uracil-DNA glycosylase [EC:3.2.2.27]
WP_008141122.1	K01914	asnA; aspartate--ammonia ligase [EC:6.3.1.1]
WP_008141145.1	K06201	cutC; copper homeostasis protein
WP_008141148.1	K01192	E3.2.1.25, MANBA, manB; beta-mannosidase [EC:3.2.1.25]
WP_008141155.1	K01187	malZ; alpha-glucosidase [EC:3.2.1.20]
WP_008141164.1	K02564	nagB, GNPDA; glucosamine-6-phosphate deaminase [EC:3.5.99.6]
WP_008141165.1	K02564	nagB, GNPDA; glucosamine-6-phosphate deaminase [EC:3.5.99.6]
WP_008141182.1	K01810	GPI, pgi; glucose-6-phosphate isomerase [EC:5.3.1.9]
WP_008141185.1	K00057	gpsA; glycerol-3-phosphate dehydrogenase (NAD(P)+) [EC:1.1.1.94]
WP_008141187.1	K04567	KARS, lysS; lysyl-tRNA synthetase, class II [EC:6.1.1.6]
WP_008141193.1	K00180	iorB; indolepyruvate ferredoxin oxidoreductase, beta subunit [EC:1.2.7.8]
WP_008141196.1	K01912	paaK; phenylacetate-CoA ligase [EC:6.2.1.30]
WP_008141198.1	K15987	hppA; K(+)-stimulated pyrophosphate-energized sodium pump [EC:7.2.3.1]
WP_008141200.1	K27802	acnA; aconitate hydratase A / 2-methylisocitrate dehydratase [EC:4.2.1.3 4.2.1.99]
WP_008141262.1	K06173	truA, PUS1; tRNA pseudouridine38-40 synthase [EC:5.4.99.12]
WP_008141270.1	K02477	K02477; two-component system, LytTR family, response regulator
WP_008141273.1	K07095	K07095; uncharacterized protein
WP_008141282.1	K07089	K07089; uncharacterized protein
WP_008141333.1	K03737	por, nifJ; pyruvate-ferredoxin/flavodoxin oxidoreductase [EC:1.2.7.1 1.2.8.1]
WP_008141338.1	K03770	ppiD; peptidyl-prolyl cis-trans isomerase D [EC:5.2.1.8]
WP_008141339.1	K03699	tlyC; magnesium and cobalt exporter, CNNM family
WP_008141348.1	K27956	lnb; lipoprotein N-acyltransferase [EC:2.3.1.-]
WP_008141352.1	K03070	secA; preprotein translocase subunit SecA [EC:7.4.2.8]
WP_008141379.1	K03100	lepB; signal peptidase I [EC:3.4.21.89]

WP_008141393.1	K01873	VARS, valS; valyl-tRNA synthetase [EC:6.1.1.9]
WP_008141401.1	K21572	susD; starch-binding outer membrane protein, SusD/RagB family
WP_008141404.1	K12373	HEXA_B; hexosaminidase [EC:3.2.1.52]
WP_008141410.1	K03088	rpoE; RNA polymerase sigma-70 factor, ECF subfamily
WP_008141425.1	K04041	fbp3; fructose-1,6-bisphosphatase III [EC:3.1.3.11]
WP_008141465.1	K01579	panD; aspartate 1-decarboxylase [EC:4.1.1.11]
WP_008141470.1	K07405	E3.2.1.1A; alpha-amylase [EC:3.2.1.1]
WP_008141473.1	K01875	SARS, serS; seryl-tRNA synthetase [EC:6.1.1.11]
WP_008141475.1	K01627	kdsA; 2-dehydro-3-deoxyphosphooctonate aldolase (KDO 8-P synthase) [EC:2.5.1.55]
WP_008141477.1	K00791	miaA, TRIT1; tRNA dimethylallyltransferase [EC:2.5.1.75]
WP_008141481.1	K05801	djlA; DnaJ like chaperone protein
WP_008141494.1	K07804	pagC; putatice virulence related protein PagC
WP_008141500.1	K00688	PYG, glgP; glycogen phosphorylase [EC:2.4.1.1]
WP_008141506.1	K02124	ATPVK, ntpK, atpK; V/A-type H+/Na+-transporting ATPase subunit K
WP_008141508.1	K02123	ATPVI, ntpI, atpI; V/A-type H+/Na+-transporting ATPase subunit I
WP_008141510.1	K02120	ATPVD, ntpD, atpD; V/A-type H+/Na+-transporting ATPase subunit D
WP_008141512.1	K02118	ATPVB, ntpB, atpB; V/A-type H+/Na+-transporting ATPase subunit B
WP_008141513.1	K02117	ATPVA, ntpA, atpA; V/A-type H+/Na+-transporting ATPase subunit A [EC:7.1.2.2 7.2.2.1]
WP_008141517.1	K02121	ATPVE, ntpE, atpE; V/A-type H+/Na+-transporting ATPase subunit E
WP_008141527.1	K07713	zraR, hydG; two-component system, NtrC family, response regulator HydG
WP_008141550.1	K03169	topB; DNA topoisomerase III [EC:5.6.2.1]
WP_008141565.1	K07735	algH; putative transcriptional regulator
WP_008141566.1	K00657	speG, SAT; diamine N-acetyltransferase [EC:2.3.1.57]
WP_008141569.1	K03978	engB; GTP-binding protein
WP_008141575.1	K12373	HEXA_B; hexosaminidase [EC:3.2.1.52]
WP_008141585.1	K16089	TC.FEV.OM2, cirA, cfrA, hmuR; outer membrane receptor for ferrienterochelin and colicins
WP_008141588.1	K02230	cobN; cobaltochelatase CobN [EC:6.6.1.2]
WP_008141600.1	K03437	spoU; RNA methyltransferase, TrmH family
WP_008141605.1	K03101	lspA; signal peptidase II [EC:3.4.23.36]
WP_008141612.1	K01870	IARS, ileS; isoleucyl-tRNA synthetase [EC:6.1.1.5]
WP_008141617.1	K01890	FARSB, pheT; phenylalanyl-tRNA synthetase beta chain [EC:6.1.1.20]

WP_008141623.1	K00919	ispE; 4-diphosphocytidyl-2-C-methyl-D-erythritol kinase [EC:2.7.1.148]
WP_008141625.1	K06177	rluA; tRNA pseudouridine32 synthase / 23S rRNA pseudouridine746 synthase [EC:5.4.99.28 5.4.99.29]
WP_008141631.1	K03305	TC.POT; proton-dependent oligopeptide transporter, POT family
WP_008141632.1	K03305	TC.POT; proton-dependent oligopeptide transporter, POT family
WP_008141635.1	K00053	ilvC; ketol-acid reductoisomerase [EC:1.1.1.86]
WP_008141637.1	K01653	E2.2.1.6S, ilvH, ilvN; acetolactate synthase I/III small subunit [EC:2.2.1.6]
WP_008141639.1	K01652	E2.2.1.6L, ilvB, ilvG, ilvI; acetolactate synthase I/II/III large subunit [EC:2.2.1.6]
WP_008141649.1	K09797	K09797; uncharacterized protein
WP_008141650.1	K02945	RP-S1, rpsA; small subunit ribosomal protein S1
WP_008141681.1	K01270	pepD; dipeptidase D [EC:3.4.13.-]
WP_008141685.1	K03534	rhaM; L-rhamnose mutarotase [EC:5.1.3.32]
WP_008141686.1	K03671	TXN, trxA; thioredoxin
WP_008141687.1	K18691	mltF; peptidoglycan lytic transglycosylase F [EC:4.2.2.29]
WP_008141689.1	K00876	udk, UCK; uridine kinase [EC:2.7.1.48]
WP_008141692.1	K00765	hisG; ATP phosphoribosyltransferase [EC:2.4.2.17]
WP_008141693.1	K00013	hisD; histidinol dehydrogenase [EC:1.1.1.23]
WP_008141694.1	K00817	hisC; histidinol-phosphate aminotransferase [EC:2.6.1.9]
WP_008141695.1	K01089	hisB; imidazoleglycerol-phosphate dehydratase / histidinol-phosphatase [EC:4.2.1.19 3.1.3.15]
WP_008141698.1	K09702	K09702; uncharacterized protein
WP_008141701.1	K18139	oprM, emhC, ttgC, cusC, adeK, smeF, mtrE, cmeC, gesC; outer membrane protein, multidrug efflux system
WP_008141702.1	K18138	acrB, mexB, adeJ, smeE, mtrD, cmeB; multidrug efflux pump
WP_008141703.1	K03585	acrA, mexA, adeI, smeD, mtrC, cmeA; membrane fusion protein, multidrug efflux system
WP_008141706.1	K12340	tolC, bepC, cyaE, raxC, sapF, rsaF, hasF; outer membrane protein
WP_008141710.1	K03585	acrA, mexA, adeI, smeD, mtrC, cmeA; membrane fusion protein, multidrug efflux system
WP_008141711.1	K08289	purT; phosphoribosylglycinamide formyltransferase 2 [EC:6.3.1.21]
WP_008141713.1	K10947	K10947; PadR family transcriptional regulator
WP_008141719.1	K01139	spoT; GTP diphosphokinase / guanosine-3',5'-bis(diphosphate) 3'-diphosphatase [EC:2.7.6.5 3.1.7.2]

WP_008141724.1	K02959	RP-S16, MRPS16, rpsP; small subunit ribosomal protein S16
WP_008141731.1	K02501	hisH; imidazole glycerol-phosphate synthase subunit HisH [EC:4.3.2.10]
WP_008141732.1	K01814	hisA; phosphoribosylformimino-5-aminoimidazole carboxamide ribotide isomerase [EC:5.3.1.16]
WP_008141733.1	K02500	hisF; imidazole glycerol-phosphate synthase subunit HisF [EC:4.3.2.10]
WP_008141735.1	K11755	hisIE; phosphoribosyl-AMP cyclohydrolase / phosphoribosyl-ATP pyrophosphohydrolase [EC:3.5.4.19 3.6.1.31]
WP_008141737.1	K00928	lysC; aspartate kinase [EC:2.7.2.4]
WP_008141738.1	K01586	lysA; diaminopimelate decarboxylase [EC:4.1.1.20]
WP_008141741.1	K21556	malR; CRP/FNR family transcriptional regulator, polysaccharide utilization system transcription regulator
WP_008141742.1	K09808	lolC_E_F; lipoprotein-releasing system permease protein
WP_008141743.1	K00812	aspB; aspartate aminotransferase [EC:2.6.1.1]
WP_008141747.1	K06997	yggS, PROSC; PLP dependent protein
WP_008141748.1	K00226	pyrD; dihydroorotate dehydrogenase (fumarate) [EC:1.3.98.1]
WP_008141751.1	K01897	ACSL, fadD; long-chain-fatty-acid---CoA ligase [EC:6.2.1.3]
WP_008141766.1	K10206	E2.6.1.83; LL-diaminopimelate aminotransferase [EC:2.6.1.83]
WP_008141787.1	K01190	lacZ; beta-galactosidase [EC:3.2.1.23]
WP_008141800.1	K06180	rluD; 23S rRNA pseudouridine1911/1915/1917 synthase [EC:5.4.99.23]
WP_008141802.1	K02356	efp; elongation factor P
WP_008141808.1	K03088	rpoE; RNA polymerase sigma-70 factor, ECF subfamily
WP_008141810.1	K03630	radC; DNA repair protein RadC
WP_008141816.1	K02470	gyrB; DNA gyrase subunit B [EC:5.6.2.2]
WP_008141817.1	K02968	RP-S20, rpsT; small subunit ribosomal protein S20
WP_008141821.1	K01992	ABC-2.P; ABC-2 type transport system permease protein
WP_008141822.1	K01990	ABC-2.A; ABC-2 type transport system ATP-binding protein
WP_008141824.1	K09117	K09117; uncharacterized protein
WP_008141826.1	K03531	ftsZ; cell division protein FtsZ
WP_008141827.1	K03590	ftsA; cell division protein FtsA
WP_008141828.1	K03589	ftsQ; cell division protein FtsQ
WP_008141829.1	K01924	murC; UDP-N-acetylmuramate--alanine ligase [EC:6.3.2.8]

WP_008141830.1	K02563	murG; UDP-N-acetylglucosamine--N-acetylmuramyl-(pentapeptide) pyrophosphoryl-undecaprenol N-acetylglucosamine transferase [EC:2.4.1.227]
WP_008141831.1	K03588	ftsW, spoVE, rodA; peptidoglycan glycosyltransferase [EC:2.4.99.28]
WP_008141832.1	K01925	murD; UDP-N-acetylmuramoylalanine--D-glutamate ligase [EC:6.3.2.9]
WP_008141833.1	K01000	mraY; phospho-N-acetylmuramoyl-pentapeptide-transferase [EC:2.7.8.13]
WP_008141834.1	K01928	murE; UDP-N-acetylmuramoyl-L-alanyl-D-glutamate--2,6-diaminopimelate ligase [EC:6.3.2.13]
WP_008141835.1	K03587	ftsI; cell division protein FtsI (penicillin-binding protein 3) [EC:3.4.16.4]
WP_008141837.1	K03438	mraW, rsmH; 16S rRNA (cytosine1402-N4)-methyltransferase [EC:2.1.1.199]
WP_008141871.1	K00946	thiL; thiamine-monophosphate kinase [EC:2.7.4.16]
WP_008141873.1	K03783	punA, PNP; purine-nucleoside phosphorylase [EC:2.4.2.1]
WP_008141875.1	K00912	lpxK; tetraacyldisaccharide 4'-kinase [EC:2.7.1.130]
WP_008141877.1	K04773	sppA; protease IV [EC:3.4.21.-]
WP_008141910.1	K08281	pncA; nicotinamidase/pyrazinamidase [EC:3.5.1.19 3.5.1.-]
WP_008141912.1	K00763	pncB, NAPRT1; nicotinate phosphoribosyltransferase [EC:6.3.4.21]
WP_008141927.1	K26937	dinF, mepA, vmrA; MATE family, multidrug efflux pump
WP_008141933.1	K03116	tatA; sec-independent protein translocase protein TatA
WP_008141939.1	K18139	oprM, emhC, ttgC, cusC, adeK, smeF, mtrE, cmeC, gesC; outer membrane protein, multidrug efflux system
WP_008141940.1	K18138	acrB, mexB, adeJ, smeE, mtrD, cmeB; multidrug efflux pump
WP_008141952.1	K09825	perR; Fur family transcriptional regulator, peroxide stress response regulator
WP_008141954.1	K01950	E6.3.5.1, NADSYN1, QNS1, nadE; NAD+ synthase (glutamine-hydrolysing) [EC:6.3.5.1]
WP_008141956.1	K03426	NUDT12_13, nudC; NAD+ diphosphatase [EC:3.6.1.22]
WP_008141957.1	K06959	tex; protein Tex
WP_008141959.1	K24217	mnaT; L-amino acid N-acyltransferase [EC:2.3.1.-]
WP_008141963.1	K05516	cbpA; curved DNA-binding protein
WP_008142095.1	K03497	parB, spo0J; ParB family transcriptional regulator, chromosome partitioning protein
WP_008142107.1	K01835	pgm; phosphoglucomutase [EC:5.4.2.2]
WP_008142109.1	K02039	phoU; phosphate transport system protein

WP_008142110.1	K02036	pstB; phosphate transport system ATP-binding protein [EC:7.3.2.1]
WP_008142111.1	K02038	pstA; phosphate transport system permease protein
WP_008142112.1	K02037	pstC; phosphate transport system permease protein
WP_008142113.1	K02040	pstS; phosphate transport system substrate-binding protein
WP_008142114.1	K06889	K06889; uncharacterized protein
WP_008142116.1	K03183	ubiE; demethylmenaquinone methyltransferase / 2-methoxy-6-polyprenyl-1,4-benzoquinol methylase [EC:2.1.1.163 2.1.1.201]
WP_008142118.1	K06217	phoH, phoL; phosphate starvation-inducible protein PhoH and related proteins
WP_008142123.1	K12373	HEXA_B; hexosaminidase [EC:3.2.1.52]
WP_008142135.1	K00901	dgkA, DGK; diacylglycerol kinase (ATP) [EC:2.7.1.107]
WP_008142136.1	K01142	xthA, xth; exodeoxyribonuclease III [EC:3.1.11.2]
WP_008142140.1	K03701	uvrA; excinuclease ABC subunit A
WP_008142142.1	K00254	DHODH, pyrD; dihydroorotate dehydrogenase [EC:1.3.5.2]
WP_008142145.1	K03547	sbcD, mre11; DNA repair protein SbcD/Mre11
WP_008142149.1	K00788	thiE; thiamine-phosphate pyrophosphorylase [EC:2.5.1.3]
WP_008142151.1	K03150	thiH; 2-iminoacetate synthase [EC:4.1.99.19]
WP_008142152.1	K03147	thiC; phosphomethylpyrimidine synthase [EC:4.1.99.17]
WP_008142153.1	K03149	thiG; thiazole synthase [EC:2.8.1.10]
WP_008142154.1	K00788	thiE; thiamine-phosphate pyrophosphorylase [EC:2.5.1.3]
WP_008142155.1	K03154	thiS; sulfur carrier protein
WP_008142159.1	K03839	fldA, nifF, isiB; flavodoxin I
WP_008142161.1	K09790	K09790; uncharacterized protein
WP_008142163.1	K02619	pabC; 4-amino-4-deoxychorismate lyase [EC:4.1.3.38]
WP_008142164.1	K00868	pdxK, pdxY; pyridoxine kinase [EC:2.7.1.35]
WP_008142167.1	K00831	serC, PSAT1; phosphoserine aminotransferase [EC:2.6.1.52]
WP_008142168.1	K00058	serA, PHGDH; D-3-phosphoglycerate dehydrogenase / 2-oxoglutarate reductase [EC:1.1.1.95 1.1.1.399]
WP_008142175.1	K18369	adh2; alcohol dehydrogenase [EC:1.1.1.-]
WP_008142180.1	K11921	cynR; LysR family transcriptional regulator, cyn operon transcriptional activator
WP_008142182.1	K03781	katE, CAT, catB, srpA; catalase [EC:1.11.1.6]
WP_008142275.1	K07484	K07484; transposase
WP_008142278.1	K07484	K07484; transposase
WP_008142293.1	K07114	yfbK; Ca-activated chloride channel homolog

WP_008142295.1	K07114	yfbK; Ca-activated chloride channel homolog
WP_008142301.1	K03924	moxR; MoxR-like ATPase [EC:3.6.3.-]
WP_008142305.1	K03530	hupB; DNA-binding protein HU-beta
WP_008142307.1	K14441	rimO; ribosomal protein S12 methylthiotransferase [EC:2.8.4.4]
WP_008142309.1	K03110	ftsY; fused signal recognition particle receptor
WP_008142313.1	K02902	RP-L28, MRPL28, rpmB; large subunit ribosomal protein L28
WP_008142316.1	K25706	tsaD; tRNA N6-adenosine threonylcarbamoyltransferase [EC:2.3.1.234]
WP_008142318.1	K01265	map; methionyl aminopeptidase [EC:3.4.11.18]
WP_008142330.1	K00654	SPT, spt; serine palmitoyltransferase [EC:2.3.1.50]
WP_008142333.1	K15460	yfiC, trmX; tRNA1Val (adenine37-N6)-methyltransferase [EC:2.1.1.223]
WP_008142336.1	K11720	lptG; lipopolysaccharide export system permease protein
WP_008142349.1	K01079	serB, PSPH; phosphoserine phosphatase [EC:3.1.3.3]
WP_008142356.1	K00012	UGDH, ugd; UDPglucose 6-dehydrogenase [EC:1.1.1.22]
WP_008142362.1	K02231	cobP, cobU; adenosylcobinamide kinase / adenosylcobinamide-phosphate guanylyltransferase [EC:2.7.1.156 2.7.7.62]
WP_008142363.1	K00768	E2.4.2.21, cobU, cobT; nicotinate-nucleotide--dimethylbenzimidazole phosphoribosyltransferase [EC:2.4.2.21]
WP_008142372.1	K03150	thiH; 2-iminoacetate synthase [EC:4.1.99.19]
WP_008142405.1	K04720	cobD; threonine-phosphate decarboxylase [EC:4.1.1.81]
WP_008142410.1	K00940	ndk, NME; nucleoside-diphosphate kinase [EC:2.7.4.6]
WP_008142584.1	K11749	rseP; regulator of sigma E protease [EC:3.4.24.-]
WP_008142585.1	K00099	dxr; 1-deoxy-D-xylulose-5-phosphate reductoisomerase [EC:1.1.1.267]
WP_008142590.1	K02860	rimM; 16S rRNA processing protein RimM
WP_008142592.1	K00790	murA; UDP-N-acetylglucosamine 1-carboxyvinyltransferase [EC:2.5.1.7]
WP_008142599.1	K00942	gmk, GUK1; guanylate kinase [EC:2.7.4.8]
WP_008142603.1	K00969	nadD; nicotinate-nucleotide adenylyltransferase [EC:2.7.7.18]
WP_008142609.1	K07079	K07079; uncharacterized protein
WP_008142614.1	K01770	ispF; 2-C-methyl-D-erythritol 2,4-cyclodiphosphate synthase [EC:4.6.1.12]
WP_008142617.1	K01926	rex; redox-sensing transcriptional repressor
WP_008142623.1	K18303	mexK; multidrug efflux pump
WP_008142630.1	K12343	SRD5A1; 3-oxo-5-alpha-steroid 4-dehydrogenase 1 [EC:1.3.1.22]

WP_008142631.1	K22347	nox; 4,4'-dithiodibutanoate disulfide reductase [EC:1.8.1.20]
WP_008142633.1	K01673	cynT, can; carbonic anhydrase [EC:4.2.1.1]
WP_008142634.1	K00602	purH; phosphoribosylaminoimidazolecarboxamide formyltransferase / IMP cyclohydrolase [EC:2.1.2.3 3.5.4.10]
WP_008142644.1	K02967	RP-S2, MRPS2, rpsB; small subunit ribosomal protein S2
WP_008142645.1	K02996	RP-S9, MRPS9, rpsI; small subunit ribosomal protein S9
WP_008142655.1	K01893	NARS, asnS; asparaginyl-tRNA synthetase [EC:6.1.1.22]
WP_008142661.1	K01205	NAGLU; alpha-N-acetylglucosaminidase [EC:3.2.1.50]
WP_008142665.1	K21572	susD; starch-binding outer membrane protein, SusD/RagB family
WP_008142691.1	K16345	xanP; xanthine permease XanP
WP_008142692.1	K03816	xpt; xanthine phosphoribosyltransferase [EC:2.4.2.22]
WP_008142699.1	K03308	TC.NSS; neurotransmitter:Na+ symporter, NSS family
WP_008142701.1	K03088	rpoE; RNA polymerase sigma-70 factor, ECF subfamily
WP_008142704.1	K02836	prfB; peptide chain release factor 2
WP_008142705.1	K01897	ACSL, fadD; long-chain-fatty-acid---CoA ligase [EC:6.2.1.3]
WP_008142706.1	K01438	argE; acetylornithine deacetylase [EC:3.5.1.16]
WP_008142709.1	K22719	envC; murein hydrolase activator
WP_008142713.1	K01520	dut, DUT; dUTP diphosphatase [EC:3.6.1.23]
WP_008142721.1	K01163	K01163; uncharacterized protein
WP_008142725.1	K06518	cidA; holin-like protein
WP_008142726.1	K00625	pta; phosphate acetyltransferase [EC:2.3.1.8]
WP_008142728.1	K00925	ackA; acetate kinase [EC:2.7.2.1]
WP_008142746.1	K21575	susA; neopullulanase [EC:3.2.1.135]
WP_008142748.1	K21574	susB; glucan 1,4-alpha-glucosidase [EC:3.2.1.3]
WP_008142750.1	K21573	susC; TonB-dependent starch-binding outer membrane protein SusC
WP_008142751.1	K21572	susD; starch-binding outer membrane protein, SusD/RagB family
WP_008142758.1	K15633	gpmI; 2,3-bisphosphoglycerate-independent phosphoglycerate mutase [EC:5.4.2.12]
WP_008142763.1	K03470	rnhB; ribonuclease HII [EC:3.1.26.4]
WP_008142764.1	K11717	sufS; cysteine desulfurase / selenocysteine lyase [EC:2.8.1.7 4.4.1.16]
WP_008142766.1	K09015	sufD; Fe-S cluster assembly protein SufD
WP_008142768.1	K09013	sufC; Fe-S cluster assembly ATP-binding protein
WP_008142770.1	K09014	sufB; Fe-S cluster assembly protein SufB
WP_008142775.1	K02519	infB, MTIF2; translation initiation factor IF-2

WP_008142777.1	K02600	nusA; transcription termination/antitermination protein NusA
WP_008142778.1	K09748	rimP; ribosome maturation factor RimP
WP_008142786.1	K12373	HEXA_B; hexosaminidase [EC:3.2.1.52]
WP_008142798.1	K03088	rpoE; RNA polymerase sigma-70 factor, ECF subfamily
WP_008142799.1	K00930	argB; acetylglutamate kinase [EC:2.7.2.8]
WP_008142801.1	K01585	speA; arginine decarboxylase [EC:4.1.1.19]
WP_008142803.1	K00891	aroK, aroL; shikimate kinase [EC:2.7.1.71]
WP_008142805.1	K03168	topA; DNA topoisomerase I [EC:5.6.2.1]
WP_008142815.1	K03718	asnC; Lrp/AsnC family transcriptional regulator, regulator for asnA, asnC and gidA
WP_008142823.1	K00001	E1.1.1.1, adh; alcohol dehydrogenase [EC:1.1.1.1]
WP_008142831.1	K07107	ybgC; acyl-CoA thioester hydrolase [EC:3.1.2.-]
WP_008142837.1	K12410	cobB, srtN, npdA; NAD-dependent protein deacetylase/lipoamidase [EC:2.3.1.286 2.3.1.313]
WP_008142841.1	K03324	yjbB; phosphate:Na+ symporter
WP_008142843.1	K00876	udk, UCK; uridine kinase [EC:2.7.1.48]
WP_008142845.1	K03092	rpoN; RNA polymerase sigma-54 factor
WP_008142849.1	K02437	gcvH, GCSH; glycine cleavage system H protein
WP_008142851.1	K01588	purE; 5-(carboxyamino)imidazole ribonucleotide mutase [EC:5.4.99.18]
WP_008142853.1	K03526	gcpE, ispG; (E)-4-hydroxy-3-methylbut-2-enyl-diphosphate synthase [EC:1.17.7.1 1.17.7.3]
WP_008142855.1	K03797	E3.4.21.102, prc, ctpA; carboxyl-terminal processing protease [EC:3.4.21.102]
WP_008142859.1	K03284	corA; magnesium transporter
WP_008142862.1	K01776	murI; glutamate racemase [EC:5.1.1.3]
WP_008142864.1	K06142	hlpA, ompH; outer membrane protein
WP_008142865.1	K07277	SAM50, TOB55, bamA; outer membrane protein insertion porin family
WP_008142866.1	K00806	uppS; undecaprenyl diphosphate synthase [EC:2.5.1.31]
WP_008142869.1	K02493	hemK, prmC, HEMK; release factor glutamine methyltransferase [EC:2.1.1.297]
WP_008142870.1	K03565	recX; regulatory protein
WP_008142872.1	K00762	pyrE; orotate phosphoribosyltransferase [EC:2.4.2.10]
WP_008142874.1	K01755	argH, ASL; argininosuccinate lyase [EC:4.3.2.1]
WP_008142888.1	K12373	HEXA_B; hexosaminidase [EC:3.2.1.52]
WP_008142889.1	K09808	lolC_E_F; lipoprotein-releasing system permease protein
WP_008142890.1	K02834	rbfA; ribosome-binding factor A
WP_008142894.1	K00873	PK, pyk; pyruvate kinase [EC:2.7.1.40]
WP_008142895.1	K03786	aroQ, qutE; 3-dehydroquinate dehydratase II [EC:4.2.1.10]
WP_008142896.1	K04763	xerD; integrase/recombinase XerD

WP_008142902.1	K03530	hupB; DNA-binding protein HU-beta
WP_008142967.1	K05810	LACC1, yfiH; purine-nucleoside/S-methyl-5'-thioadenosine phosphorylase / adenosine deaminase [EC:2.4.2.1 2.4.2.28 3.5.4.4]
WP_008142969.1	K00939	adk, AK; adenylate kinase [EC:2.7.4.3]
WP_008142970.1	K00760	hprT, hpt, HPRT1; hypoxanthine phosphoribosyltransferase [EC:2.4.2.8]
WP_008142971.1	K23997	nnr; ADP-dependent NAD(P)H-hydrate dehydratase / NAD(P)H-hydrate epimerase [EC:4.2.1.136 5.1.99.6]
WP_008142977.1	K01624	FBA, fbaA; fructose-bisphosphate aldolase, class II [EC:4.1.2.13]
WP_008142983.1	K03655	recG; ATP-dependent DNA helicase RecG [EC:5.6.2.4]
WP_008142985.1	K00991	ispD; 2-C-methyl-D-erythritol 4-phosphate cytidylyltransferase [EC:2.7.7.60]
WP_008142986.1	K03152	thiJ; protein deglycase [EC:3.5.1.124]
WP_008142990.1	K03559	exbD; biopolymer transport protein ExbD
WP_008142992.1	K03561	exbB; biopolymer transport protein ExbB
WP_008142993.1	K03474	pdxJ; pyridoxine 5-phosphate synthase [EC:2.6.99.2]
WP_008142996.1	K00858	ppnK, NADK; NAD+ kinase [EC:2.7.1.23]
WP_008143073.1	K03469	rnhA, RNASEH1; ribonuclease HI [EC:3.1.26.4]
WP_008143078.1	K07391	comM; magnesium chelatase family protein
WP_008143079.1	K02003	ABC.CD.A; putative ABC transport system ATP-binding protein
WP_008143085.1	K02005	ABC.CD.TX; HlyD family secretion protein
WP_008143112.1	K01874	MARS, metG; methionyl-tRNA synthetase [EC:6.1.1.10]
WP_008143119.1	K07271	licD; lipopolysaccharide cholinephosphotransferase [EC:2.7.8.-]
WP_008143141.1	K03543	emrA; membrane fusion protein, multidrug efflux system
WP_008143151.1	K01243	mtnN, mtn, pfs; adenosylhomocysteine nucleosidase [EC:3.2.2.9]
WP_008143152.1	K07173	luxS; S-ribosylhomocysteine lyase [EC:4.4.1.21]
WP_008143158.1	K01206	FUCA; alpha-L-fucosidase [EC:3.2.1.51]
WP_008143160.1	K02956	RP-S15, MRPS15, rpsO; small subunit ribosomal protein S15
WP_008143164.1	K07148	K07148; uncharacterized protein
WP_008143168.1	K06207	typA, bipA; GTP-binding protein
WP_008143169.1	K20534	gtrB; polyisoprenyl-phosphate glycosyltransferase [EC:2.4.-.-]
WP_008143179.1	K02517	lpxL, htrB; Kdo2-lipid IVA lauroyltransferase/acyltransferase [EC:2.3.1.241 2.3.1.-]
WP_008143181.1	K11085	msbA; ATP-binding cassette, subfamily B, bacterial MsbA [EC:7.5.2.6]

WP_008143188.1	K03473	pdxB; erythronate-4-phosphate dehydrogenase [EC:1.1.1.290]
WP_008143190.1	K01206	FUCA; alpha-L-fucosidase [EC:3.2.1.51]
WP_008143191.1	K05349	bglX; beta-glucosidase [EC:3.2.1.21]
WP_008143201.1	K23514	fecI, prhI; RNA polymerase sigma-19 factor, ECF subfamily
WP_008143209.1	K21572	susD; starch-binding outer membrane protein, SusD/RagB family
WP_008143225.1	K09458	fabF, OXSM, CEM1; 3-oxoacyl-[acyl-carrier-protein] synthase II [EC:2.3.1.179]
WP_008143238.1	K03685	rnc, DROSHA, RNT1; ribonuclease III [EC:3.1.26.3]
WP_008143240.1	K21071	pfk, pfp; ATP-dependent phosphofructokinase / diphosphate-dependent phosphofructokinase [EC:2.7.1.11 2.7.1.90]
WP_008143253.1	K27493	exoMA2; D-arabinan exo beta-(1,2)-arabinofuranosidase (non-reducing end) [EC:3.2.1.224]
WP_008143264.1	K01883	CARS, cysS; cysteinyl-tRNA synthetase [EC:6.1.1.16]
WP_008143269.1	K01012	bioB; biotin synthase [EC:2.8.1.6]
WP_008143270.1	K00833	bioA; adenosylmethionine---8-amino-7-oxononanoate aminotransferase [EC:2.6.1.62]
WP_008143271.1	K00652	bioF; 8-amino-7-oxononanoate synthase [EC:2.3.1.47]
WP_008143272.1	K09789	bioG; pimeloyl-[acyl-carrier protein] methyl ester esterase [EC:3.1.1.85]
WP_008143273.1	K02169	bioC; malonyl-CoA O-methyltransferase [EC:2.1.1.197]
WP_008143275.1	K01935	bioD; dethiobiotin synthetase [EC:6.3.3.3]
WP_008143283.1	K05540	dusB; tRNA-dihydrouridine synthase B [EC:1.-.-.-]
WP_008143288.1	K03073	secE; preprotein translocase subunit SecE
WP_008143291.1	K21572	susD; starch-binding outer membrane protein, SusD/RagB family
WP_008143309.1	K00640	cysE; serine O-acetyltransferase [EC:2.3.1.30]
WP_008143316.1	K01206	FUCA; alpha-L-fucosidase [EC:3.2.1.51]
WP_008143321.1	K25137	rlmL; 23S rRNA (guanine2445-N2)-methyltransferase [EC:2.1.1.173]
WP_008143322.1	K01278	DPP4, CD26; dipeptidyl-peptidase 4 [EC:3.4.14.5]
WP_008143324.1	K01945	purD; phosphoribosylamine---glycine ligase [EC:6.3.4.13]
WP_008143336.1	K09815	znuA; zinc transport system substrate-binding protein
WP_008143338.1	K09817	znuC; zinc transport system ATP-binding protein [EC:7.2.2.20]
WP_008143363.1	K00382	DLD, lpd, pdhD; dihydrolipoyl dehydrogenase [EC:1.8.1.4]
WP_008143367.1	K00278	nadB; L-aspartate oxidase [EC:1.4.3.16]
WP_008143388.1	K00826	E2.6.1.42, ilvE; branched-chain amino acid aminotransferase [EC:2.6.1.42]

WP_008143390.1	K03602	xseB; exodeoxyribonuclease VII small subunit [EC:3.1.11.6]
WP_008143391.1	K03601	xseA; exodeoxyribonuclease VII large subunit [EC:3.1.11.6]
WP_008143397.1	K00937	ppk1; polyphosphate kinase [EC:2.7.4.1]
WP_008143398.1	K07095	K07095; uncharacterized protein
WP_008143403.1	K19294	algI; alginate O-acetyltransferase complex protein AlgI
WP_008143413.1	K18139	oprM, emhC, ttgC, cusC, adeK, smeF, mtrE, cmeC, gesC; outer membrane protein, multidrug efflux system
WP_008143414.1	K18138	acrB, mexB, adeJ, smeE, mtrD, cmeB; multidrug efflux pump
WP_008143416.1	K03585	acrA, mexA, adeI, smeD, mtrC, cmeA; membrane fusion protein, multidrug efflux system
WP_008143417.1	K00029	maeB; malate dehydrogenase (oxaloacetate-decarboxylating)(NADP+) [EC:1.1.1.40]
WP_008143420.1	K00262	E1.4.1.4, gdhA; glutamate dehydrogenase (NADP+) [EC:1.4.1.4]
WP_008143423.1	K09810	lolD; lipoprotein-releasing system ATP-binding protein [EC:7.6.2.-]
WP_008143425.1	K22132	tcdA; tRNA threonylcarbamoyladenosine dehydratase
WP_008143428.1	K26730	napA, nhaS3, nhaS5, gerN; Na+:H+ antiporter
WP_008143430.1	K00133	asd; aspartate-semialdehyde dehydrogenase [EC:1.2.1.11]
WP_008143434.1	K01206	FUCA; alpha-L-fucosidase [EC:3.2.1.51]
WP_008143438.1	K01487	guaD, GDA; guanine deaminase [EC:3.5.4.3]
WP_008143442.1	K04077	groEL, HSPD1, HSP60, CPN60; chaperonin GroEL [EC:5.6.1.7]
WP_008143444.1	K04078	groES, HSPE1; chaperonin GroES
WP_008143449.1	K09760	rmuC; DNA recombination protein RmuC
WP_008143453.1	K01524	ppx-gppA; exopolyphosphatase / guanosine-5'-triphosphate,3'-diphosphate pyrophosphatase [EC:3.6.1.11 3.6.1.40]
WP_008143456.1	K07012	cas3; CRISPR-associated endonuclease/helicase Cas3 [EC:3.1.-.- 5.6.2.4]
WP_008143458.1	K19119	cas5d; CRISPR-associated protein Cas5d
WP_008143459.1	K19117	csd1, cas8c; CRISPR-associated protein Csd1
WP_008143461.1	K19118	csd2, cas7; CRISPR-associated protein Csd2
WP_008143462.1	K07464	cas4; CRISPR-associated exonuclease Cas4 [EC:3.1.12.1]
WP_008143464.1	K15342	cas1; CRISP-associated protein Cas1
WP_008143466.1	K09951	cas2; CRISPR-associated protein Cas2
WP_008143471.1	K07491	rayT; REP-associated tyrosine transposase
WP_008143486.1	K01892	HARS, hisS; histidyl-tRNA synthetase [EC:6.1.1.21]
WP_008143487.1	K06973	K06973; uncharacterized protein

WP_008143492.1	K03169	topB; DNA topoisomerase III [EC:5.6.2.1]
WP_008143497.1	K00560	thyA, TYMS; thymidylate synthase [EC:2.1.1.45]
WP_008143498.1	K00287	DHFR, folA; dihydrofolate reductase [EC:1.5.1.3]
WP_008143507.1	K03561	exbB; biopolymer transport protein ExbB
WP_008143508.1	K03424	tatD; TatD DNase family protein [EC:3.1.21.-]
WP_008143510.1	K03832	tonB; periplasmic protein TonB
WP_008143517.1	K03527	ispH, lytB; 4-hydroxy-3-methylbut-2-en-1-yl diphosphate reductase [EC:1.17.7.4]
WP_008143518.1	K00850	pfkA, PFK; 6-phosphofructokinase 1 [EC:2.7.1.11]
WP_008143524.1	K11069	potD; spermidine/putrescine transport system substrate-binding protein
WP_008143525.1	K11070	potC; spermidine/putrescine transport system permease protein
WP_008143526.1	K11071	potB; spermidine/putrescine transport system permease protein
WP_008143527.1	K11072	potA; spermidine/putrescine transport system ATP-binding protein [EC:7.6.2.11]
WP_008143529.1	K07588	MMAA, argK; GTPase [EC:3.6.5.-]
WP_008143540.1	K01270	pepD; dipeptidase D [EC:3.4.13.-]
WP_008143542.1	K02528	ksgA; 16S rRNA (adenine1518-N6/adenine1519-N6)-dimethyltransferase [EC:2.1.1.182]
WP_008143543.1	K06213	mgtE; magnesium transporter
WP_008143545.1	K01798	alr-murF; Alr-MurF fusion protein [EC:5.1.1.1 6.3.2.10]
WP_008143551.1	K03499	trkA, ktrA, ktrC; trk/ktr system potassium uptake protein
WP_008143554.1	K00567	ogt, MGMT; methylated-DNA-[protein]-cysteine S-methyltransferase [EC:2.1.1.63]
WP_008143566.1	K08316	rsmD; 16S rRNA (guanine966-N2)-methyltransferase [EC:2.1.1.171]
WP_008143567.1	K06131	clsA_B; cardiolipin synthase A/B [EC:2.7.8.-]
WP_008143570.1	K01735	aroB; 3-dehydroquinate synthase [EC:4.2.3.4]
WP_008143571.1	K03699	tlyC; magnesium and cobalt exporter, CNNM family
WP_008143578.1	K09816	znuB; zinc transport system permease protein
WP_008143591.1	K03671	TXN, trxA; thioredoxin
WP_008143592.1	K02337	dnaE; DNA polymerase III subunit alpha [EC:2.7.7.7]
WP_008143596.1	K11991	tadA; tRNA(adenine34) deaminase [EC:3.5.4.33]
WP_008143599.1	K07460	yraN; putative endonuclease
WP_008143600.1	K03524	birA; BirA family transcriptional regulator, biotin operon repressor / biotin---[acetyl-CoA-carboxylase] ligase [EC:6.3.4.15]
WP_008143601.1	K26937	dinF, mepA, vmrA; MATE family, multidrug efflux pump
WP_008143602.1	K09903	pyrH; uridylate kinase [EC:2.7.4.22]
WP_008143607.1	K02838	frr, MRRF, RRF; ribosome recycling factor

WP_008143608.1	K06949	rsgA, engC; ribosome biogenesis GTPase / thiamine phosphate phosphatase [EC:3.6.1.- 3.1.3.100]
WP_008143610.1	K03585	acrA, mexA, adeI, smeD, mtrC, cmeA; membrane fusion protein, multidrug efflux system
WP_008143611.1	K18138	acrB, mexB, adeJ, smeE, mtrD, cmeB; multidrug efflux pump
WP_008143612.1	K12340	tolC, bepC, cyaE, raxC, sapF, rsaF, hasF; outer membrane protein
WP_008143613.1	K03294	TC.APA; basic amino acid/polyamine antiporter, APA family
WP_008143614.1	K01082	cysQ, MET22, BPNT1; 3'(2'), 5'-bisphosphate nucleotidase [EC:3.1.3.7]
WP_008143617.1	K00860	cysC; adenylylsulfate kinase [EC:2.7.1.25]
WP_008143619.1	K00957	cysD; sulfate adenylyltransferase subunit 2 [EC:2.7.7.4]
WP_008143620.1	K00956	cysN; sulfate adenylyltransferase subunit 1 [EC:2.7.7.4]
WP_008143623.1	K01206	FUCA; alpha-L-fucosidase [EC:3.2.1.51]
WP_008143624.1	K03453	TC.BASS; bile acid:Na+ symporter, BASS family
WP_008143628.1	K01639	E4.1.3.3, nanA, NPL; N-acetylneuraminate lyase [EC:4.1.3.3]
WP_008143629.1	K01787	RENBP; N-acylglucosamine 2-epimerase [EC:5.1.3.8]
WP_008143632.1	K01186	NEU1; sialidase-1 [EC:3.2.1.18]
WP_008143634.1	K07407	E3.2.1.22B, galA, rafA; alpha-galactosidase [EC:3.2.1.22]
WP_008143641.1	K00024	mdh; malate dehydrogenase [EC:1.1.1.37]
WP_008143647.1	K02316	dnaG; DNA primase [EC:2.7.7.101]
WP_008143649.1	K01495	GCH1, folE; GTP cyclohydrolase IA [EC:3.5.4.16]
WP_008143665.1	K00847	E2.7.1.4, scrK; fructokinase [EC:2.7.1.4]
WP_008143667.1	K03332	fruA; fructan beta-fructosidase [EC:3.2.1.80]
WP_008143670.1	K03340	dapdh; diaminopimelate dehydrogenase [EC:1.4.1.16]
WP_008143671.1	K03550	ruvA; holliday junction DNA helicase RuvA
WP_008143672.1	K04564	SOD2; superoxide dismutase, Fe-Mn family [EC:1.15.1.1]
WP_008143674.1	K00974	cca; tRNA nucleotidyltransferase (CCA-adding enzyme) [EC:2.7.7.72 3.1.3.- 3.1.4.-]
WP_008143679.1	K21636	nrdD; ribonucleoside-triphosphate reductase (formate) [EC:1.1.98.6]
WP_008143681.1	K04068	nrdG; anaerobic ribonucleoside-triphosphate reductase activating protein [EC:1.97.1.4]
WP_008143719.1	K25026	glk; glucokinase [EC:2.7.1.2]
WP_008143737.1	K07322	ytfE, scdA; regulator of cell morphogenesis and NO signaling
WP_008143739.1	K05366	mrcA; penicillin-binding protein 1A [EC:2.4.99.28 3.4.16.4]

WP_008143743.1	K00979	kdsB; 3-deoxy-manno-octulosonate cytidylyltransferase (CMP-KDO synthetase) [EC:2.7.7.38]
WP_008143746.1	K01092	E3.1.3.25, IMPA, suhB; myo-inositol-1(or 4)-monophosphatase [EC:3.1.3.25]
WP_008143748.1	K00700	GBE1, glgB; 1,4-alpha-glucan branching enzyme [EC:2.4.1.18]
WP_008143771.1	K00784	rnz; ribonuclease Z [EC:3.1.26.11]
WP_008143782.1	K07164	K07164; uncharacterized protein
WP_008143786.1	K01206	FUCA; alpha-L-fucosidase [EC:3.2.1.51]
WP_008143787.1	K02529	galR; LacI family transcriptional regulator, galactose operon repressor
WP_008143791.1	K01241	amn; AMP nucleosidase [EC:3.2.2.4]
WP_008143792.1	K02340	holA; DNA polymerase III subunit delta [EC:2.7.7.7]
WP_008143795.1	K02823	pyrDII; dihydroorotate dehydrogenase electron transfer subunit
WP_008143796.1	K17828	pyrDI; dihydroorotate dehydrogenase (NAD+) catalytic subunit [EC:1.3.1.14]
WP_008143797.1	K00554	trmD; tRNA (guanine37-N1)-methyltransferase [EC:2.1.1.228]
WP_008143798.1	K01972	E6.5.1.2, ligA, ligB; DNA ligase (NAD+) [EC:6.5.1.2]
WP_008143804.1	K07001	K07001; NTE family protein
WP_008143813.1	K04079	HSP90A, htpG; molecular chaperone HtpG
WP_008143814.1	K03696	clpC; ATP-dependent Clp protease ATP-binding subunit ClpC
WP_008143816.1	K02469	gyrA; DNA gyrase subunit A [EC:5.6.2.2]
WP_008143832.1	K03798	ftsH, hflB; cell division protease FtsH [EC:3.4.24.-]
WP_008143834.1	K00981	E2.7.7.41, CDS1, CDS2, cdsA; phosphatidate cytidylyltransferase [EC:2.7.7.41]
WP_008143835.1	K00748	lpxB; lipid-A-disaccharide synthase [EC:2.4.1.182]
WP_008143837.1	K03787	surE; 5'/3'-nucleotidase [EC:3.1.3.5 3.1.3.6]
WP_008143838.1	K03496	parA, soj; chromosome partitioning protein
WP_008143841.1	K03497	parB, spo0J; ParB family transcriptional regulator, chromosome partitioning protein
WP_008143844.1	K08307	mltD, dniR; peptidoglycan lytic transglycosylase D [EC:4.2.2.29]
WP_008143848.1	K01139	spoT; GTP diphosphokinase / guanosine-3',5'-bis(diphosphate) 3'-diphosphatase [EC:2.7.6.5 3.1.7.2]
WP_008143853.1	K01872	AARS, alaS; alanyl-tRNA synthetase [EC:6.1.1.7]
WP_008143862.1	K00602	purH; phosphoribosylaminoimidazolecarboxamide formyltransferase / IMP cyclohydrolase [EC:2.1.2.3 3.5.4.10]
WP_008143864.1	K03569	mreB; rod shape-determining protein MreB and related proteins
WP_008143866.1	K03570	mreC; rod shape-determining protein MreC

WP_008143869.1	K05515	mrdA; penicillin-binding protein 2 [EC:3.4.16.4]
WP_008143871.1	K05837	rodA, mrdB; peptidoglycan glycosyltransferase [EC:2.4.99.28]
WP_008143878.1	K02341	holB; DNA polymerase III subunit delta' [EC:2.7.7.7]
WP_008143880.1	K00297	metF, MTHFR; methylenetetrahydrofolate reductase (NADH) [EC:1.5.1.54]
WP_008143894.1	K07404	pgl; 6-phosphogluconolactonase [EC:3.1.1.31]
WP_008143906.1	K05970	SIAE; sialate O-acetylesterase [EC:3.1.1.53]
WP_008143908.1	K01676	E4.2.1.2A, fumA, fumB; fumarate hydratase, class I [EC:4.2.1.2]
WP_008143911.1	K03665	hflX; GTPase
WP_008143917.1	K21572	susD; starch-binding outer membrane protein, SusD/RagB family
WP_008143931.1	K07056	rsmI; 16S rRNA (cytidine1402-2'-O)-methyltransferase [EC:2.1.1.198]
WP_008143933.1	K00857	tdk, TK; thymidine kinase [EC:2.7.1.21]
WP_008143950.1	K26939	norM, mdtK; MATE family, multidrug efflux pump
WP_008143954.1	K07713	zraR, hydG; two-component system, NtrC family, response regulator HydG
WP_008143959.1	K06180	rluD; 23S rRNA pseudouridine1911/1915/1917 synthase [EC:5.4.99.23]
WP_008143960.1	K00059	fabG, OAR1; 3-oxoacyl-[acyl-carrier protein] reductase [EC:1.1.1.100]
WP_008143970.1	K01955	carB, CPA2; carbamoyl-phosphate synthase large subunit [EC:6.3.5.5]
WP_008143976.1	K09861	K09861; uncharacterized protein
WP_008143977.1	K03572	mutL; DNA mismatch repair protein MutL
WP_008143981.1	K03771	surA; peptidyl-prolyl cis-trans isomerase SurA [EC:5.2.1.8]
WP_008143986.1	K00088	IMPDH, guaB; IMP dehydrogenase [EC:1.1.1.205]
WP_008143988.1	K03654	recQ; ATP-dependent DNA helicase RecQ [EC:5.6.2.4]
WP_008143991.1	K01358	clpP, CLPP; ATP-dependent Clp protease, protease subunit [EC:3.4.21.92]
WP_008143993.1	K03545	tig; trigger factor
WP_008143995.1	K06861	lptB; lipopolysaccharide export system ATP-binding protein [EC:7.5.2.5]
WP_008143998.1	K02066	mlaE, linK; phospholipid/cholesterol/gamma-HCH transport system permease protein
WP_008143999.1	K02065	mlaF, linL, mkl; phospholipid/cholesterol/gamma-HCH transport system ATP-binding protein
WP_008144001.1	K03977	engA, der; GTPase
WP_008144004.1	K03595	era, ERAL1; GTPase
WP_008144009.1	K01443	nagA, AMDHD2; N-acetylglucosamine-6-phosphate deacetylase [EC:3.5.1.25]

WP_008144010.1	K01443	nagA, AMDHD2; N-acetylglucosamine-6-phosphate deacetylase [EC:3.5.1.25]
WP_008144028.1	K03654	recQ; ATP-dependent DNA helicase RecQ [EC:5.6.2.4]
WP_008144030.1	K07462	recJ; single-stranded-DNA-specific exonuclease [EC:3.1.-.-]
WP_008144037.1	K18682	rny; ribonucrease Y [EC:3.1.-.-]
WP_008144043.1	K01737	queD, ptpS, PTS; 6-pyruvoyltetrahydropterin/6-carboxytetrahydropterin synthase [EC:4.2.3.12 4.1.2.50]
WP_008144045.1	K10026	queE; 7-carboxy-7-deazaguanine synthase [EC:4.3.99.3]
WP_008144051.1	K03310	TC.AGCS; alanine or glycine:cation symporter, AGCS family
WP_008144066.1	K01889	FARSA, pheS; phenylalanyl-tRNA synthetase alpha chain [EC:6.1.1.20]
WP_008144070.1	K00075	murB; UDP-N-acetylmuramate dehydrogenase [EC:1.3.1.98]
WP_008144074.1	K10773	NTHL1, nth; endonuclease III [EC:3.2.2.- 4.2.99.18]
WP_008144075.1	K03284	corA; magnesium transporter
WP_008144087.1	K06920	queC; 7-cyano-7-deazaguanine synthase [EC:6.3.4.20]
WP_008144092.1	K13052	divIC, divA; cell division protein DivIC
WP_008144103.1	K00655	plsC; 1-acyl-sn-glycerol-3-phosphate acyltransferase [EC:2.3.1.51]
WP_008144105.1	K07098	K07098; uncharacterized protein
WP_008144108.1	K00721	DPM1; dolichol-phosphate mannosyltransferase [EC:2.4.1.83]
WP_008144110.1	K00343	nuoN; NADH-quinone oxidoreductase subunit N [EC:7.1.1.2]
WP_008144111.1	K00342	nuoM; NADH-quinone oxidoreductase subunit M [EC:7.1.1.2]
WP_008144112.1	K00341	nuoL; NADH-quinone oxidoreductase subunit L [EC:7.1.1.2]
WP_008144113.1	K00340	nuoK; NADH-quinone oxidoreductase subunit K [EC:7.1.1.2]
WP_008144114.1	K00339	nuoJ; NADH-quinone oxidoreductase subunit J [EC:7.1.1.2]
WP_008144115.1	K00338	nuoI; NADH-quinone oxidoreductase subunit I [EC:7.1.1.2]
WP_008144116.1	K00337	nuoH; NADH-quinone oxidoreductase subunit H [EC:7.1.1.2]
WP_008144119.1	K13378	nuoCD; NADH-quinone oxidoreductase subunit C/D [EC:7.1.1.2]
WP_008144121.1	K00331	nuoB; NADH-quinone oxidoreductase subunit B [EC:7.1.1.2]

WP_008144123.1	K00330	nuoA; NADH-quinone oxidoreductase subunit A [EC:7.1.1.2]
WP_008144130.1	K03316	nhaP, nhaS1, nhaS2; Na+:H+ antiporter
WP_008144131.1	K01911	menE; o-succinylbenzoate---CoA ligase [EC:6.2.1.26]
WP_008144135.1	K01661	menB; naphthoate synthase [EC:4.1.3.36]
WP_008144137.1	K02551	menD; 2-succinyl-5-enolpyruvyl-6-hydroxy-3-cyclohexene-1-carboxylate synthase [EC:2.2.1.9]
WP_008144138.1	K02361	entC; isochorismate synthase [EC:5.4.4.2]
WP_008144140.1	K19222	menI, DHNAT; 1,4-dihydroxy-2-naphthoyl-CoA hydrolase [EC:3.1.2.28]
WP_008144142.1	K01206	FUCA; alpha-L-fucosidase [EC:3.2.1.51]
WP_008144144.1	K21572	susD; starch-binding outer membrane protein, SusD/RagB family
WP_008144154.1	K01740	metY; O-acetylhomoserine (thiol)-lyase [EC:2.5.1.49]
WP_008144163.1	K04516	AROA1, aroA; chorismate mutase [EC:5.4.99.5]
WP_008144165.1	K10206	E2.6.1.83; LL-diaminopimelate aminotransferase [EC:2.6.1.83]
WP_008144167.1	K04518	pheA2; prephenate dehydratase [EC:4.2.1.51]
WP_008144191.1	K06975	K06975; uncharacterized protein
WP_008144199.1	K07387	K07387; metalloprotease [EC:3.4.24.-]
WP_008144221.1	K03664	smpB; SsrA-binding protein
WP_008144223.1	K00548	metH, MTR; 5-methyltetrahydrofolate--homocysteine methyltransferase [EC:2.1.1.13]
WP_008144232.1	K03701	uvrA; excinuclease ABC subunit A
WP_008144236.1	K01515	nudF; ADP-ribose diphosphatase [EC:3.6.1.13 3.6.1.-]
WP_008144258.1	K01808	rpiB; ribose 5-phosphate isomerase B [EC:5.3.1.6]
WP_008144260.1	K00615	E2.2.1.1, tktA, tktB; transketolase [EC:2.2.1.1]
WP_008144263.1	K01953	asnB, ASNS; asparagine synthase (glutamine-hydrolysing) [EC:6.3.5.4]
WP_008144265.1	K01778	dapF; diaminopimelate epimerase [EC:5.1.1.7]
WP_008144267.1	K10206	E2.6.1.83; LL-diaminopimelate aminotransferase [EC:2.6.1.83]
WP_008144269.1	K01915	glnA, GLUL; glutamine synthetase [EC:6.3.1.2]
WP_008144275.1	K07456	mutS2; DNA mismatch repair protein MutS2
WP_008144281.1	K01091	gph; phosphoglycolate phosphatase [EC:3.1.3.18]
WP_008144282.1	K02888	RP-L21, MRPL21, rplU; large subunit ribosomal protein L21
WP_008144285.1	K02899	RP-L27, MRPL27, rpmA; large subunit ribosomal protein L27
WP_008144286.1	K07082	mltG; peptidoglycan lytic transglycosylase G [EC:4.2.2.29]
WP_008144288.1	K06001	trpB; tryptophan synthase beta chain [EC:4.2.1.20]
WP_008144290.1	K00764	purF, PPAT; amidophosphoribosyltransferase [EC:2.4.2.14]

WP_008144310.1	K00605	gcvT, AMT; glycine cleavage system T protein (aminomethyltransferase) [EC:2.1.2.10]
WP_008144313.1	K04762	hslR; ribosome-associated heat shock protein Hsp15
WP_008144315.1	K01056	PTH1, PTRH1, pth, spoVC; peptidyl-tRNA hydrolase, PTH1 family [EC:3.1.1.29]
WP_008144317.1	K02897	RP-L25, rplY; large subunit ribosomal protein L25
WP_008144320.1	K03625	nusB; transcription antitermination protein NusB
WP_008144322.1	K03210	yajC; preprotein translocase subunit YajC
WP_008144325.1	K00859	coaE; dephospho-CoA kinase [EC:2.7.1.24]
WP_008144331.1	K03695	clpB; ATP-dependent Clp protease ATP-binding subunit ClpB
WP_008144348.1	K00895	pfp, PFP; diphosphate-dependent phosphofructokinase [EC:2.7.1.90]
WP_008144350.1	K07273	acm; lysozyme
WP_008144353.1	K02687	prmA; ribosomal protein L11 methyltransferase [EC:2.1.1.-]
WP_008144369.1	K00177	korC, oorC; 2-oxoglutarate ferredoxin oxidoreductase subunit gamma [EC:1.2.7.3]
WP_008144374.1	K00174	korA, oorA, oforA; 2-oxoglutarate/2-oxoacid ferredoxin oxidoreductase subunit alpha [EC:1.2.7.3 1.2.7.11]
WP_008144376.1	K00176	korD, oorD; 2-oxoglutarate ferredoxin oxidoreductase subunit delta [EC:1.2.7.3]
WP_008144381.1	K01424	E3.5.1.1, ansA, ansB; L-asparaginase [EC:3.5.1.1]
WP_008144383.1	K01695	trpA; tryptophan synthase alpha chain [EC:4.2.1.20]
WP_008144385.1	K01817	trpF; phosphoribosylanthranilate isomerase [EC:5.3.1.24]
WP_008144387.1	K01609	trpC; indole-3-glycerol phosphate synthase [EC:4.1.1.48]
WP_008144389.1	K00766	trpD; anthranilate phosphoribosyltransferase [EC:2.4.2.18]
WP_008144391.1	K01658	trpG; anthranilate synthase component II [EC:4.1.3.27]
WP_008144396.1	K01696	trpB; tryptophan synthase beta chain [EC:4.2.1.20]
WP_008144407.1	K06872	K06872; uncharacterized protein
WP_008144409.1	K03744	lemA; LemA protein
WP_008144410.1	K06188	aqpZ, aqpM, AQP9; aquaporin
WP_008144416.1	K01961	accC; acetyl-CoA carboxylase, biotin carboxylase subunit [EC:6.4.1.2 6.3.4.14]
WP_008144418.1	K01966	PCCB, pccB; propionyl-CoA carboxylase beta subunit [EC:6.4.1.3 2.1.3.15]
WP_008144420.1	K01448	amiABC; N-acetylmuramoyl-L-alanine amidase [EC:3.5.1.28]
WP_008144422.1	K01206	FUCA; alpha-L-fucosidase [EC:3.2.1.51]
WP_008144424.1	K15923	AXY8, FUC95A, afcA; alpha-L-fucosidase 2 [EC:3.2.1.51]

WP_008144425.1	K03553	recA; recombination protein RecA
WP_008144426.1	K03564	BCP, PRXQ, DOT5; thioredoxin-dependent peroxiredoxin [EC:1.11.1.24]
WP_008144428.1	K27502	capadh; carboxyaminopropylagmatine dehydrogenase [EC:1.5.1.55]
WP_008144443.1	K04043	dnaK, HSPA9; molecular chaperone DnaK
WP_008144450.1	K00561	ermC, ermA; 23S rRNA (adenine-N6)-dimethyltransferase [EC:2.1.1.184]
WP_008144467.1	K00764	purF, PPAT; amidophosphoribosyltransferase [EC:2.4.2.14]
WP_008144468.1	K01956	carA, CPA1; carbamoyl-phosphate synthase small subunit [EC:6.3.5.5]
WP_008144469.1	K01955	carB, CPA2; carbamoyl-phosphate synthase large subunit [EC:6.3.5.5]
WP_008144471.1	K15342	cas1; CRISP-associated protein Cas1
WP_008144477.1	K01744	aspA; aspartate ammonia-lyase [EC:4.3.1.1]
WP_008144480.1	K03711	fur, zur, furB; Fur family transcriptional regulator, ferric uptake regulator
WP_008144482.1	K08301	rng, cafA; ribonuclease G [EC:3.1.26.-]
WP_008144483.1	K03530	hupB; DNA-binding protein HU-beta
WP_008144484.1	K03575	mutY; A/G-specific adenine glycosylase [EC:3.2.2.31]
WP_008144485.1	K03111	ssb; single-strand DNA-binding protein
WP_008144489.1	K00266	gltD; glutamate synthase (NADPH) small chain [EC:1.4.1.13]
WP_008144505.1	K15865	CDKAL1, mtaB; threonylcarbamoyladenosine tRNA methylthiotransferase [EC:2.8.4.5]
WP_008144509.1	K00791	miaA, TRIT1; tRNA dimethylallyltransferase [EC:2.5.1.75]
WP_008144514.1	K16363	lpxC-fabZ; UDP-3-O-[3-hydroxymyristoyl] N-acetylglucosamine deacetylase / 3-hydroxyacyl-[acyl-carrier-protein] dehydratase [EC:3.5.1.108 4.2.1.59]
WP_008144516.1	K02536	lpxD; UDP-3-O-[3-hydroxymyristoyl] glucosamine N-acyltransferase [EC:2.3.1.191]
WP_008144519.1	K01591	pyrF; orotidine-5'-phosphate decarboxylase [EC:4.1.1.23]
WP_008144520.1	K02835	prfA, MTRF1, MRF1; peptide chain release factor 1
WP_008144535.1	K00850	pfkA, PFK; 6-phosphofructokinase 1 [EC:2.7.1.11]
WP_008144547.1	K26937	dinF, mepA, vmrA; MATE family, multidrug efflux pump
WP_008144559.1	K02171	blaI; BlaI family transcriptional regulator, penicillinase repressor
WP_008144570.1	K00927	PGK, pgk; phosphoglycerate kinase [EC:2.7.2.3]
WP_008144571.1	K06076	fadL; long-chain fatty acid transport protein
WP_008144576.1	K01190	lacZ; beta-galactosidase [EC:3.2.1.23]

WP_008144586.1	K01599	hemE, UROD; uroporphyrinogen decarboxylase [EC:4.1.1.37]
WP_008144590.1	K03307	TC.SSS; solute:Na+ symporter, SSS family
WP_008144607.1	K16692	etk-wzc; tyrosine-protein kinase Etk/Wzc [EC:2.7.10.3]
WP_008144609.1	K01991	wza, gfcE; polysaccharide biosynthesis/export protein
WP_008144659.1	K02621	parC; topoisomerase IV subunit A [EC:5.6.2.2]
WP_008144662.1	K26937	dinF, mepA, vmrA; MATE family, multidrug efflux pump
WP_008144664.1	K01880	GARS, glyS1; glycyl-tRNA synthetase [EC:6.1.1.14]
WP_008144668.1	K03773	fklB; FKBP-type peptidyl-prolyl cis-trans isomerase FklB [EC:5.2.1.8]
WP_008144669.1	K15726	czcA, cusA, cnrA; heavy metal efflux system protein
WP_008144672.1	K02477	K02477; two-component system, LytTR family, response regulator
WP_008144674.1	K15727	czcB, cusB, cnrB; membrane fusion protein, heavy metal efflux system
WP_008144675.1	K15726	czcA, cusA, cnrA; heavy metal efflux system protein
WP_008144680.1	K00651	metA; homoserine O-succinyltransferase/O-acetyltransferase [EC:2.3.1.46 2.3.1.31]
WP_008144682.1	K24847	rlhA; 23S rRNA 5-hydroxycytidine C2501 synthase
WP_008144697.1	K07240	chrA; chromate transporter
WP_008144699.1	K07240	chrA; chromate transporter
WP_008144731.1	K09167	K09167; uncharacterized protein
WP_008144742.1	K01895	ACSS1_2, acs; acetyl-CoA synthetase [EC:6.2.1.1]
WP_008144752.1	K03402	argR, ahrC; transcriptional regulator of arginine metabolism
WP_008144755.1	K01940	argG, ASS1; argininosuccinate synthase [EC:6.3.4.5]
WP_008144756.1	K00145	argC; N-acetyl-gamma-glutamyl-phosphate reductase [EC:1.2.1.38]
WP_008144758.1	K00821	argD; acetylornithine/N-succinyldiaminopimelate aminotransferase [EC:2.6.1.11 2.6.1.17]
WP_008144764.1	K01990	ABC-2.A; ABC-2 type transport system ATP-binding protein
WP_008144765.1	K03387	ahpF; NADH-dependent peroxiredoxin subunit F [EC:1.8.1.-]
WP_008144766.1	K24119	ahpC; NADH-dependent peroxiredoxin subunit C [EC:1.11.1.26]
WP_008144768.1	K08218	ampG; MFS transporter, PAT family, beta-lactamase induction signal transducer AmpG
WP_008144770.1	K18785	mp2; beta-1,4-mannooligosaccharide/beta-1,4-mannosyl-N-acetylglucosamine phosphorylase [EC:2.4.1.319 2.4.1.320]
WP_008144781.1	K04485	radA, sms; DNA repair protein RadA/Sms

WP_008144783.1	K12524	thrA; bifunctional aspartokinase / homoserine dehydrogenase 1 [EC:2.7.2.4 1.1.1.3]
WP_008144784.1	K15635	apgM; 2,3-bisphosphoglycerate-independent phosphoglycerate mutase [EC:5.4.2.12]
WP_008144785.1	K01733	thrC; threonine synthase [EC:4.2.3.1]
WP_008144786.1	K03439	trmB, METTL1, TRM8; tRNA (guanine-N7-)-methyltransferase [EC:2.1.1.33]
WP_008144787.1	K03593	mrp, NUBPL; ATP-binding protein involved in chromosome partitioning
WP_008144789.1	K01190	lacZ; beta-galactosidase [EC:3.2.1.23]
WP_008144790.1	K02346	dinB; DNA polymerase IV [EC:2.7.7.7]
WP_008144794.1	K03281	clcA, clcB, CLC-E, CLC-F; chloride channel protein, CIC family
WP_008144796.1	K00604	MTFMT, fmt; methionyl-tRNA formyltransferase [EC:2.1.2.9]
WP_008144798.1	K03088	rpoE; RNA polymerase sigma-70 factor, ECF subfamily
WP_008144803.1	K01783	rpe, RPE; ribulose-phosphate 3-epimerase [EC:5.1.3.1]
WP_008144805.1	K02238	comEC; competence protein ComEC
WP_008144814.1	K07139	K07139; uncharacterized protein
WP_008144820.1	K03442	mscS; small conductance mechanosensitive channel
WP_008144834.1	K06901	pbuG, azgA, ghxP, ghxQ, adeQ; adenine/guanine/hypoxanthine permease
WP_008144839.1	K01625	eda; 2-dehydro-3-deoxyphosphogluconate aldolase / (4S)-4-hydroxy-2-oxoglutarate aldolase [EC:4.1.2.14 4.1.3.42]
WP_008144841.1	K00874	kdgK; 2-dehydro-3-deoxygluconokinase [EC:2.7.1.45]
WP_008144842.1	K01815	kduI; 4-deoxy-L-threo-5-hexosulose-uronate ketol-isomerase [EC:5.3.1.17]
WP_008144843.1	K02775	gatC, sgcC; galactitol PTS system EIIC component
WP_008144851.1	K00046	idnO; gluconate 5-dehydrogenase [EC:1.1.1.69]
WP_008144872.1	K00931	proB; glutamate 5-kinase [EC:2.7.2.11]
WP_008144874.1	K00147	proA; glutamate-5-semialdehyde dehydrogenase [EC:1.2.1.41]
WP_008144876.1	K01847	MUT; methylmalonyl-CoA mutase [EC:5.4.99.2]
WP_008144879.1	K01847	MUT; methylmalonyl-CoA mutase [EC:5.4.99.2]
WP_008144883.1	K06158	ABCF3; ATP-binding cassette, subfamily F, member 3
WP_008144885.1	K01791	wecB; UDP-N-acetylglucosamine 2-epimerase (non-hydrolysing) [EC:5.1.3.14]
WP_008144887.1	K00849	galK; galactokinase [EC:2.7.1.6]
WP_008144890.1	K01785	galM, GALM; aldose 1-epimerase [EC:5.1.3.3]
WP_008144891.1	K11754	folC; dihydrofolate synthase / folylpolyglutamate synthase [EC:6.3.2.12 6.3.2.17]
WP_008144896.1	K21572	susD; starch-binding outer membrane protein, SusD/RagB family

WP_008144908.1	K01197	hya; hyaluronoglucosaminidase [EC:3.2.1.35]
WP_008144912.1	K02429	fucP, deoP, nagP, agaP; MFS transporter, FHS family, fucose-galactose-glucose:H+ symporter family protein
WP_008144915.1	K00848	rhaB; rhamnulokinase [EC:2.7.1.5]
WP_008144921.1	K00048	fucO; lactaldehyde reductase [EC:1.1.1.77]
WP_008144922.1	K01818	fucI; L-fucose/D-arabinose isomerase [EC:5.3.1.25 5.3.1.3]
WP_008144940.1	K03687	GRPE; molecular chaperone GrpE
WP_008144982.1	K06881	nrnA; bifunctional oligoribonuclease and PAP phosphatase NrnA [EC:3.1.3.7 3.1.13.3]
WP_008144985.1	K01840	manB; phosphomannomutase [EC:5.4.2.8]
WP_008144997.1	K02355	fusA, GFM, EFG; elongation factor G
WP_008144998.1	K07636	phoR; two-component system, OmpR family, phosphate regulon sensor histidine kinase PhoR [EC:2.7.13.3]
WP_008145004.1	K02990	RP-S6, MRPS6, rpsF; small subunit ribosomal protein S6
WP_008145006.1	K02939	RP-L9, MRPL9, rplI; large subunit ribosomal protein L9
WP_008145012.1	K00763	pncB, NAPRT1; nicotinate phosphoribosyltransferase [EC:6.3.4.21]
WP_008145018.1	K01649	leuA, IMS; 2-isopropylmalate synthase [EC:2.3.3.13]
WP_008145019.1	K01703	leuC, IPMI-L; 3-isopropylmalate/(R)-2-methylmalate dehydratase large subunit [EC:4.2.1.33 4.2.1.35]
WP_008145021.1	K01704	leuD, IPMI-S; 3-isopropylmalate/(R)-2-methylmalate dehydratase small subunit [EC:4.2.1.33 4.2.1.35]
WP_008145023.1	K09011	cimA; (R)-citramalate synthase [EC:2.3.3.21]
WP_008145027.1	K07304	msrA; peptide-methionine (S)-S-oxide reductase [EC:1.8.4.11]
WP_008145032.1	K03654	recQ; ATP-dependent DNA helicase RecQ [EC:5.6.2.4]
WP_008145044.1	K03711	fur, zur, furB; Fur family transcriptional regulator, ferric uptake regulator
WP_008145046.1	K01939	purA, ADSS; adenylosuccinate synthase [EC:6.3.4.4]
WP_008145048.1	K06158	ABCF3; ATP-binding cassette, subfamily F, member 3
WP_008145049.1	K07386	pepO; putative endopeptidase [EC:3.4.24.-]
WP_008145050.1	K07568	queA; S-adenosylmethionine:tRNA ribosyltransferase-isomerase [EC:2.4.99.17]
WP_008145053.1	K05306	phnX; phosphonoacetaldehyde hydrolase [EC:3.11.1.1]
WP_008145054.1	K03430	phnW; 2-aminoethylphosphonate-pyruvate transaminase [EC:2.6.1.37]
WP_008145058.1	K02614	paaI; acyl-CoA thioesterase [EC:3.1.2.-]
WP_008145060.1	K26937	dinF, mepA, vmrA; MATE family, multidrug efflux pump
WP_008145070.1	K07085	K07085; putative transport protein

WP_008145071.1	K16053	ybdG, mscM; miniconductance mechanosensitive channel
WP_008145074.1	K03521	fixA, etfB; electron transfer flavoprotein beta subunit
WP_008145075.1	K03522	fixB, etfA; electron transfer flavoprotein alpha subunit
WP_008145083.1	K03088	rpoE; RNA polymerase sigma-70 factor, ECF subfamily
WP_008145092.1	K11105	cvrA, nhaP2; potassium/hydrogen antiporter
WP_008145096.1	K25028	btuD; cobalamin transport system ATP-binding protein [EC:7.6.2.8]
WP_008145098.1	K25034	btuF; cobalamin transport system substrate-binding protein
WP_008145106.1	K12373	HEXA_B; hexosaminidase [EC:3.2.1.52]
WP_008145108.1	K12373	HEXA_B; hexosaminidase [EC:3.2.1.52]
WP_008145122.1	K23514	fecI, prhI; RNA polymerase sigma-19 factor, ECF subfamily
WP_008145124.1	K10716	kch, trkA, mthK, pch; voltage-gated potassium channel
WP_008145126.1	K18139	oprM, emhC, ttgC, cusC, adeK, smeF, mtrE, cmeC, gesC; outer membrane protein, multidrug efflux system
WP_008145127.1	K18138	acrB, mexB, adeJ, smeE, mtrD, cmeB; multidrug efflux pump
WP_008145128.1	K03585	acrA, mexA, adeI, smeD, mtrC, cmeA; membrane fusion protein, multidrug efflux system
WP_008145139.1	K00243	K00243; uncharacterized protein
WP_008145153.1	K12340	tolC, bepC, cyaE, raxC, sapF, rsaF, hasF; outer membrane protein
WP_008145154.1	K02005	ABC.CD.TX; HlyD family secretion protein
WP_008145155.1	K02004	ABC.CD.P; putative ABC transport system permease protein
WP_008145156.1	K02004	ABC.CD.P; putative ABC transport system permease protein
WP_008145159.1	K02003	ABC.CD.A; putative ABC transport system ATP-binding protein
WP_008145162.1	K25026	glk; glucokinase [EC:2.7.1.2]
WP_008145169.1	K02884	RP-L19, MRPL19, rplS; large subunit ribosomal protein L19
WP_008145177.1	K01738	cysK; cysteine synthase [EC:2.5.1.47]
WP_008145180.1	K00812	aspB; aspartate aminotransferase [EC:2.6.1.1]
WP_008145181.1	K06890	K06890; uncharacterized protein
WP_008145182.1	K14652	ribBA; 3,4-dihydroxy 2-butanone 4-phosphate synthase / GTP cyclohydrolase II [EC:4.1.99.12 3.5.4.25]
WP_008145183.1	K07091	lptF; lipopolysaccharide export system permease protein
WP_008145188.1	K01091	gph; phosphoglycolate phosphatase [EC:3.1.3.18]
WP_008145190.1	K01573	oadG; oxaloacetate decarboxylase (Na+ extruding) subunit gamma

WP_008145192.1	K01960	pycB; pyruvate carboxylase subunit B [EC:6.4.1.1]
WP_008145194.1	K20509	madB, oadB, gcdB, mmdB; carboxybiotin decarboxylase [EC:7.2.4.1]
WP_008145197.1	K00208	fabI; enoyl-[acyl-carrier protein] reductase I [EC:1.3.1.9 1.3.1.10]
WP_008145201.1	K07056	rsmI; 16S rRNA (cytidine1402-2'-O)-methyltransferase [EC:2.1.1.198]
WP_008145206.1	K01278	DPP4, CD26; dipeptidyl-peptidase 4 [EC:3.4.14.5]
WP_008145209.1	K01992	ABC-2.P; ABC-2 type transport system permease protein
WP_008145210.1	K01993	ABC-2.TX; HlyD family secretion protein
WP_008145211.1	K12340	tolC, bepC, cyaE, raxC, sapF, rsaF, hasF; outer membrane protein
WP_008145285.1	K23514	fecI, prhI; RNA polymerase sigma-19 factor, ECF subfamily
WP_008145297.1	K01190	lacZ; beta-galactosidase [EC:3.2.1.23]
WP_008145299.1	K01126	E3.1.4.46, glpQ, ugpQ; glycerophosphoryl diester phosphodiesterase [EC:3.1.4.46]
WP_008145303.1	K21572	susD; starch-binding outer membrane protein, SusD/RagB family
WP_008145305.1	K01206	FUCA; alpha-L-fucosidase [EC:3.2.1.51]
WP_008145394.1	K21572	susD; starch-binding outer membrane protein, SusD/RagB family
WP_008145402.1	K01190	lacZ; beta-galactosidase [EC:3.2.1.23]
WP_008145408.1	K00525	E1.17.4.1A, nrdA, nrdE; ribonucleoside-diphosphate reductase alpha chain [EC:1.17.4.1]
WP_008145415.1	K02067	mlaD, linM; phospholipid/cholesterol/gamma-HCH transport system substrate-binding protein
WP_008145419.1	K01491	folD; methylenetetrahydrofolate dehydrogenase (NADP+) / methenyltetrahydrofolate cyclohydrolase [EC:1.5.1.5 3.5.4.9]
WP_008145425.1	K00661	maa; maltose O-acetyltransferase [EC:2.3.1.79]
WP_008145426.1	K00100	bdhAB; butanol dehydrogenase [EC:1.1.1.-]
WP_008145427.1	K06889	K06889; uncharacterized protein
WP_008145449.1	K12373	HEXA_B; hexosaminidase [EC:3.2.1.52]
WP_008145451.1	K07148	K07148; uncharacterized protein
WP_008145461.1	K06287	yhdE; nucleoside triphosphate pyrophosphatase [EC:3.6.1.-]
WP_008145463.1	K03270	kdsC; 3-deoxy-D-manno-octulosonate 8-phosphate phosphatase (KDO 8-P phosphatase) [EC:3.1.3.45]
WP_008145472.1	K05606	MCEE, epi; methylmalonyl-CoA/ethylmalonyl-CoA epimerase [EC:5.1.99.1]
WP_008145473.1	K01604	mmdA; methylmalonyl-CoA decarboxylase subunit alpha [EC:7.2.4.3]

WP_008145475.1	K23351	gcdC, mmdC; glutaconyl-CoA/methylmalonyl-CoA decarboxylase subunit gamma
WP_008145476.1	K20509	madB, oadB, gcdB, mmdB; carboxybiotin decarboxylase [EC:7.2.4.1]
WP_008145658.1	K07484	K07484; transposase
WP_008659497.1	K03497	parB, spo0J; ParB family transcriptional regulator, chromosome partitioning protein
WP_008768657.1	K06921	K06921; uncharacterized protein
WP_022276647.1	K02961	RP-S17, MRPS17, rpsQ; small subunit ribosomal protein S17
WP_022276673.1	K00948	PRPS, prsA; ribose-phosphate pyrophosphokinase [EC:2.7.6.1]
WP_022276732.1	K01951	guaA, GMPS; GMP synthase (glutamine-hydrolysing) [EC:6.3.5.2]
WP_022276798.1	K13043	argF; N-succinyl-L-ornithine transcarbamylase [EC:2.1.3.11]
WP_022276811.1	K00286	proC; pyrroline-5-carboxylate reductase [EC:1.5.1.2]
WP_022276827.1	K03773	fklB; FKBP-type peptidyl-prolyl cis-trans isomerase FklB [EC:5.2.1.8]
WP_022276851.1	K03976	ybaK, ebsC; Cys-tRNA(Pro)/Cys-tRNA(Cys) deacylase [EC:3.1.1.-]
WP_022277062.1	K07040	yceD, ylbN; DUF177 domain-containing protein
WP_022277063.1	K00648	fabH; 3-oxoacyl-[acyl-carrier-protein] synthase III [EC:2.3.1.180]
WP_022277077.1	K01867	WARS, trpS; tryptophanyl-tRNA synthetase [EC:6.1.1.2]
WP_022277084.1	K01489	cdd, CDA; cytidine deaminase [EC:3.5.4.5]
WP_022277087.1	K02313	dnaA; chromosomal replication initiator protein
WP_022277096.1	K00262	E1.4.1.4, gdhA; glutamate dehydrogenase (NADP+) [EC:1.4.1.4]
WP_022277257.1	K11065	tpx; thioredoxin-dependent peroxiredoxin [EC:1.11.1.24]
WP_022277308.1	K00784	rnz; ribonuclease Z [EC:3.1.26.11]
WP_022277381.1	K01918	panC; pantoate--beta-alanine ligase [EC:6.3.2.1]
WP_022277383.1	K01915	glnA, GLUL; glutamine synthetase [EC:6.3.1.2]
WP_022277393.1	K09812	ftsE; cell division transport system ATP-binding protein
WP_022277427.1	K01868	TARS, thrS; threonyl-tRNA synthetase [EC:6.1.1.3]
WP_022277441.1	K02003	ABC.CD.A; putative ABC transport system ATP-binding protein
WP_022277464.1	K00782	lldG; L-lactate dehydrogenase complex protein LldG
WP_022277465.1	K18929	lldF; L-lactate dehydrogenase complex protein LldF
WP_022277470.1	K00425	cydA; cytochrome bd ubiquinol oxidase subunit I [EC:7.1.1.7]
WP_022277509.1	K07085	K07085; putative transport protein

WP_022277541.1	K01752	E4.3.1.17, sdaA, sdaB, tdcG; L-serine dehydratase [EC:4.3.1.17]
WP_022277558.1	K00175	korB, oorB, oforB; 2-oxoglutarate/2-oxoacid ferredoxin oxidoreductase subunit beta [EC:1.2.7.3 1.2.7.11]
WP_022277559.1	K02484	K02484; two-component system, OmpR family, sensor kinase [EC:2.7.13.3]
WP_022277567.1	K01736	aroC; chorismate synthase [EC:4.2.3.5]
WP_022277586.1	K01929	murF; UDP-N-acetylmuramoyl-tripeptide--D-alanyl-D-alanine ligase [EC:6.3.2.10]
WP_022277594.1	K00798	MMAB, pduO; cob(I)alamin adenosyltransferase [EC:2.5.1.17]
WP_022277611.1	K12373	HEXA_B; hexosaminidase [EC:3.2.1.52]
WP_022277617.1	K03076	secY; preprotein translocase subunit SecY
WP_022277629.1	K00800	aroA; 3-phosphoshikimate 1-carboxyvinyltransferase [EC:2.5.1.19]
WP_022277632.1	K06925	tsaE; tRNA threonylcarbamoyladenosine biosynthesis protein TsaE
WP_022277634.1	K17103	CHO1, pssA; CDP-diacylglycerol---serine O-phosphatidyltransferase [EC:2.7.8.8]
WP_022277665.1	K01803	TPI, tpiA; triosephosphate isomerase (TIM) [EC:5.3.1.1]
WP_022277674.1	K14742	tsaB; tRNA threonylcarbamoyladenosine biosynthesis protein TsaB
WP_022277710.1	K01710	rfbB, rmlB, rffG; dTDP-glucose 4,6-dehydratase [EC:4.2.1.46]
WP_022277754.1	K09125	yhhQ; queuosine precursor transporter
WP_022277765.1	K09888	zapA; cell division protein ZapA
WP_022277773.1	K01659	prpC; 2-methylcitrate synthase [EC:2.3.3.5]
WP_022277778.1	K00179	iorA; indolepyruvate ferredoxin oxidoreductase, alpha subunit [EC:1.2.7.8]
WP_022277818.1	K07560	dtd, DTD; D-aminoacyl-tRNA deacylase [EC:3.1.1.96]
WP_022277839.1	K03699	tlyC; magnesium and cobalt exporter, CNNM family
WP_022277858.1	K03719	lrp; Lrp/AsnC family transcriptional regulator, leucine-responsive regulatory protein
WP_022277882.1	K03644	lipA, LIAS, LIP1, LIP5; lipoyl synthase [EC:2.8.1.8]
WP_022277939.1	K00052	leuB, IMDH; 3-isopropylmalate dehydrogenase [EC:1.1.1.85]
WP_022277986.1	K02837	prfC; peptide chain release factor 3
WP_022278047.1	K01885	EARS, gltX; glutamyl-tRNA synthetase [EC:6.1.1.17]
WP_022278059.1	K01933	purM; phosphoribosylformylglycinamidine cyclo-ligase [EC:6.3.3.1]
WP_022278069.1	K06142	hlpA, ompH; outer membrane protein

WP_022278073.1	K11752	ribD; diaminohydroxyphosphoribosylaminopyrimidine deaminase / 5-amino-6-(5-phosphoribosylamino)uracil reductase [EC:3.5.4.26 1.1.1.193]
WP_022278112.1	K02357	tsf, TSFM; elongation factor Ts
WP_022278114.1	K02871	RP-L13, MRPL13, rplM; large subunit ribosomal protein L13
WP_022278115.1	K06178	rluB; 23S rRNA pseudouridine2605 synthase [EC:5.4.99.22]
WP_022278122.1	K23514	fecI, prhI; RNA polymerase sigma-19 factor, ECF subfamily
WP_022278146.1	K21557	susR; HTH-type transcriptional regulator, polysaccharide utilization system transcription regulator
WP_022278234.1	K03743	pncC; nicotinamide-nucleotide amidase [EC:3.5.1.42]
WP_022278244.1	K00773	tgt; queuine tRNA-ribosyltransferase [EC:2.4.2.29]
WP_022278280.1	K27684	capadc; carboxyaminopropylagmatine decarboxylase [EC:4.1.1.127]
WP_022278282.1	K03086	rpoD; RNA polymerase primary sigma factor
WP_022278284.1	K00606	panB; 3-methyl-2-oxobutanoate hydroxymethyltransferase [EC:2.1.2.11]
WP_022278312.1	K02377	TSTA3, fcl; GDP-L-fucose synthase [EC:1.1.1.271]
WP_040310995.1	K03498	trkH, trkG, ktrB, ktrD; trk/ktr system potassium uptake protein
WP_040311013.1	K03517	nadA; quinolinate synthase [EC:2.5.1.72]
WP_040311017.1	K00929	buk; butyrate kinase [EC:2.7.2.7]
WP_040311020.1	K08676	tri; tricorn protease [EC:3.4.21.-]
WP_040311021.1	K25767	nag31; exo-acting protein-alpha-N-acetylgalactosaminidase [EC:3.2.1.217]
WP_040311024.1	K02335	polA; DNA polymerase I [EC:2.7.7.7]
WP_040311025.1	K00215	dapB; 4-hydroxy-tetrahydrodipicolinate reductase [EC:1.17.1.8]
WP_040311028.1	K03088	rpoE; RNA polymerase sigma-70 factor, ECF subfamily
WP_040311030.1	K01537	ATP2C; P-type Ca2+ transporter type 2C [EC:7.2.2.10]
WP_040311031.1	K04066	priA; primosomal protein N' (replication factor Y) (superfamily II helicase) [EC:5.6.2.4]
WP_040311032.1	K25307	wzb, etp; low molecular weight protein-tyrosine phosphatase [EC:3.1.3.48]
WP_040311034.1	K03555	mutS; DNA mismatch repair protein MutS
WP_040311036.1	K03797	E3.4.21.102, prc, ctpA; carboxyl-terminal processing protease [EC:3.4.21.102]
WP_040311039.1	K07059	rho2; rhomboid family protease [EC:3.4.21.105]
WP_040311040.1	K28370	lysO; lysine exporter
WP_040311043.1	K00566	mnmA, trmU; tRNA-uridine 2-sulfurtransferase [EC:2.8.1.13]
WP_040311045.1	K04477	ycdX; putative hydrolase

WP_040311046.1	K07037	pgpH; cyclic-di-AMP phosphodiesterase PgpH [EC:3.1.4.-]
WP_040311047.1	K05808	hpf; ribosome hibernation promoting factor
WP_040311051.1	K02931	RP-L5, MRPL5, rplE; large subunit ribosomal protein L5
WP_040311055.1	K00348	nqrC; Na+-transporting NADH:ubiquinone oxidoreductase subunit C [EC:7.2.1.1]
WP_040311056.1	K08974	K08974; putative membrane protein
WP_040311057.1	K01886	QARS, glnS; glutaminyl-tRNA synthetase [EC:6.1.1.18]
WP_040311067.1	K02005	ABC.CD.TX; HlyD family secretion protein
WP_040311068.1	K07322	ytfE, scdA; regulator of cell morphogenesis and NO signaling
WP_040311069.1	K05595	marC; multiple antibiotic resistance protein
WP_040311074.1	K26937	dinF, mepA, vmrA; MATE family, multidrug efflux pump
WP_040311082.1	K06969	rlmI; 23S rRNA (cytosine1962-C5)-methyltransferase [EC:2.1.1.191]
WP_040311089.1	K02113	ATPF1D, atpH; F-type H+-transporting ATPase subunit delta
WP_040311097.1	K03315	nhaC; Na+:H+ antiporter, NhaC family
WP_040311101.1	K02429	fucP, deoP, nagP, agaP; MFS transporter, FHS family, fucose-galactose-glucose:H+ symporter family protein
WP_040311105.1	K02371	fabK; enoyl-[acyl-carrier protein] reductase II [EC:1.3.1.9]
WP_040311106.1	K02003	ABC.CD.A; putative ABC transport system ATP-binding protein
WP_040311118.1	K00865	glxK, garK; glycerate 2-kinase [EC:2.7.1.165]
WP_040311135.1	K01876	DARS2, aspS; aspartyl-tRNA synthetase [EC:6.1.1.12]
WP_040311143.1	K03215	rumA; 23S rRNA (uracil1939-C5)-methyltransferase [EC:2.1.1.190]
WP_040311151.1	K13566	NIT2, yafV; omega-amidase [EC:3.5.1.3]
WP_040311157.1	K12373	HEXA_B; hexosaminidase [EC:3.2.1.52]
WP_040311159.1	K01186	NEU1; sialidase-1 [EC:3.2.1.18]
WP_040311161.1	K00031	IDH1, IDH2, icd; isocitrate dehydrogenase [EC:1.1.1.42]
WP_040311171.1	K07133	K07133; uncharacterized protein
WP_040311172.1	K03525	coaX; type III pantothenate kinase [EC:2.7.1.33]
WP_040311199.1	K11065	tpx; thioredoxin-dependent peroxiredoxin [EC:1.11.1.24]
WP_040311203.1	K00703	glgA; starch synthase [EC:2.4.1.21]
WP_040311207.1	K06187	recR; recombination protein RecR
WP_040311210.1	K01687	ilvD; dihydroxy-acid dehydratase [EC:4.2.1.9]

WP_040311211.1	K03775	slyD; FKBP-type peptidyl-prolyl cis-trans isomerase SlyD [EC:5.2.1.8]
WP_040311214.1	K07149	K07149; uncharacterized protein
WP_040311215.1	K01190	lacZ; beta-galactosidase [EC:3.2.1.23]
WP_040311222.1	K03655	recG; ATP-dependent DNA helicase RecG [EC:5.6.2.4]
WP_040311226.1	K01206	FUCA; alpha-L-fucosidase [EC:3.2.1.51]
WP_040311238.1	K06142	hlpA, ompH; outer membrane protein
WP_040311244.1	K01921	ddl; D-alanine-D-alanine ligase [EC:6.3.2.4]
WP_040311248.1	K03269	lpxH; UDP-2,3-diacylglucosamine hydrolase [EC:3.6.1.54]
WP_040311249.1	K03584	recO; DNA repair protein RecO (recombination protein O)
WP_040311256.1	K03585	acrA, mexA, adeI, smeD, mtrC, cmeA; membrane fusion protein, multidrug efflux system
WP_040311265.1	K01923	purC; phosphoribosylaminoimidazole-succinocarboxamide synthase [EC:6.3.2.6]
WP_040311287.1	K17836	penP; beta-lactamase class A [EC:3.5.2.6]
WP_040311290.1	K21029	moeB; molybdopterin-synthase adenylyltransferase [EC:2.7.7.80]
WP_040311291.1	K01665	pabB; para-aminobenzoate synthetase component I [EC:2.6.1.85]
WP_040311304.1	K01338	lon; ATP-dependent Lon protease [EC:3.4.21.53]
WP_040311305.1	K02226	cobC, phpB; alpha-ribazole phosphatase [EC:3.1.3.73]
WP_040311308.1	K02227	cbiB, cobD; adenosylcobinamide-phosphate synthase [EC:6.3.1.10]
WP_040311309.1	K02232	cobQ, cbiP; adenosylcobyric acid synthase [EC:6.3.5.10]
WP_040311314.1	K19302	bcrC; undecaprenyl-diphosphatase [EC:3.6.1.27]
WP_040311317.1	K07507	mgtC; putative Mg2+ transporter-C (MgtC) family protein
WP_040311325.1	K02224	cobB-cbiA; cobyrinic acid a,c-diamide synthase [EC:6.3.5.9 6.3.5.11]
WP_040311336.1	K01077	E3.1.3.1, phoA, phoB; alkaline phosphatase [EC:3.1.3.1]
WP_040311340.1	K00705	malQ; 4-alpha-glucanotransferase [EC:2.4.1.25]
WP_040311341.1	K09704	K09704; uncharacterized protein
WP_040311343.1	K01740	metY; O-acetylhomoserine (thiol)-lyase [EC:2.5.1.49]
WP_040311359.1	K19265	gpr; L-glyceraldehyde 3-phosphate reductase [EC:1.1.1.-]
WP_040311361.1	K12573	rnr, vacB; ribonuclease R [EC:3.1.13.1]
WP_040311365.1	K07259	dacB; serine-type D-Ala-D-Ala carboxypeptidase/endopeptidase (penicillin-binding protein 4) [EC:3.4.16.4 3.4.21.-]

WP_040311369.1	K03718	asnC; Lrp/AsnC family transcriptional regulator, regulator for asnA, asnC and gidA
WP_040311370.1	K00945	cmk; CMP/dCMP kinase [EC:2.7.4.25]
WP_040311379.1	K02548	menA; 1,4-dihydroxy-2-naphthoate polyprenyltransferase [EC:2.5.1.74]
WP_040311380.1	K03113	EIF1, SUI1; translation initiation factor 1
WP_040311381.1	K12340	tolC, bepC, cyaE, raxC, sapF, rsaF, hasF; outer membrane protein
WP_040311385.1	K01756	purB, ADSL; adenylosuccinate lyase [EC:4.3.2.2]
WP_040311390.1	K01129	dgt; dGTPase [EC:3.1.5.1]
WP_040311393.1	K03558	cvpA; membrane protein required for colicin V production
WP_040311400.1	K03979	obgE, cgtA, MTG2; GTPase [EC:3.6.5.-]
WP_040311403.1	K12340	tolC, bepC, cyaE, raxC, sapF, rsaF, hasF; outer membrane protein
WP_040311405.1	K21681	bcs1; ribitol-5-phosphate 2-dehydrogenase (NADP+) / D-ribitol-5-phosphate cytidylyltransferase [EC:1.1.1.405 2.7.7.40]
WP_040311413.1	K00566	mnmA, trmU; tRNA-uridine 2-sulfurtransferase [EC:2.8.1.13]
WP_040311416.1	K08303	prtC, trhP; U32 family peptidase [EC:3.4.-.-]
WP_040311417.1	K28561	cerR; ceramide reductase [EC:1.-.-.-]
WP_040311431.1	K07027	K07027; glycosyltransferase 2 family protein
WP_040311432.1	K01662	dxs; 1-deoxy-D-xylulose-5-phosphate synthase [EC:2.2.1.7]
WP_040311438.1	K01613	psd, PISD; phosphatidylserine decarboxylase [EC:4.1.1.65]
WP_040311442.1	K13292	lgt, umpA; phosphatidylglycerol---prolipoprotein diacylglyceryl transferase [EC:2.5.1.145]
WP_040311445.1	K01881	PARS, proS; prolyl-tRNA synthetase [EC:6.1.1.15]
WP_040311452.1	K00950	folK; 2-amino-4-hydroxy-6-hydroxymethyldihydropteridine diphosphokinase [EC:2.7.6.3]
WP_040311458.1	K01809	manA, MPI; mannose-6-phosphate isomerase [EC:5.3.1.8]
WP_040311463.1	K21572	susD; starch-binding outer membrane protein, SusD/RagB family
WP_040311466.1	K03544	clpX, CLPX; ATP-dependent Clp protease ATP-binding subunit ClpX
WP_040311468.1	K09710	ybeB; ribosome-associated protein
WP_040311479.1	K00661	maa; maltose O-acetyltransferase [EC:2.3.1.79]
WP_040311483.1	K06167	phnP; phosphoribosyl 1,2-cyclic phosphate phosphodiesterase [EC:3.1.4.55]

WP_040311484.1	K02343	dnaX; DNA polymerase III subunit gamma/tau [EC:2.7.7.7]
WP_040311485.1	K01465	URA4, pyrC; dihydroorotase [EC:3.5.2.3]
WP_040311490.1	K09765	queH; epoxyqueuosine reductase [EC:1.17.99.6]
WP_040311494.1	K26735	KEA4_5_6, TMCO3, ybaL; K+:H+ antiporter
WP_040311500.1	K01657	trpE; anthranilate synthase component I [EC:4.1.3.27]
WP_040311503.1	K16264	czcD, zitB; cobalt-zinc-cadmium efflux system protein
WP_040311511.1	K09457	queF; 7-cyano-7-deazaguanine reductase [EC:1.7.1.13]
WP_040311517.1	K14155	patB, malY; cysteine-S-conjugate beta-lyase [EC:4.4.1.13]
WP_040311524.1	K01258	pepT; tripeptide aminopeptidase [EC:3.4.11.4]
WP_040311538.1	K21572	susD; starch-binding outer membrane protein, SusD/RagB family
WP_040311541.1	K13280	SEC11, sipW; signal peptidase I [EC:3.4.21.89]
WP_040311547.1	K00677	lpxA; UDP-N-acetylglucosamine acyltransferase [EC:2.3.1.129]
WP_040311562.1	K15727	czcB, cusB, cnrB; membrane fusion protein, heavy metal efflux system
WP_040311564.1	K15725	czcC, cusC, cnrC; outer membrane protein, heavy metal efflux system
WP_040311566.1	K00067	rfbD, rmlD; dTDP-4-dehydrorhamnose reductase [EC:1.1.1.133]
WP_040311573.1	K07263	pqqL; zinc protease [EC:3.4.24.-]
WP_040311594.1	K07566	tsaC, rimN, SUA5, YRDC; L-threonylcarbamoyladenylate synthase [EC:2.7.7.87]
WP_040311605.1	K01081	E3.1.3.5; 5'-nucleotidase [EC:3.1.3.5]
WP_040311611.1	K00566	mnmA, trmU; tRNA-uridine 2-sulfurtransferase [EC:2.8.1.13]
WP_040311630.1	K01633	folB; 7,8-dihydroneopterin aldolase/epimerase/oxygenase [EC:4.1.2.25 5.1.99.8 1.13.11.81]
WP_040311633.1	K02171	blaI; BlaI family transcriptional regulator, penicillinase repressor
WP_050765334.1	K03446	emrB; MFS transporter, DHA2 family, multidrug resistance protein
WP_054856196.1	K07137	K07137; uncharacterized protein
WP_054856198.1	K18138	acrB, mexB, adeJ, smeE, mtrD, cmeB; multidrug efflux pump
WP_054856204.1	K02314	dnaB; replicative DNA helicase [EC:5.6.2.3]
WP_054856242.1	K12340	tolC, bepC, cyaE, raxC, sapF, rsaF, hasF; outer membrane protein
WP_054856254.1	K05770	TSPO, BZRP; translocator protein
WP_054856256.1	K07149	K07149; uncharacterized protein

WP_054856274.1	K03723	mfd; transcription-repair coupling factor (superfamily II helicase) [EC:5.6.2.4]
WP_054856297.1	K00941	thiD; hydroxymethylpyrimidine/phosphomethylpyrimidine kinase [EC:2.7.1.49 2.7.4.7]
WP_054856302.1	K03733	xerC; integrase/recombinase XerC
WP_054856305.1	K25027	btuC; cobalamin transport system permease protein
WP_054856308.1	K00874	kdgK; 2-dehydro-3-deoxygluconokinase [EC:2.7.1.45]
WP_054856341.1	K01714	dapA; 4-hydroxy-tetrahydrodipicolinate synthase [EC:4.3.3.7]
WP_054856356.1	K19049	cslA; chondroitin AC lyase [EC:4.2.2.5]
WP_054856373.1	K01119	cpdB; 2',3'-cyclic-nucleotide 2'-phosphodiesterase / 3'-nucleotidase [EC:3.1.4.16 3.1.3.6]
WP_054856382.1	K09951	cas2; CRISPR-associated protein Cas2
WP_054856410.1	K01190	lacZ; beta-galactosidase [EC:3.2.1.23]
WP_054856433.1	K00600	glyA, SHMT; glycine hydroxymethyltransferase [EC:2.1.2.1]
WP_054856464.1	K00012	UGDH, ugd; UDPglucose 6-dehydrogenase [EC:1.1.1.22]
WP_054856466.1	K01531	mgtA, mgtB; P-type Mg2+ transporter [EC:7.2.2.14]
WP_054856525.1	K19166	higB; mRNA interferase HigB [EC:3.1.-.-]
WP_054858133.1	K01277	DPP3; dipeptidyl-peptidase III [EC:3.4.14.4]
WP_054858134.1	K18138	acrB, mexB, adeJ, smeE, mtrD, cmeB; multidrug efflux pump
WP_054858150.1	K03496	parA, soj; chromosome partitioning protein
WP_054858217.1	K01952	PFAS, purL; phosphoribosylformylglycinamidine synthase [EC:6.3.5.3]
WP_054858255.1	K19166	higB; mRNA interferase HigB [EC:3.1.-.-]
WP_054861734.1	K16066	ydfG; 3-hydroxy acid dehydrogenase / malonic semialdehyde reductase [EC:1.1.1.381 1.1.1.-]
WP_054861760.1	K00937	ppk1; polyphosphate kinase [EC:2.7.4.1]
WP_054861807.1	K07316	mod; adenine-specific DNA-methyltransferase [EC:2.1.1.72]
WP_081446907.1	K01934	MTHFS; 5-formyltetrahydrofolate cyclo-ligase [EC:6.3.3.2]
WP_081446910.1	K01607	pcaC; 4-carboxymuconolactone decarboxylase [EC:4.1.1.44]
WP_081446913.1	K03741	arsC; arsenate reductase (thioredoxin) [EC:1.20.4.4]
WP_081446928.1	K01206	FUCA; alpha-L-fucosidase [EC:3.2.1.51]
WP_081446929.1	K07496	K07496; putative transposase
WP_081446947.1	K12340	tolC, bepC, cyaE, raxC, sapF, rsaF, hasF; outer membrane protein
WP_081446957.1	K25027	btuC; cobalamin transport system permease protein

WP_118400320.1	K12340	tolC, bepC, cyaE, raxC, sapF, rsaF, hasF; outer membrane protein
WP_118400804.1	K04095	fic, FICD, HYPE; cell filamentation protein, protein adenylyltransferase [EC:2.7.7.108]
WP_155819018.1	K02622	parE; topoisomerase IV subunit B [EC:5.6.2.2]
WP_155819152.1	K17641	bxlA; beta-xylosidase
WP_156321737.1	K00012	UGDH, ugd; UDPglucose 6-dehydrogenase [EC:1.1.1.22]
WP_162231583.1	K02004	ABC.CD.P; putative ABC transport system permease protein
WP_162231587.1	K03088	rpoE; RNA polymerase sigma-70 factor, ECF subfamily
WP_169306508.1	K12340	tolC, bepC, cyaE, raxC, sapF, rsaF, hasF; outer membrane protein
WP_173585527.1	K13038	coaBC, dfp; phosphopantothenoylcysteine decarboxylase / phosphopantothenate---cysteine ligase [EC:4.1.1.36 6.3.2.5]
WP_185114289.1	K03686	dnaJ; molecular chaperone DnaJ
WP_187326036.1	K03281	clcA, clcB, CLC-E, CLC-F; chloride channel protein, CIC family
WP_187326038.1	K01599	hemE, UROD; uroporphyrinogen decarboxylase [EC:4.1.1.37]
WP_204083345.1	K02172	blaR1; bla regulator protein blaR1
WP_204083355.1	K05349	bglX; beta-glucosidase [EC:3.2.1.21]
WP_204083356.1	K21572	susD; starch-binding outer membrane protein, SusD/RagB family
WP_204083357.1	K21572	susD; starch-binding outer membrane protein, SusD/RagB family
WP_204083361.1	K07484	K07484; transposase
WP_204083369.1	K26937	dinF, mepA, vmrA; MATE family, multidrug efflux pump
WP_204083370.1	K07484	K07484; transposase
WP_204083371.1	K07484	K07484; transposase
WP_204083382.1	K02342	dnaQ; DNA polymerase III subunit epsilon [EC:2.7.7.7]
WP_204083387.1	K01012	bioB; biotin synthase [EC:2.8.1.6]
WP_204083388.1	K02233	E2.7.8.26, cobS, cobV; adenosylcobinamide-GDP ribazoletransferase [EC:2.7.8.26]
WP_204083389.1	K07484	K07484; transposase
WP_204083394.1	K11537	xapB; MFS transporter, NHS family, xanthosine permease
WP_204083395.1	K07263	pqqL; zinc protease [EC:3.4.24.-]
WP_246497753.1	K06889	K06889; uncharacterized protein
WP_246497775.1	K19294	algI; alginate O-acetyltransferase complex protein AlgI
WP_246497787.1	K07452	mcrB; 5-methylcytosine-specific restriction enzyme B [EC:3.1.21.-]

WP_246497789.1	K01206	FUCA; alpha-L-fucosidase [EC:3.2.1.51]
WP_246497798.1	K00265	gltB; glutamate synthase (NADPH) large chain [EC:1.4.1.13]
WP_246497808.1	K03574	mutT, NUDT15, MTH2; 8-oxo-dGTP diphosphatase [EC:3.6.1.55]
WP_246497814.1	K01130	atsA, aslA; arylsulfatase [EC:3.1.6.1]
WP_246497817.1	K06131	clsA_B; cardiolipin synthase A/B [EC:2.7.8.-]
WP_246497818.1	K26937	dinF, mepA, vmrA; MATE family, multidrug efflux pump
WP_370847634.1	K01442	cbh; choloylglycine hydrolase [EC:3.5.1.24]
WP_370847663.1	K23514	fecI, prhI; RNA polymerase sigma-19 factor, ECF subfamily
WP_370848103.1	K16089	TC.FEV.OM2, cirA, cfrA, hmuR; outer membrane receptor for ferrienterochelin and colicins
WP_373920400.1	K01190	lacZ; beta-galactosidase [EC:3.2.1.23]
WP_373920405.1	K13789	GGPS; geranylgeranyl diphosphate synthase, type II [EC:2.5.1.1 2.5.1.10 2.5.1.29]
WP_373920412.1	K01448	amiABC; N-acetylmuramoyl-L-alanine amidase [EC:3.5.1.28]
WP_373920414.1	K12308	bgaB, lacA; beta-galactosidase [EC:3.2.1.23]
